# Supplementary material for: Genomic analysis of two phlebotomine sand fly vectors of Leishmania from the New and Old World
Source: PLoS Negl Trop Dis. 2023 Apr 12;17(4):e0010862. doi: 10.1371/journal.pntd.0010862 (PMC10138862; doi:10.1371/journal.pntd.0010862)
Supplement: S1 Methods — (DOCX) [file pntd.0010862.s001.docx]

**Genomic Analysis of Two Phlebotomine Sand Fly Vectors of *Leishmania* from the New and Old World**

**Supplemental Methods:**

Manual Annotation Methods

*Transposable Elements*

*Long Terminal Repeats*

Psiblast was used to construct reverse position specific matrices from the deducted coding sequences from transposable elements found in the TEFAM and REPBASE [1] databases. The sand fly genomes (in pieces of 50 kb containing 10 kb of the previous fragment) were queried against this database using the tool RPSblast [2] with an e-value cutoff of 1e-15. The coordinates of the matches having > 800 nt were extended by 500 nt to include flanking regions. The final file containing the genome scaffold name, plus n1 and n2 extended coordinates was sorted and the coordinates that had overlap were fused. This compacted coordinate table was finally used to retrieve the putative transposable elements as a fastA file. The sequences were trimmed at the terminal inverted repeat (TIR) sequences or alternatively, at the coding sequence when repeats were not found.

The domains were classified according to the Conserved Domain Database [3] and the full-length Reverse Transcriptase-Riboneuclease H regions used to perform phylogenetic analysis that permitted the further classification of the sequences into families.

The classification of the different long terminal repeat (LTR) superfamilies was based on phylogenetic analysis. The full-length Reverse Transcriptase domains were aligned to reference sequences belonging to the main LTR superfamilies by the Mafft algorithm as implemented in <http://mafft.cbrc.jp/alignment/server/> [4] and manually edited in
MEGA5 [5].

*Non-Long Terminal Repeats*

Using a TEseeker [6] we searched for putative non-LTR elements. To annotate these putative sequences we performed Phylo tree building for each putative element verses previously known published representative non-LTRs from other insect genomes, and assigned a clade. We also clustered using ClustalW [7] all putative and all annotated elements with the representatives of other organisms within the clade and overall. We considered protein sequences that were 80% identical as one element and making manual corrections we built a consensus sequence of the 80% identical elements. We then used this consensus sequence to perform a final count of the copy number of each element in the genome, and to estimate percent genome coverage.

*Miniature Inverted-repeat Transposable Elements*

RepeatModeler [8] output that were less than 500 bp were used as query to run BLAST against the genome assembly. Top 20 hits were retrieved together with their flanking sequences (500 bp on each side). These sequences were aligned using ClustalW [7] to reveal TE boundaries, TIRs and target site duplications that are characteristics of miniature inverted-repeat transposable elements (MITEs). Self-blast of the initial MITE candidates were performed to remove redundancy using a cut-off of overall 80% identity. To discover short interspersed nuclear elements (SINEs), sequences that are less than 500 bp and showed similarity to tRNAs or other polymerase III promoters were selected. Element boundaries, target site duplications and tandem repeats at the 3’ ends that are characteristics of SINEs were also analyzed by Clustal alignment of top 20 hits.

*Digestion Genes*

*Peptidases, GHF13 and GHF18*

The Peptidase and Glycoside Hydrolase Families (GHF) (defined as described in the MEROPS and CAZy databases, respectively) in sand flies genomes were characterized using FAT software [9], which integrates HMMER (http://hmmer.janelia.org/) and BLAST+ tools [10] to filter the initial dataset and perform automatic annotation. The filter step used the HMG-box conserved domain (Pfam code varied between families) to identify and extract only proteins containing such a domain in both datasets.

*N-acetylhexosaminidases*

VectorBase [11-13] was screened for sand fly n-acetylhexosamisnidases through tBlastn searches against *Tribolium* *castaneum* homologs [14]. Genbank Accession numbers: *Drosophila melanogaster* (DmNAG1: NP_523924; DmNAG2: NP_525081; and DmFDL: NP_725178), *Anopheles gambiae* (AgNAG1: XP_315391; AgNAG2: XM_307483; AgFDL: XP_308677; and AgHEX: XM_319210), *Aedes aegypti* (AaNAG1: EAT43909; AaNAG2: EAT40440; AaFDL: EAT36388; and AaHEX: EAT43655), and *T. castaneum* (TcNAG1: ABQ95982; TcNAG2: ABQ95983; TcNAG3: ABQ95984; TcFDL: ABQ95985; TcHEX1: XM_970563; TcHEX2: XM_970567; and TcHEX3: XM_970565). Conserved domain architecture was identified through CDD, Prosite, and Pfam database searches.

*Chitin deacetylases*

VectorBase was screened for sand fly chitin deacetylase (CDA) through tBlastn searches against *T. castaneum* homologs [15]. Genbank Accession numbers: *T. castaneum* (TcCDA1: ABU25223; TcCDA2: ABU25224; TcCDA3: ABW74145; TcCDA4: ABW74146; TcCDA5: ABW74147; TcCDA6: ABW74149; TcCDA7: ABW74150; TcCDA8: ABW74151; and TcCDA9: ABW74152), *An. gambiae* (AgCDA1: EAA00275; AgCDA2: EAA43313; AgCDA3: EAA12484; AgCDA4: EAA06323; and AgCDA5: EAA12207); *Ae. aegypti* (AeCDA1: EAT45316; AeCDA2: EAT45315; AeCDA3: EAT42807; and AeCDA4: EAT42983), and *D. melanogaster* (DmCDA1: AGB94755; DmCDA2: AAF49121; DmCDA3: NP_609806; DmCDA4: AAF50937; DmCDA5: ABV53594; and DmCDA9: AHN56315). Conserved domain architecture was identified through CDD, Prosite, and Pfam database searches.

*Peritrophin-like proteins*

VectorBase [11-13] was screened for *Lutzomyia longipalpis* peritrophins by carrying out tBlastn searches against peritrophin-like proteins from many insects, for instance: *Lucilia cuprina* (AAC37261, AAC70784, AAK01058, AAB38414, and AAB70878); *An. Gambiae* (AAV31069); *Chrysomya bezziana* (AAK01057, AAB86623, and AAD25103); *D. melanogaster* (NP_524982 and NP_524983); *Trichoplusia ni* (AAC47557 and AAC47556); *Armigeres* subalbatus (AY439786); *Ae. Aegyti* (AAL05409, AAM94157, AAM94156, AAM94155, and AAM94154); and the sand fly peritrophins identified in cDNA libraries [16, 17]. This initial screening gave rise to 10 unique *Lu. longipalpis* peritrophins. Such sequences were translated, and their protein sequences were used to further search Bfor novel sand fly peritrophins. Genbank Accession numbers for proteins displayed in the phylogenetic tree: *T. castaneum* peritrophin access numbers were retrieved from [18] whereas available sand fly peritrophin access numbers were retrieved from [16, 17]. Conserved domain architecture was identified through CDD, Prosite, and Pfam database searches.

*Immunity Genes*

*JAK/STAT*

OrthoMCL was used to search for orthologous genes between the 2 sand fly species and 6 other insect species (*D. melanogaster, Anophles albimanus, An. gambiae, Ae. aegypti, Culex quinquefasciatus*, *Rhodnius prolixus*). The genome sequences for these organisms were downloaded from VectorBase [11-13], with the exception of *D. melanogaster*, which was downloaded from FlyBase [19]. Following the OrthoMCL comparisons, a list of known immunity genes and ID’s were downloaded from FlyBase for *D. melanogaster*. There were a total of 5 JAK/STAT genes found using this method; Hopscotch, Domeless, STAT92E, Upd3, SOCS36E. The ID's were used to search for the OrthoMCL clusters for the 5 JAK/STAT genes. From the OrthoMCL gene cluster ID, it was possible to find the corresponding ID’s for the 2 sand fly species. 5 VectorBase ID’s were found in each sand fly species for the 5 JAK/STAT genes. VectorBase searches were performed to retrieve the sand-fly ID’s, each gene was downloaded to assess the quality of the gene model on VectorBase. In order to assess the quality of the gene model, Artemis [20] was used. The gene models were checked to make sure that that the coding sequences started and ended with the correct codons. In addition, the coding sequences were checked to make sure that there were no stop codons in the coding sequences themselves. All of the gene models were of good quality and everything was in the right place. Once the gene quality had been assured, the gene ID’s could then be confirmed.

To find more JAK/STAT genes, the Insect Immunity Database (Bordenstein Lab, NSF DEB-1046149) was used. This website contains a list of immunity genes from different insects, the JAK/STAT gene list has been mainly compiled using *D. melanogaster*. two genes were found from this list that were also present in our OrthoMCL gene clusters, these were; p38b, and mekk1. The genes were again found using VectorBase and the gene models were confirmed using Artemis [20].

*Prophenoloxidase genes*

The same OrthoMCL gene clusters were used as in the JAK/STAT gene annotations. Using the FlyBase immunity gene list, we found three prophenoloxidase genes. These three genes turned out to all belong to the same gene cluster. The gene models were downloaded for all of the three prophenoloxidase genes and they were assessed using Artemis [20]. All were confirmed to be good quality. Another phylogenetic tree is being created using these genes, more phenoloxidase genes are being searched for currently.

*Toll Signaling Genes*

The annotation was focused on the core signaling molecules in the Toll pathway and also on the upstream pathogen recognition receptors including peptidoglycan recognition proteins and gram-negative bacteria-binding proteins. This manual annotation started by using blastp against the two sand fly genomes from Vectorbase with the *Drosophila* protein sequences of the core Toll signaling genes. The *Drosophila* genes and protein sequences were obtained from FlyBase (Toll: FBgn0262473; PGRP-SA: FBgn0030310; GNBP1: FBgn0040323; Tube: FBgn0003882; Pelle: FBgn0010441; Myd88: FBgn0033402; Cactus: FBgn0000250; Dorsal: FBgn0260632). Gene models of the top hits from the blast were checked by Artemis [20] to ensure the correct annotation of coding region. Subsequently the protein (transcribed) sequences of the hits were used to align with *Drosophila* counterpart by ClustalW [7]. A score above 20 was set as the arbitrary threshold to confirm it was a ‘true’ homologue. Furthermore, SMART [21] was used to confirm that the characteristic protein domains essential for the gene function are present in the sand fly homologues.

*IMD/ROS genes*

For manual annotation, blast programs were used with IMD/ROS core genes from *Drosophila* or in-house sequences from *Lu. longipalpis* as query. The *Drosophila* genes and protein sequences were obtained from Flybase. Gene models from the blast highest hits were checked by Artemis [20] to ensure the correct annotation of coding region.

*Galactose-binding proteins*

VectorBase was screened for sand fly galectins through tBlastn searches against fly, mosquito, and sand fly homologs retrieved from Genbank. Genbank accession numbers: *Crassostrea virginica* (CVgalec: ABG75998.1), *Ae. aegypti* (AeGal1: XP_001656933; AeGal2: XP_001655870; AeGal3: EAT44461; AeGal5: XP_001657305; AeGal6: EAT43258; AeGal8: XP_001662146; AeGal11: EAT44861; AeGal12: EAT38239; AeGal13: EAT38241; AeGal14: EAT38240; and AeGal_ XP_001650704: XP_001650704), *An. gambiae* (AgGal1: EAA14815;AgGAl2: EAA06231; AgGAl3: EAA10348; AgGal4: EAL41552; AgGal5: EAA05052; AgGAl6: EAA13122; AgGal7: EAA13132; AgGal8: EAA09838; AgGal9: EGK97239; and AgGal10: EAL38639), *D. melanogaster* (DmGalA: AAF51564; DmGalB: ACZ94129; DmGalD: AFH03490; DmGalE: ACZ94130; DmGalF: AGB92325; DmGal_CG5335: AAF57667; DmGal_CG11374: AAF51563; DmGal_peroxin23: AAF49038; DmGal_CG13950: AAF51443; and DmGal_CG14879: AHN57362), *Ph. papatasi* (PpGalecA: AY538600), *Lu. longipalpis* (LuloGalecR: ABV60341), and *Biomphalaria glabrata* (BgGal_ABQ09359: ABQ09359). Conserved domain architecture was identified through CDD, Prosite, and Pfam database searches.

*MAP Kinases*

A bidirectional BLAST method was applied to identify new orthologous genes belonging to the ERK, JNK and p38 MAPKs pathways. All RefSeq proteins of *Homo sapiens*, *An. gambiae*, *D. melanogaster* and *Caenorhabditis elegans* were downloaded from Genbank and a set of 79 MAPKs was made by selecting representative amino acid sequences from these species. Set of homologue sequences used as query: NP_001036065.1, NP_003945.2, XP_310236.3, NP_109587.1, NP_005912.1, NP_003609.2, XP_307879.4, XP_309868.4, XP_316357.4, XP_316502.4, XP_318144.4, XP_322064.4, NP_572458.3, NP_477163.1, NP_477353.1, NP_477361.1, NP_491683.1, NP_491087.1, NP_492620.1, NP_501365.1, NP_504721.1, NP_509682.1, NP_524080.1, NP_511098.1, NP_002742.3, NP_620602.2, NP_620448.1, NP_620581.1, NP_620637.1, NP_620407.1, NP_002743.3, NP_660143.1, NP_660186.1, NP_002437.2, NP_663304.1, NP_005195.2, NP_004570.2, NP_723541.1, NP_727335.1, NP_610817.1, NP_611399.2, NP_741431.1, NP_741537.1, NP_477089.3, NP_001001671.3, NP_649137.3, NP_006292.3, NP_872069.1, NP_004324.2, NP_001193733.1, NP_001229243.1, NP_001229488.1, XP_310813.5, NP_976226.1, NP_650750.4, NP_001645.1, NP_002410.1, NP_002745.1, NP_002739.1, NP_002871.1, NP_003001.1, NP_995972.1, NP_996277.1, NP_004663.3, NP_002960.2, NP_149132.2, XP_005248576.1, XP_005250103.1, XP_005258356.1, NP_002746.1, XP_006715545.1, XP_006723285.1, NP_009112.1, NP_001021270.1, NP_001022584.1, NP_741430.3, NP_001024971.1, NP_002737.2, NP_620590.2.

This query dataset was blasted against the *Ph. papatasi* and *Lu. longipalpis* genome. The genomic regions with highest similarity to MAPKs were extracted and blasted against all downloaded RefSeq proteins. Only the genomic regions which matched to MAPKs in the second blast round were considered as a MAPK gene locus. The exons were identified by the comparison of the genes found to the RNA-seq data assembled using different methodologies. The RNA-seq assemblies were also screened by a similar bidirectional blast approach.

*TGF-beta*

Manual annotation was performed by identifying *D. melanogaster* ortholog genes related to TGF-beta molecules from FlyBase (<http://flybase.org>; dcapentaplegic: [FBgn0000490](http://flybase.org/reports/FBgn0000490.html); maverick: [FBgn0039914](http://flybase.org/reports/FBgn0039914.html) ; mother against decapentaplegic: [FBgn0011648](http://flybase.org/reports/FBgn0011648.html); smad on X: [FBgn0025800](http://flybase.org/reports/FBgn0025800.html); punt: [FBgn0003169](http://flybase.org/reports/FBgn0003169.html) ; saxophone: [FBgn0003317](http://flybase.org/reports/FBgn0003317.html); baboon: [FBgn0011300](http://flybase.org/reports/FBgn0011300.html); thickveins: [FBgn0003716](http://flybase.org/reports/FBgn0003716.html); tolloid: [FBgn0003719](http://flybase.org/reports/FBgn0003719.html); fusel: [FBgn0039932](http://flybase.org/reports/FBgn0039932.html); inwardly rectifying potassium channel 2: [FBgn0039081](http://flybase.org/reports/FBgn0039081.html); supernumerary limbs: [FBgn0267841](http://flybase.org/reports/FBgn0267841.html); spichthyin: [FBgn0032451](http://flybase.org/reports/FBgn0032451.html); short gastrulation: [FBgn0003463](http://flybase.org/reports/FBgn0003463.html)). These sequences were used as a query using BLAST search tools against the two sand fly genomes from VectorBase. Gene models identified in the sand fly genomes were checked by Artemis [20] or Web Apollo [22] and metadata was added.

*Circadian Rhythm Related Gene Families*

Using a bioinformatic approach, we identified several genes involved in the control of circadian rhythms, behaviour and sensory ecology in other insect species. Orthologous sequences, mostly from *D. melanogaster*, *An. gambie*, *Ae. aegypti*, and *C. quinquefasciatus* were used as a query to perform HMMER [23], BLASTP, and TBLASTN searches on the *Lu. longipalpis* and *Ph. papatasi* genomes and on their predicted protein sets. Subsequently, some gene models were improved manually or using gene prediction programs such as Augustus [24] and Fgenesh+ [25]. Final gene models have been created using Artemis [20]. The clock and behaviour related genes identified and their main characteristics are detailed in S1 and S2 Tables.for *Lu. longipalpis* and *Ph. papatasi*, respectively.

*cycle* (*cyc*), *Clock* (*Clk*), and *vrille* (vri) genes were experimentally obtained. We first designed degenerate primers in conserved regions of vertebrate and invertebrate ortholog genes (*Mus musculus*, *Gallus gallus*, *Danio rerio*, and *D.* *melanogaster*; GenBank accession numbers AAC53200, AAF26365, AAD27749, and AAF50516). Based on these first sequences, new specific primers were made to obtain further sequences. The 5’ and 3’ ends were obtained using RACE techniques [26]. cDNA sequences were then used as query to search and edit the *Lu. longipalpis* and *Ph. papatasi* gene models automatically annotated.

*Lu. longipalpis* and *Ph. papatasi* genes that belong to serine/threonine phosphatase (Pp1-alpha, Pp1-beta, Pp2-alpha, Pp2-beta, Pp4, and PpV-6, and Pp7) and photolyase superfamilies (cry-1, cry-2, phr, (6-4) phr) were differentiated by performing phylogenetic analyses using vertebrate and invertebrate ortholog genes. To do so, alignments of vertebrate and invertebrate ortholog sequences were performed in ClustaL X [7], manually edited in Jalview v2.6.1 [27] and subsequently aligned using G-INS-I strategy in MAFFT [28]. For the phylogenetic reconstruction, different evolutionary models (JTT, LG, DCMut, MtREV, MtMam, MtArt, Dayhoff, WAG, RtREV, CpREV, Blosum62, and VT) were tested using the ProtTest v2.4 [29]. Finally, maximum likelihood trees with 1,000 bootstrap pseudo-replicates of the original data were built in MEGA6 [30].

In the case of the pickpocket (PPK) and transient receptor potential (TRP) cation channel protein families, interative searches were performed with each new obtained protein sequence as query until no new genes were identified in each major subfamily or lineage. Once gene modelling process was finished, the number of predicted transmembrane domains in sand fly TRP and PPK sequences was established using TOPCONS [31]. Besides, functional domains were identified using Pfam v27.0 database [32]. Finally, a phylogenetic analysis with sequences from these protein families and those from *D. melanogaster* was performed. TRP sequences from *Drosophila* melanogaster were obtained from Peng et al. [33]. In the case of PPKs, fruit fly sequences were obtained from Zelle et al. [34]. To build the TRP and PPK phylogenetic trees, we aligned and edited the sequences the same way as described above.

*RNA Genes and MicroRNAs*

Identification and characterization of RNAi genes - *D. melanogaster* RNA interference core proteins involved in siRNA, miRNA and piRNA pathways were used to search for similarity using BLASTP against all predicted polypeptide genes in *Lu. longipalpis* and *Ph. papatasi* genomes. Identification of sand fly predicted polypeptides was confirmed by similarity searches against annotated proteins in the *Drosophila* genome using BLASTPh. RNAi proteins identified by this strategy were analyzed to determine size and domain organization with InterProScan and gene were refined using SoftBerry and manual curation. Identification of microRNAs and piRNAs - Small RNA libraries were constructed from adult female *Lu. longipalpis* and deep sequenced using the Illumina platform. Small RNAs were mapped against the *Lu. longipalpis* genome using Bowtie. miRNAs were identified using miRDeep2 [35]. Candidate precursors were manually curated using 4 different criteria: (1) Size distribution of mapped reads on precursors, (2) Homogeneity of 5’ end of mapped reads, (3) detection in more than one library and (4) conservation in other insects. To identify piRNA clusters, *Luztomya* scaffolds were separated in 2000 nt bins using in-home perl scripts that were used as reference for mapping reads libraries using Bowtie [36]. 2000 nt bins with a profile of mapped reads showing size distribution between 24 and 30 nt were selected. These were analyzed for ping-pong signature [37], nucleotide enrichment (weblogo) and offset between reads mapped in positive and negative strands.

*G-Protein Coupled Receptors*

A novel classifier pipeline was utilized to identify the GPCRs from the *Ph. papatasi* and *Lu. longipalpis* genome assemblies at VectorBase using the set of known GPCR peptides from *Ae. aegypti, An. gambiae, Apis mellifera, D. melanogaster, H. sapiens,* and *Pediculus humanus* [38]. The identified sand fly GPCR sequences were aligned using BLAST against the non-redundant protein sequences (nr) in NCBI. Putative functions were assigned based on the best and the most informative BLAST hits, in most of the cases the later was from *D. melanogaster*. The sand fly sequences were aligned with homologous genes, mostly from insects used in the classifier pipeline, using MultAlin [39] and trees from Clustal Omega [40]. The sand fly gene models were manually corrected using the annotation tool Apollo. Identified and manually annotated GPCRs were used to search (tBLASTn) the sand fly genome scaffolds for additional GPCR genes. When available, the gene models were confirmed using transcript evidence from Expressed Sequence Tags (ESTs) and RNA sequencing (RNAseq).

*Cytochrome P450s*

To identify all potential members of the cytochrome P450 superfamily we aligned the predicted gene sets of *Lu. longipalpis* (LlonJ1.6) and *Ph. papatasi* (PpapI1.6) to a reference collection of 2,942 curated CYPs from a wide variety of arthropods [41] using BLAST v2.10.0+ [10]. Since it is known that automated gene prediction methods quite frequently contain mistakes we proceeded to manually curate the automatically predicted CYP genes. To this end, we mapped publicly available *Lu. longipalpis* (SRR535765) and *Ph. papatasi* (ERR3714261, ERR3714267, ERR3714268) publicly available RNAseq data on the reference genomes using Hisat2 v2.1.0 [42] with parameters “--dta-cufflinks”. Subsequently, Bam2wig v3.0.1 was used with default parameters to generate BigWig files, in order to obtain transcription levels along the genome [43]. The BigWig files were then loaded as separate tracks in a locally deployed instance of the Apollo genome browser (v2.6.0) [22] that contained the sand fly genome sequences as well as the automatic gene predictions. We also identified CYP genes that were missed by automatic gene prediction using tblastn searches of the reference CYP set against each genome assembly. The Apollo genome browser was used for manual curation of the P450 genes.

A phylogenetic analysis of the *Lu. longipalpis* and *Ph. papatasi* CYPs was conducted using the curated CYPome of the major malaria vector *An. gambiae [41]* as reference. More specifically, we first aligned the amino acid sequence of the CYPomes of the three species using MAFFT v7.490 [44] with the “auto” parameter. Next, we trimmed the alignment using Trimal v1.2rev59 [45] with parameters “-gt 0.50”. IQ-TREE 1.6.12 [46] was used with parameters “*-alrt 5000 -bb 5000 -m MFP*” to reconstruct a maximum likelihood phylogeny with 5,000 bootstraps using the ultrafast bootstrap algorithm UFBoot2 [47]. Amino-acid model selection was performed using ModelFinder [48]. The human CYP51A1 was used as an outgroup. Finally, tree visualization and post-processing was performed using Evolview v3 [49].

*Salivary Protein Genes*

Forty-nine *Ph. papatasi* and 35 *Lu. longipalpis* putative salivary genes deposited at NCBI [50] were mapped to the sand fly assemblies using BLAST.

*Heat Shock Protein Genes*

Comparison of *D. melanogaster* proteins with GO terms relating to heat shock and response to hypoxia were compared by BLAST to the scaffolds and predicted genes in *Lu. longipalpis* and *Ph. papatasi*. Genes that were identified as possible orthologs were then compared by BLAST to the arthropod-specific BLAST database using CLC Genomics Workbench 7.

*Cuticular Protein Genes*

Sequence motifs characteristic of various families of cuticle proteins [51] were blasted (tBLASTn) [52] against the official gene set for *Lu. longipalpis* (LlonJ1.4) and *Ph. papatasi* (Ppal1.4), which were obtained from VectorBase (http://vectorbase.org). Potential cuticle proteins were further analyzed with CutProtFam-Pred, a tool for predicting cuticular protein families described by Ioannidou et al. [53], to assign genes to gene families. To find the closest putative homolog to cuticle protein genes from *Lu. longipalpis* and *Ph. papatasi*, genes were searched against the official gene sets for *Ae. aegypti* and *An. gambiae* downloaded from VectorBase as well as *D. melanogaster* genes obtained from FlyBase (http://flybase.org). The gene with the lowest e-value was considered the closest putative homolog.

*Vitamin Metabolism Genes*

Known protein sequences associated with vitamin metabolism from *D. melanogaster* were acquired through batch download and compared using BLASTagainst peptides from *Ph. papatasi* and*Lu. longipalpis* to identify the highest e-value and bit score matches through the use of CLC Genomics Workbench 7. These peptides from *Ph. papatasi* and *Lu. longipalpis* were subsequently compared against databases for *An. gambiae*and *Ae. aegypti*to identify specific orthologs and to confirm their identity.

*Hormonal Signaling Genes*

*D.melanogaster* proteins with GO terms relating to insulin signaling processes were compared to the hypothetical scaffolds of the two sand fly species *Lu. longipalpis* and *Ph. papatasi* using BLAST. Predicted genes that were identified as possible orthologs were then compared by BLAST to the arthropod-specific BLAST database using CLC Genomics Workbench 7 to validate the identity of each gene.

*Antioxidant Genes*

Comparison of *D. melanogaster* proteins with GO terms relating to antioxidant activity and response to oxidative stress were compared using BLAST to predicted genes of the two sand fly species *Lu. longipalpis* and *Ph. papatasi* allowed for identification of orthologs in each species. Predicted gene that were identified as orthologs were then compared by BLAST to an arthropod-specific BLAST database using CLC Genomics Workbench 7 for gene identity validation.

*Aquaporin Genes*

Aquaporins were identified based upon BLAST comparison to those from mosquitoes and other higher flies [54]. Putative aquaporin genes were subsequently compared to an arthropod-specific BLAST database using CLC Genomics Workbench 7 to validate each as an AQPh.

*Novel Viruses*

Two strategies were used to investigate the possible presence of novel viruses in the sand fly genomes. First, reads were aligned onto known viral sequences. The Taxonomy Browser of NCBI was used to select 1795534 viral sequences from GenBank. 1791477 of these proved suitable for use. The GS Reference Mapper 3.0 software (454 Life Sciences) was used to align the *Lutzomyia* and *Phlebotomus* *.sff files onto the downloaded viral GenBank fraction. Reads of less than 75 bases in length were discarded and the hit match threshold was set at 85% identity. *Lutzomyia* Illumina *.fq files were first groomed using read cleaner (Gatherer, unpublished) and then aligned onto the same downloaded viral GenBank fraction using BWA [55] and Bowtie [36] implemented in valet (Gatherer, unpublished). Output was viewed in Tablet [56].

Secondly, the NCBI Taxonomy Browser was used to search for a reference genome from each family of viruses as defined by the International Union for Taxonomy of Viruses (ICTV), retrieving 101 reference genomes. BLASTN was then used to search the *Lutzomyia* and *Phlebotomus* assembled transcript sets for homologous sequences. A list was compiled of hits at expectation threshold of <10^-3^ and match longer than 100 nucleotides, produced a total of 11 non-redundant hits for *Lutzomyia* and 6 for *Phlebotomus*. Each candidate hit was then used to search the whole of GenPept by BLASTX to determine its nearest protein relative. In addition, BLASTN was used with an expectation threshold of <10^-3^ to match individual genome contigs to the viral GenBank fraction described above. Genome contigs were also BLASTed against bracoviral and retroviral fractions of GenBank.

**References**

1. Jurka J, Kapitonov VV, Pavlicek A, Klonowski P, Kohany O, Walichiewicz J. Repbase Update, a database of eukaryotic repetitive elements. Cytogenet Genome Res. 2005;110(1-4):462-7. doi: 10.1159/000084979. PubMed PMID: 16093699.

2. Altschul SF, Gish W, Miller W, Myers EW, Lipman DJ. Basic local alignment search tool. J Mol Biol. 1990;215(3):403-10. doi: 10.1016/S0022-2836(05)80360-2. PubMed PMID: 2231712.

3. Marchler-Bauer A, Lu S, Anderson JB, Chitsaz F, Derbyshire MK, DeWeese-Scott C, et al. CDD: a Conserved Domain Database for the functional annotation of proteins. Nucleic Acids Res. 2011;39(Database issue):D225-9. Epub 20101124. doi: 10.1093/nar/gkq1189. PubMed PMID: 21109532; PubMed Central PMCID: PMCPMC3013737.

4. Katoh K, Misawa K, Kuma K, Miyata T. MAFFT: a novel method for rapid multiple sequence alignment based on fast Fourier transform. Nucleic Acids Res. 2002;30(14):3059-66. doi: 10.1093/nar/gkf436. PubMed PMID: 12136088; PubMed Central PMCID: PMCPMC135756.

5. Tamura K, Peterson D, Peterson N, Stecher G, Nei M, Kumar S. MEGA5: molecular evolutionary genetics analysis using maximum likelihood, evolutionary distance, and maximum parsimony methods. Mol Biol Evol. 2011;28(10):2731-9. Epub 20110504. doi: 10.1093/molbev/msr121. PubMed PMID: 21546353; PubMed Central PMCID: PMCPMC3203626.

6. Kennedy RC, Unger MF, Christley S, Collins FH, Madey GR. An automated homology-based approach for identifying transposable elements. BMC Bioinformatics. 2011;12:130. Epub 20110503. doi: 10.1186/1471-2105-12-130. PubMed PMID: 21535899; PubMed Central PMCID: PMCPMC3107183.

7. Larkin MA, Blackshields G, Brown NP, Chenna R, McGettigan PA, McWilliam H, et al. Clustal W and Clustal X version 2.0. Bioinformatics. 2007;23(21):2947-8. doi: 10.1093/bioinformatics/btm404. PubMed PMID: 17846036.

8. RepeatModeler [Internet]. 2010. Available from: <http://www.repeatmasker.org/RepeatModeler/>.

9. Seabra-Junior E, Souza E, Mesquita R. FAT - functional Analysis Software. 2011.

10. Camacho C, Coulouris G, Avagyan V, Ma N, Papadopoulos J, Bealer K, et al. BLAST+: architecture and applications. BMC Bioinformatics. 2009;10:421. Epub 20091215. doi: 10.1186/1471-2105-10-421. PubMed PMID: 20003500; PubMed Central PMCID: PMCPMC2803857.

11. Giraldo-Calderón GI, Harb OS, Kelly SA, Rund SS, Roos DS, McDowell MA. VectorBase.org updates: bioinformatic resources for invertebrate vectors of human pathogens and related organisms. Curr Opin Insect Sci. 2021;50:100860. Epub 20211203. doi: 10.1016/j.cois.2021.11.008. PubMed PMID: 34864248.

12. Giraldo-Calderon GI, Emrich SJ, MacCallum RM, Maslen G, Dialynas E, Topalis P, et al. VectorBase: an updated bioinformatics resource for invertebrate vectors and other organisms related with human diseases. Nucleic Acids Res. 2015;43(Database issue):D707-13. Epub 2014/12/17. doi: 10.1093/nar/gku1117. PubMed PMID: 25510499; PubMed Central PMCID: PMCPMC4383932.

13. Megy K, Emrich SJ, Lawson D, Campbell D, Dialynas E, Hughes DST, et al. VectorBase: improvements to a bioinformatics resource for invertebrate vector genomics. Nucleic acids research. 2012;40:D729-34. doi: 10.1093/nar/gkr1089. PubMed PMID: 22135296.

14. Hogenkamp DG, Arakane Y, Kramer KJ, Muthukrishnan S, Beeman RW. Characterization and expression of the beta-N-acetylhexosaminidase gene family of Tribolium castaneum. Insect Biochem Mol Biol. 2008;38(4):478-89. doi: 10.1016/j.ibmb.2007.08.002. PubMed PMID: 18342252.

15. Arakane Y, Dixit R, Begum K, Park Y, Specht CA, Merzendorfer H, et al. Analysis of functions of the chitin deacetylase gene family in Tribolium castaneum. Insect Biochem Mol Biol. 2009;39(5-6):355-65. doi: 10.1016/j.ibmb.2009.02.002. PubMed PMID: 19268706.

16. Jochim RC, Teixeira CR, Laughinghouse A, Mu J, Oliveira F, Gomes RB, et al. The midgut transcriptome of Lutzomyia longipalpis: comparative analysis of cDNA libraries from sugar-fed, blood-fed, post-digested and Leishmania infantum chagasi-infected sand flies. BMC genomics. 2008;9:15. doi: 10.1186/1471-2164-9-15. PubMed PMID: 18194529; PubMed Central PMCID: PMC2249575.

17. Ramalho-Ortigao M, Jochim RC, Anderson JM, Lawyer PG, Pham VM, Kamhawi S, et al. Exploring the midgut transcriptome of Phlebotomus papatasi: comparative analysis of expression profiles of sugar-fed, blood-fed and Leishmania-major-infected sandflies. BMC genomics. 2007;8:300. PubMed PMID: 17760985.

18. Jasrapuria S, Arakane Y, Osman G, Kramer KJ, Beeman RW, Muthukrishnan S. Genes encoding proteins with peritrophin A-type chitin-binding domains in Tribolium castaneum are grouped into three distinct families based on phylogeny, expression and function. Insect Biochem Mol Biol. 2010;40(3):214-27. doi: 10.1016/j.ibmb.2010.01.011. PubMed PMID: 20144715.

19. Larkin A, Marygold SJ, Antonazzo G, Attrill H, Dos Santos G, Garapati PV, et al. FlyBase: updates to the Drosophila melanogaster knowledge base. Nucleic Acids Res. 2021;49(D1):D899-D907. doi: 10.1093/nar/gkaa1026. PubMed PMID: 33219682; PubMed Central PMCID: PMCPMC7779046.

20. Carver T, Harris SR, Berriman M, Parkhill J, McQuillan JA. Artemis: an integrated platform for visualization and analysis of high-throughput sequence-based experimental data. Bioinformatics. 2012;28(4):464-9. Epub 20111222. doi: 10.1093/bioinformatics/btr703. PubMed PMID: 22199388; PubMed Central PMCID: PMCPMC3278759.

21. Letunic I, Khedkar S, Bork P. SMART: recent updates, new developments and status in 2020. Nucleic Acids Res. 2021;49(D1):D458-D60. doi: 10.1093/nar/gkaa937. PubMed PMID: 33104802; PubMed Central PMCID: PMCPMC7778883.

22. Lee E, Harris N, Gibson M, Chetty R, Lewis S. Apollo: a community resource for genome annotation editing. Bioinformatics. 2009;25(14):1836-7. Epub 20090513. doi: 10.1093/bioinformatics/btp314. PubMed PMID: 19439563; PubMed Central PMCID: PMCPMC2705230.

23. Finn RD, Clements J, Eddy SR. HMMER web server: interactive sequence similarity searching. Nucleic Acids Res. 2011;39(Web Server issue):W29-37. Epub 20110518. doi: 10.1093/nar/gkr367. PubMed PMID: 21593126; PubMed Central PMCID: PMCPMC3125773.

24. Stanke M, Waack S. Gene prediction with a hidden Markov model and a new intron submodel. Bioinformatics. 2003;19 Suppl 2:ii215-25. PubMed PMID: 14534192.

25. Salamov AA, Solovyev VV. Ab initio gene finding in Drosophila genomic DNA. Genome Res. 2000;10(4):516-22. PubMed PMID: 10779491; PubMed Central PMCID: PMC310882.

26. Meireles-Filho AC, Amoretty PR, Souza NA, Kyriacou CP, Peixoto AA. Rhythmic expression of the cycle gene in a hematophagous insect vector. BMC molecular biology. 2006;7:38. doi: 10.1186/1471-2199-7-38. PubMed PMID: 17069657; PubMed Central PMCID: PMC1636064.

27. Waterhouse AM, Procter JB, Martin DM, Clamp M, Barton GJ. Jalview Version 2--a multiple sequence alignment editor and analysis workbench. Bioinformatics. 2009;25(9):1189-91. doi: 10.1093/bioinformatics/btp033. PubMed PMID: 19151095; PubMed Central PMCID: PMC2672624.

28. Katoh K, Asimenos G, Toh H. Multiple alignment of DNA sequences with MAFFT. Methods Mol Biol. 2009;537:39-64. doi: 10.1007/978-1-59745-251-9_3. PubMed PMID: 19378139.

29. Darriba D, Taboada GL, Doallo R, Posada D. ProtTest 3: fast selection of best-fit models of protein evolution. Bioinformatics. 2011;27(8):1164-5. doi: 10.1093/bioinformatics/btr088. PubMed PMID: 21335321.

30. Tamura K, Stecher G, Peterson D, Filipski A, Kumar S. MEGA6: Molecular Evolutionary Genetics Analysis version 6.0. Mol Biol Evol. 2013;30(12):2725-9. doi: 10.1093/molbev/mst197. PubMed PMID: 24132122; PubMed Central PMCID: PMC3840312.

31. Bernsel A, Viklund H, Hennerdal A, Elofsson A. TOPCONS: consensus prediction of membrane protein topology. Nucleic Acids Res. 2009;37(Web Server issue):W465-8. doi: 10.1093/nar/gkp363. PubMed PMID: 19429891; PubMed Central PMCID: PMC2703981.

32. Finn RD, Bateman A, Clements J, Coggill P, Eberhardt RY, Eddy SR, et al. Pfam: the protein families database. Nucleic Acids Res. 2014;42(Database issue):D222-30. doi: 10.1093/nar/gkt1223. PubMed PMID: 24288371; PubMed Central PMCID: PMC3965110.

33. Peng G, Shi X, Kadowaki T. Evolution of TRP channels inferred by their classification in diverse animal species. Mol Phylogenet Evol. 2014;84C:145-57. doi: 10.1016/j.ympev.2014.06.016. PubMed PMID: 24981559.

34. Zelle KM, Lu B, Pyfrom SC, Ben-Shahar Y. The genetic architecture of degenerin/epithelial sodium channels in Drosophila. G3. 2013;3(3):441-50. doi: 10.1534/g3.112.005272. PubMed PMID: 23449991; PubMed Central PMCID: PMC3583452.

35. Friedlander MR, Mackowiak SD, Li N, Chen W, Rajewsky N. miRDeep2 accurately identifies known and hundreds of novel microRNA genes in seven animal clades. Nucleic Acids Res. 2012;40(1):37-52. doi: 10.1093/nar/gkr688. PubMed PMID: 21911355; PubMed Central PMCID: PMC3245920.

36. Langmead B, Trapnell C, Pop M, Salzberg SL. Ultrafast and memory-efficient alignment of short DNA sequences to the human genome. Genome Biol. 2009;10(3):R25. doi: 10.1186/gb-2009-10-3-r25. PubMed PMID: 19261174; PubMed Central PMCID: PMC2690996.

37. Vodovar N, Bronkhorst AW, van Cleef KW, Miesen P, Blanc H, van Rij RP, et al. Arbovirus-derived piRNAs exhibit a ping-pong signature in mosquito cells. PloS one. 2012;7(1):e30861. doi: 10.1371/journal.pone.0030861. PubMed PMID: 22292064; PubMed Central PMCID: PMC3265520.

38. Nowling RJ, Abrudan JL, Shoue DA, Abdul-Wahid B, Wadsworth M, Stayback G, et al. Identification of novel arthropod vector G protein-coupled receptors. Parasites & vectors. 2013;6:150. doi: 10.1186/1756-3305-6-150. PubMed PMID: 23705687; PubMed Central PMCID: PMC3680159.

39. Corpet F. Multiple sequence alignment with hierarchical clustering. Nucleic Acids Res. 1988;16(22):10881-90. Epub 1988/11/25. PubMed PMID: 2849754; PubMed Central PMCID: PMCPMC338945.

40. Sievers F, Higgins DG. Clustal Omega for making accurate alignments of many protein sequences. Protein Sci. 2018;27(1):135-45. Epub 2017/09/09. doi: 10.1002/pro.3290. PubMed PMID: 28884485; PubMed Central PMCID: PMCPMC5734385.

41. Dermauw W, Van Leeuwen T, Feyereisen R. Diversity and evolution of the P450 family in arthropods. Insect Biochem Mol Biol. 2020;127:103490. Epub 20201022. doi: 10.1016/j.ibmb.2020.103490. PubMed PMID: 33169702.

42. Kim D, Paggi JM, Park C, Bennett C, Salzberg SL. Graph-based genome alignment and genotyping with HISAT2 and HISAT-genotype. Nat Biotechnol. 2019;37(8):907-15. Epub 20190802. doi: 10.1038/s41587-019-0201-4. PubMed PMID: 31375807; PubMed Central PMCID: PMCPMC7605509.

43. Wang L, Wang S, Li W. RSeQC: quality control of RNA-seq experiments. Bioinformatics. 2012;28(16):2184-5. Epub 20120627. doi: 10.1093/bioinformatics/bts356. PubMed PMID: 22743226.

44. Katoh K, Standley DM. MAFFT multiple sequence alignment software version 7: improvements in performance and usability. Mol Biol Evol. 2013;30(4):772-80. Epub 20130116. doi: 10.1093/molbev/mst010. PubMed PMID: 23329690; PubMed Central PMCID: PMCPMC3603318.

45. Capella-Gutierrez S, Silla-Martinez JM, Gabaldon T. trimAl: a tool for automated alignment trimming in large-scale phylogenetic analyses. Bioinformatics. 2009;25(15):1972-3. doi: 10.1093/bioinformatics/btp348. PubMed PMID: 19505945; PubMed Central PMCID: PMC2712344.

46. Nguyen LT, Schmidt HA, von Haeseler A, Minh BQ. IQ-TREE: a fast and effective stochastic algorithm for estimating maximum-likelihood phylogenies. Mol Biol Evol. 2015;32(1):268-74. Epub 20141103. doi: 10.1093/molbev/msu300. PubMed PMID: 25371430; PubMed Central PMCID: PMCPMC4271533.

47. Hoang DT, Chernomor O, von Haeseler A, Minh BQ, Vinh LS. UFBoot2: Improving the Ultrafast Bootstrap Approximation. Mol Biol Evol. 2018;35(2):518-22. doi: 10.1093/molbev/msx281. PubMed PMID: 29077904; PubMed Central PMCID: PMCPMC5850222.

48. Kalyaanamoorthy S, Minh BQ, Wong TKF, von Haeseler A, Jermiin LS. ModelFinder: fast model selection for accurate phylogenetic estimates. Nat Methods. 2017;14(6):587-9. Epub 20170508. doi: 10.1038/nmeth.4285. PubMed PMID: 28481363; PubMed Central PMCID: PMCPMC5453245.

49. Subramanian B, Gao S, Lercher MJ, Hu S, Chen WH. Evolview v3: a webserver for visualization, annotation, and management of phylogenetic trees. Nucleic Acids Res. 2019;47(W1):W270-W5. doi: 10.1093/nar/gkz357. PubMed PMID: 31114888; PubMed Central PMCID: PMCPMC6602473.

50. Abdeladhim M, Kamhawi S, Valenzuela JG. What's behind a sand fly bite? The profound effect of sand fly saliva on host hemostasis, inflammation and immunity. Infect Genet Evol. 2014;28:691-703. doi: 10.1016/j.meegid.2014.07.028. PubMed PMID: 25117872.

51. Willis JH, Iconomidou VA, Smith RF, Hamadrakas SJ. Cuticular proteins. In: Gilbert LI, Iatrou K, Gill S, editors. Compgrehensive Insect Science. 4. Oxford: Elsevier; 2005. p. 79-109.

52. McGinnis S, Madden TL. BLAST: at the core of a powerful and diverse set of sequence analysis tools. Nucleic acids research. 2004;32:W20-W5. doi: 10.1093/nar/gkh435. PubMed PMID: WOS:000222273100004.

53. Ioannidou ZS, Theodoropoulou MC, Papandreou NC, Willis JH, Hamodrakas SJ. CutProtFam-Pred: detection and classification of putative structural cuticular proteins from sequence alone, based on profile hidden Markov models. Insect biochemistry and molecular biology. 2014;52:51-9. Epub 2014/07/01. doi: 10.1016/j.ibmb.2014.06.004. PubMed PMID: 24978609; PubMed Central PMCID: PMCPMC4143468.

54. Benoit JB, Hansen IA, Szuter EM, Drake LL, Burnett DL, Attardo GM. Emerging roles of aquaporins in relation to the physiology of blood-feeding arthropods. Journal of comparative physiology B, Biochemical, systemic, and environmental physiology. 2014;184(7):811-25. doi: 10.1007/s00360-014-0836-x. PubMed PMID: 24942313.

55. Li H, Durbin R. Fast and accurate long-read alignment with Burrows-Wheeler transform. Bioinformatics. 2010;26(5):589-95. Epub 2010/01/19. doi: 10.1093/bioinformatics/btp698. PubMed PMID: 20080505; PubMed Central PMCID: PMCPMC2828108.

56. Milne I, Stephen G, Bayer M, Cock PJ, Pritchard L, Cardle L, et al. Using Tablet for visual exploration of second-generation sequencing data. Briefings in bioinformatics. 2013;14(2):193-202. Epub 2012/03/27. doi: 10.1093/bib/bbs012. PubMed PMID: 22445902.
