# Supplementary material for: Genomic analysis of two phlebotomine sand fly vectors of Leishmania from the New and Old World
Source: PLoS Negl Trop Dis. 2023 Apr 12;17(4):e0010862. doi: 10.1371/journal.pntd.0010862 (PMC10138862; doi:10.1371/journal.pntd.0010862)
Supplement: S1 Results — (DOCX) [file pntd.0010862.s002.docx]

**Genomic Analysis of Two Phlebotomine Sand Fly Vectors of *Leishmania* from the New and Old World**

**Supplemental Results: Manual Annotation**

Transposable Elements

Transposable elements (TEs) are coding and non-coding sequences which have a mechanism allowing them to jump within a genome. They are undoubtedly important to analyze and categorize, because they usually make up a significant part of a genome and because of their mobility they thought to be one of the driving forces of the evolution. Genome sizes differ greatly among insect genomes, sometimes even in closely related species. The genome size of *Phlebotomus papatasi* (347 Mb) is approximately twice the size of *Lutzomyia longipalpis* (154 Mb). We found that *Ph. papatasi* genome is composed of 5.65% of TE derived sequences while the *Lu. longipalpis* genome contains only 0.57%. As might be expected due to differences in the genome size, all TE classes and orders were expanded in the *Ph. papatasi* genome comparted to *Lu. longipalpis*. The amount of TE sequence is likely underestimated, due to the nature of the short-read assembly process producing many contigs, sometimes shorter than the length of full-length TEs. Analysis of scaffolds did not improve TE yield, most likely due to the gaps in the assembly. Sequenced BACs were also screened for the presence of TEs and were useful in identification of several full-length retrotransposons.

*Long-terminal repeat retrotransposons (LTR)*

We have identified canonical elements belonging to two of the main superfamilies of LTR

retrotransposons in both the *Ph. papatasi* and *Lu. longipalpis* genomes: gypsy, and pao/bel; and

elements corresponding to the Copia and DIRS superfamilies only in *Ph. papatasi*.

The gypsy group is the most diverse, containing elements belonging to five of the previously

characterized lineages (gypsy, Mag, CsRn1, Osvaldo-like, Mdg1 and Mdg3) and a

phylogenetically new lineage represented by sequences obtained from both genomes. These

sequences grouped together with 100% bootstrap value and they constitute full-length elements, containing all the functional domains (RT, RH, RVE). The CsRn1 lineage was not identified in either sand fly genome.

The Bel-Pao superfamily has been previously classified into seven lineages (Pao, Sinbad, Bel, Tas, Suzu, Flow and Dan) which tend to cluster with the host species phylogeny [1, 2]. Most of the sequences belonging to this superfamily in both sand fly genomes correspond to the Bel lineage while a minority (eight sequences from the *Ph. papatasi* and six from the *Lu. longipalpis*) correspond to the Pao lineage. None of the other lineages within this superfamily are present in these genomes. One sequence from the *Ph. papatasi* genome clustered with the *copia* reference sequences and five with the DIRS references. Neither *copia* nor DIRS elements were identified in the *Lu. longipalpis* genome.

The Gypsy and Bell groups are represented mostly by the same families in both genomes. For instance, the same superfamilies of gypsy are present or absent in both genomes and the “novel” lineage also has representative sequences of both. For the pao/bel group the situation is similar, only elements belonging to the Pao and Bel families are present (the Pao constituting a clade with 88% bootstrap value).

*Non-LTR*

Non-LTR retrotransposon *in-silico* screening identified that the *Ph. papatasi* genome possess 0.92% non-LTRs, whereas the *Lu. longipalpis* genome only contains 0.22%, which is considerably lower than that of *Drosophila melanogaster* (approximately 6%) [3]. In sand flies, the clades which contributed most to the genome size are L2, RTE, Jockey, I and CR1. *Ph. papatasi* also has more variation of non-LTRs than *Lu. longipalpis*. The I clade is the only clade more abundant in *Lu. longipalpis* (0.05%) than in *Ph. papatasi* (0.04%). Members of clades R2 and R4, as well as L1 are not detectable in these genomes. Overall, due to the large size of a full-length non-LTR (5-9 Kb), and comparatively short length of assembled contigs the amount of non-LTRs are likely underrepresented in this study.

*Class II (DNA) TEs*

Annotated DNA transposon content in both sand fly species is lower than that of *D. melanogaster* (*Ph. papatasi* 1.1% of the sequenced genome, *Lu.* *longipalpis* 0.05%, and *D. melanogaster* 2.0% [3]).  It is unclear how much of these differences can be attributed to the quality of the genome assemblies, particularly for elements with such low overall abundances.  Interestingly, P-elements, which are the most abundant DNA transposons in *D. melanogaster* appear to be missing from both sequenced sand fly genomes.  Given that P-elements are a recent invasion in *D. melanogaster,* but have been found in other Diptera, it is not clear whether these sand flies have some defense against P-elements, or the lack of invasion is due to random chance. However, the lack of P-elements in sand flies opens up their possible use for future control strategies. Other DNA transposons, such as the Tc1/mariner elements are found both in *Drosophila* and the sand fly genomes. Information about each Transposable Element’s distribution can be found in Table 1.

*Miniature Inverted-repeat Transposable Elements (MITEs)*

MITEs are short, non-autonomous and do not code for any protein. They have the terminal inverted repeats (TIRs) and target site duplication (TSD) on each flank. These features are the signature used for *in silico* searches of MITEs. Biologically, ITRs are used for the excision of the MITEs from the genome by the corresponding autonomous element’s active transposase, and insertion in a new location. There are 39 and seven annotated MITEs *Ph. papatasi* and *Lu.* *longipalpis*, respectively.  Annotated MITEs also appear to occupy a significantly higher percentage of the genome in *Ph. papatasi* than *Lu.* *longipalpis*.  MITEs that are flanked by TA target site duplications appear to be the most abundant and occupies >2% of the *Ph. papatasi*.  It is not clear how these comparisons may be influenced by the quality of genome assembly.

Immunity

In spite of the potential importance of the sand fly immune system for influencing *Leishmania* development and survival in the sand fly gut the immune response is poorly studied. There have been a number of studies on immune peptides, although to date, this work has been restricted to defensins [4, 5]. It was shown that manipulation of the immune system could lead to inhibition of *Leishmania* development in the gut. Gene depletion via RNAi of negative regulator of IMD pathway caspar [6] led to a reduction in *Leishmania* population in the gut of *Lu. longipalpis*.

*Toll Signaling*

The *Drosophila* Toll signaling pathway is essential in the defense response against Gram-positive bacteria and fungi [7]. Sensing of these pathogens is achieved at the cell surface by recognition of conserved pathogen associated molecules in Gram positive bacteria and fungi. Two pattern recognition receptor (PRR) gene families play a role in this, namely, Peptidoglycan Recognition Proteins (PGRPs) and Glucan Binding Proteins (GNBPs). Recognition results in the activation of proteolytic cascades followed by activation of the Toll signaling pathway. Here we focused on the core signaling molecules in the Toll pathway and on the upstream pathogen recognition receptors including PGRPs and GNBPs.

In both sand fly species’ genomes, Toll signaling pathway is highly conserved as homologues of all the core genes can be manually annotated confidently (S4 Table). For the upstream pattern recognition receptors, PGRPs, *Lu. longipalpis* has four homologues and *Ph. papatasi* has two homologues. All the sand fly homologues share a conserved peptidoglycan binding domain as in the *Drosophila* PGRPs. In *Lu. longipalpis*, two homologues LLOJ002444 and LLOJ005642 have the conserved residues for the enzymatic activity detected in *Drosophila* *PGRP-SA* and *–SD* [8]. These two homologues also show higher identity with *PGRP-SA* compared to *PGRP-SD*. In *Ph. papatasi*, the residues essential for the PGRP-SA and –SD catalytic function is conserved in PPAI010204 only but not in PPAI007689. PPAI010204 also shares higher homology with *PGRP-SA*.

For GNBPs, *Lu. longipalpis* has one GNBP homologue with higher homologue to *Drosophila* GNBP3 and *Ph. papatasi* has four GNBP homologues. Out of the four GNBP homologues in *Ph. papatasi*, two are highly likely to be paralogues. In *Ph. papatasi*, PPAI000880 shows the highest homology with *Drosophila* *GNBP1*, while PPAI010440 exhibits highest homology with *Drosophila* *GNBP3*. Both *Lu. longipalpis* and *Ph. papatasi* have five Toll homologues. All the sand fly Toll receptors have the TIR domain present.

Only one homologue of each of the core signaling molecule downstream of the PRRs, *MyD88*, *tube* and *Pelle*, can be found in both *Lu. longipalpis* and *Ph. papatasi*, with a death domain present in all homologues.

Two homologues of Cactus are present in the *Lu. longipalpis* genome with *Ph. papatasi* only having one. Similarly, *Lu. longipalpis* has two *Dorsal/Dif* paralogues and *Ph. papatasi* has one homologue. The characteristic Rel domain of NF-κB protein family is also found in the sand fly homologues. For the NF-κB inhibitor *Cactus*, the Ankyrin repeat is present.

*IMD/ROS genes*

The Insect Immune Deficiency Pathway (IMD) has an essential role in insect defense against gram-negative bacteria in *Drosophila*. [9-11]. The activation of the IMD pathway is based upon recognition of molecules derived from the bacterial wall through specific receptors (termed peptidoglycan recognition proteins - PGRPs) located on the surface of immunocompetent cells. After receptor activation, a signaling cascade is triggered in the cell cytoplasm culminating with the activation of NF-κB transcription factors such as relish, resulting in expression of antimicrobial peptides. The IMD pathway seems to be highly conserved among dipterans, including *Drosophila*, *Aedes*, *Anopheles* and both sand fly species analysed in this study (S5 Table). Homologues of several genes performing different roles in this pathway were identified for *Lu. longipalpis* and *Ph. papatasi* (S5 Table). Negative regulators Caspar, Caudal and Pirk were found for both sand fly species and Caspar was identified for *Ph. papatasi* for the first time. Interestingly, Caspar knockdown down-regulates *Leishmania* growth within the gut of *Lu. longipalpis* infected with *Leishmania mexicana* and *Leishmania infantum* [6]. Core signalling genes such as IMD, IRD5, IAP2, Effete, Bendless, CYLD; the transcription factor Relish and the Relish activator Dredd are present for both species. TAB1 that forms a complex with TAK1 during the IMD cascade was only found in *Lu. longipalpis*.

The IMD pathway is also connected with other immune-related pathways such as the one responsible for the formation of reactive oxygen species [12]. The production of reactive oxygen species (ROS) such as hydrogen peroxide (H_2_O_2_) and hypochlorous acid occur through the activation of the enzyme Dual oxidase (DUOX) [12, 13]. Calcium ions activate DUOX molecules to produce H_2_O_2_. In the presence of chloride ions, DUOX transforms hydrogen peroxide in hypochlorite which is extremely active against microorganisms. The ROS pathway is a conserved mechanism to control the insect gut flora and protect against pathogenic microorganisms. DUOX is present in the genome of *Lu. longipalpis* and *Ph. papatasi*, DUOX is upregulated through p38-mediated activation of ATF2. p38 is also present in both sand fly genomes (S5 Table). Down-regulation of this pathway involves sequential induction of calcineurin B (CanB) and MKP3, inhibiting p38 activation and reducing *DUOX* transcription [14].

*Galactose-binding proteins*

Galactose-binding proteins (galectins) are a diverse family of proteins playing roles in development and immunity [15]. Comparing the sand flies’ galectin protein sequences with other Diptera, clusters encompassing protein encoded by shared as well as independent orthologs were noticed (S2 Fig.; S7 Table). For instance, the *Leishmania major* receptor in the *Ph. papatasi* midgut PpGalec [16] (here named *PpGalecA*) shares similarities only with its *Lu. longipalpis* ortholog-encoded protein, though the latter bears an extra galactose-binding domain (S2 Fig.; S7 Table). Similarly, other sand fly specific galectins were identified, such as the *Lu. longipalpis* *LuloGalec* [17] (here named *LuloGalecR*), its putative ortholog *LuloGalecR PpGalecR, and LuloGalecII*. On the other hand, the sand fly *GalecB, GalecC, GalecD*, and *Galec_peroxin23* galectins exhibited orthologs in mosquitoes and/or flies (S2 Fig.; S7 Table). Even within a shared protein cluster, a recent gene duplication event was noticed by the presence of two PpGalecB genes in different scaffolds of the *Ph. papatasi* genome (S2 Fig.; S7 Table). Such galectin diversity may be an adaptation of the sand fly immune system to cope with specific symbiont and pathogenic microorganisms.

*TGF-beta*

Transforming growth factor beta (TGF-beta) belongs to a family of multifunctional cytokines found in organisms that go from arthropods and mammals, which regulates functions as diverse as cell differentiation and growth, adhesion, migration, and immune responses [18]. Nevertheless, the TGF-beta pathway components are quite conserved across evolution [19]. TGF-beta is synthesized as a precursor molecule containing a hydrophobic signal peptide, a poorly conserved N-terminal pro-peptide and a highly conserved and active C-terminal domain. The cytokine is secreted as a mature homo or heterodimer [18]. This cytokine superfamily comprises more than forty members, grouped into the following subfamilies according to structure or function: TGF-beta sensu stricto, BMPs (“bone morphogenic proteins”), MIF (“Mullerian inhibitory factor”) and activin/inhibin [20]. Activin/inibin is a polymeric peptide involved in many processes in *Drosophila* as growth, development and neural functions [21]. The TGF-beta family is highly conserved among arthropods and is directly involved in immunity. In the malaria vector *Aopheles stephensi*, a TGF-beta homolog named (As60A) was implicated in the insect immune response to *Plasmodium* [22]. It was also determined that the mammalian TGF-beta 1 ingested in the blood regulates nitric oxide production, modulating the *An. stephensi* immune response against the parasite [23]. The up-regulation of a TGF-beta gene (activin/inhibin subfamily) has been observed in *Lu. longipalpis* upon infection with *Le. infantum chagasi* [24].

We identified fourteen genes in *Lu. longipalpis* genome and fourteen genes in *Ph. papatasi* genome related to TGF-beta or TGF-beta pathways (S8 Table). We found one copy of each gene in *Lu. longipalpis* or *Ph. papatasi* genomes. Additionally, we searched for ortholog genes in *Glossina morsitans*, *Anopheles gambiae* and *Aedes aegypti* genomes and we also found one copy of each gene for these species with exception of *maverick* that was not found in *Ae. aegypti* and *smad on X* that was not found in *An. gambiae*. Among these genes we found two molecules from the TGF-beta superfamily that contain TGF-beta conserved domains, two TGF-beta transcription factors that contain a SMAD (or MAD, for mothers against decapentaplegic) domains, four TGF-beta receptors that contain TGF-beta receptor conserved domains, 1 Dpp (or BMP) ligand that contains a metallopeptidase, a CUB (for complement C1r/C1s, Uegf, Bmp1) and a EGF (for epidermal growth factor) domain, 2 Dpp negative regulators that contain SMAD domains, another negative regulator that contains a WD-40 (for motifs that have approximately forty amino acids often terminating in a Trp-Asp dipeptide) and a F-box (for a motif firstly described in cyclin-F) domain, 1 Dpp inhibitor that contains a magnesium transporter domain, and a Dpp antagonist that contains a CHDN (for chordin) domain and a VWF (for von Willebrand factor) domain.

*MAP Kinases*

Mitogen-activated protein kinases (MAPK) are key components of a series of vital signal transduction pathways that regulate processes such as growth, metabolism, apoptosis, and innate immune responses. The MAPK pathways are a cascade of four regulatory serine-threonine protein kinases which results in the activation of a MAPK that that can regulate effector proteins and transcription factor. MAPKs are phosphorylated and activated by the MAP2Ks (MAPK kinases), also known as MEKs. The MAP2Ks are phosphorylated and activated by MAP3Ks (MAPK kinase kinases), also known as MEKKs [25]. The whole pathway activation starts by the phosphorylation of a MAP4K which is controlled by different cell stimulus. The main MAPKs pathways are the ERK pathway (extracellular signal-regulated kinase), JNK/SAPK pathway (c-Jun NH2-terminal kinase/stress-activated protein kinase) and p38 pathway [26].

In mosquitoes infected with the malaria parasite there is an increase in the production of nitric oxide, which inhibits the proliferation of *Plasmodium* inside the insect. This reaction is caused by the activation of a MAPK signaling pathway [27]. It is known that the activation of the MAPK pathway leading to the NOS production in mosquitoes infected with *Plasmodium* is regulated by the human TGF-beta ingested with the blood [28].

Sixteen different MAPK gene loci were identified in the genome of *Lu. longipalpis* and 15 in the *Ph. papatasi* genome (S9 Table). A *Lu. longipalpis* homologue of *MAP2K3* (ABR28347.1) was found in the transcriptome, but its codifying gene was not in the genome. The number of *Lu. longipalpis* MAPKs is equal to *An. gambiae* [29]. The difference between the two species is that the mosquito has two JNK paralogues and one ERK copy while the sand fly has one JNK and two ERKs. Sand flies also have NLK (Nemo-like kinase) but we only focused on the main MAPKs pathways.

*Other immune-related genes*

Phenoloxidases are ubiquitous type 3 copper-containing enzymes also involved in insect cellular and humoral immune defences. The activated enzyme oxidises phenolic compounds to produce melanin and encapsulate invading pathogens [30]. Due to its high importance and high degree of conservancy among organisms, two prophenoloxidase homologs were found in *Lu. longipalpis* and *Ph. papatasi* genomes (S10 Table).

A TEP-1-like molecule was also found in the genome of *Lu. longipalpis* and *Ph. papatasi* (S4 Table). Tep-1 is a complement-like peptide previously described in *An. gambiae* that interacts and is cleaved by the LRIM-APLC1 complex, targeting *Plasmodium* for destruction [31]. The presence of orthologs of a complement-like system in the sand fly haemolymph raises the possibility that such machinery might be part of sand fly innate immune defences against pathogens.

Eicosanoids act in several lines of defence in insects, inducing phagocytosis, aggregation and haemocyte migration towards an invading pathogen [32]. Cyclooxygenases (COX) are not found in insects, being involved in one of the pathways leading to eicosanoid biosynthesis. Instead, a COX-like molecule was identified in the *Drosophila* genome, possibly exerting a similar role [33]. A COX-like ortholog is present in both sand fly genomes (S4 Table), possibly being involved in pathogen killing through eicosanoid production.

Salivary Protein Genes

Proteomics and transcriptomics have identified the most abundant sand fly salivary proteins from *Ph. papatasi* and *Lu. longipalpis* sand flies. There are 49 *Ph. papatasi* and 35 *Lu. longipalpis* putative salivary genes deposited at the NCBI [34]. We mapped these sequences to the sand fly assemblies (S11 Table).

During blood feeding, female sand flies inoculate saliva into the host dermis where salivary components disrupt hemostasis and the immune system of the host to maintain blood flow. Salivary proteins counteract the coagulation system by blocking clotting and inducing local vasodilation. Additionally, distinct sand fly salivary proteins have immunomodulatory properties reducing inflammation and modulating cellular recruitment to the bite site [34]. Counter-intuitively, repeated exposure to sand fly bites or inoculation of distinct salivary proteins induces an adaptive immune response that can be measured as a delayed-type hypersensitivity response and a specific anti-sand fly saliva antibody response. The antagonistic interaction of some of the sand fly salivary proteins with the host coagulation and immune systems exerts an evolutionary pressure. We can appreciate this phenomenon in the frequency of gene duplication events and the expansion of several sand fly salivary families resulting in the presence of several homologues for some genes. These patterns are highlighted by the presence of motifs assigning conserved families of sand fly salivary proteins that otherwise share a low degree of identity between members when comparing *Ph. papatasi* and *Lu. longipalpis*. This observation suggests a fast evolution pace for salivary genes for example when compared to digestive or olfactory molecules. Below we summarize the function and features of the salivary proteins that were found in the search of the *Ph. papatasi* and *Lu. longipalpis* databases.

*Yellow related proteins*

Yellow related proteins are abundant molecules in both species. Despite their size, around 40-kDa, they bind and trap biogenic amines such as serotonin, histamine and others in a central pocket. Removal of biogenic amines from the environment inhibits the activation of the coagulation system or reduces the availability of inflammatory mediators. LJM111, a yellow protein from *Lu. longipalpis*, has anti-inflammatory properties inhibiting IL-17, TNF-α, IFN-γ and neutrophil migration in an ovalbumin-induced neutrophil migration mouse model [35]. LJM11 and LJM17, other yellow proteins from *Lu. longipalpis* are immunogenic and immunization with LJM11 protects mice from *Leishmania* infection [36]. Conversely, immunization with PpSP44, the most abundant yellow protein from *Ph. papatasi*, led to exacerbation of parasite infection [37]. No function has been assigned to yellow proteins from the *Ph. papatasi* sand fly.

*D7 related Proteins*

PPTSP30 and LJL13 D7 belong to the D7 related proteins and have been found in New and Old World species of sand flies. They are classified as part of the odorant binding family of proteins [38]. They are also present in several other blood feeding insects such as mosquitoes and black flies. The function of these proteins remains unknown in sand flies, but it is possible that they also work by binding biogenic amines and eicosanoids similarly to mosquito D7 proteins [39].

*Silk related/collagen binding/32kDa family of proteins*

These salivary proteins are commonly found in saliva of most studied sand fly species. PpSP32 from *Ph. papatasi* has been characterized as the main marker of exposure to sand fly bites in an endemic area of cutaneous leishmaniasis in Tunisia [40]. There have been no functional studies undertaken on the *Lu. longipalpis* salivary protein LJL13.

*Antigen 5 family of proteins*

PPTSP29 and LJL34 belong to the Antigen 5 family of proteins. These proteins are commonly present in many bloodsucking insects and are associated to the CAP family of proteins composed of cysteine-rich secretory proteins, Antigen 5, and pathogenesis-related 1 proteins [41].

*Apyrases*

Apyrases are enzymes that hydrolyze ATP and ADP preventing platelet aggregation and facilitating blood feeding. Apyrases are present in saliva of all sand fly species studied; LJL23 and PPTSP36 represent the Apyrases for *Lu. longipalpis* and *Ph. papatasi* sand flies, respectively [41].

*Maxadilan*

One of the best-characterized molecules in *Lu. longipalpis* saliva, maxadilan, it is a powerful vasodilator and an anti-inflammatory peptide of 6.8 kDa [42]. Vaccination with DNA encoding maxadilan protected mice against *Leishmania* infection [43].

*Anti-coagulant*

LJL143 from *Lu. longipalpis*, named Lufaxin, is an inhibitor of Factor Xa, disrupting both the intrinsic and extrinsic coagulation cascades. Moreover, by its interaction with the PAR receptors it can modulate inflammation [44].

*Endonucleases*

LJL138 from *Lu. longipalpis*, named Lundep, is an endonuclease that cleaves DNA and, at a lower efficiency, RNA. Lundep has a role in blood feeding through inhibition of the intrinsic pathway of coagulation. Additionally, Lundep cleaves neutrophil extracellular traps enhancing the survival of *Leishmania* parasites upon deposition into the host tissue [45].

*Hyaluronidases*

Following the discovery of sand fly salivary transcripts coding for members of the hyaluronidase family [46], as well as biochemical detection of the enzyme activity in salivary homogenates [47-49], and the demonstration that co-injection of *Leishmania* parasites with hyaluronidases enhance the protozoan infection [50], the recombinant enzyme of *Lu. longipalpis*, named LuloHya, was produced and shown to be protective against *Leishmania* infections when used as a vaccine [51].

*10-kDa family of proteins*

LJS192, LJS169 and LJM19 are proteins of unknown function that are abundant in the saliva of *Lu. longipalpis*. Proteins of similar molecular weight but with low identity have been identified from the *Le. intermedia* salivary transcriptome [52]. Vaccination with LJM19 protects hamsters from cutaneous [53] and visceral [54] leishmaniasis.

*C-type lectin*

LJL91, LJL15, LJL18, LJM10 and LJS142 from *Lu. longipalpis* are members of the C-type lectin family of proteins that seems to be unique to New World sand flies. No function has been assigned to this family of proteins.

*PpSP56.6*

Previously identified by transcriptomic analysis, the function of PpSP56.6 from *Ph. papatasi* is unknown. There is one other member of this family identified from *Phlebotomus sergenti* saliva [55].

Amylase PPTAMY, a putative alpha-amylase from *Ph. papatasi*, hydrolyzes dietary starch to maltose, which is then cleaved to glucose by alpha glucosidase. Starch if one of the major components in the natural diet of *Ph. papatasi*.

Digestion

Digestive processes in Phlebotomine sand flies are of key interest in terms of the relevance to the gut dwelling *Leishmania* parasite. Successful development and emergence from the digesting blood meal is an essential feature for the development of a transmissible parasite population in the gut. A number of studies suggest that expression of digestive proteases may be influenced by the presence of *Leishmania* [56]. Inhibition of trypsin like protease via gene depletion enhanced *Leishmania* survival in *Lu. longipalpis* [57].

*Peptidases*

Peptidases (E.C. 3.4) are enzymes responsible for the hydrolysis of peptide bonds. They are classified according to the site of cleavage in the substrate, in exopeptidases (terminal bonds) or endopeptidases (internal bonds). Besides that, peptidases are classified by their catalytic mechanism in aspartic, cysteine, glutamic, metallo, asparagine, serine, threonine and mixed type peptidases. A more recent approach is the classification of peptidases in families based on the similarity of amino acid sequences, which reflect the overall structure and the catalytic mechanism of homologous enzymes [58].

We conducted a systematic search in the genomes of *Lu. longipalpis* and *Ph. papatasi* for genes belonging to all known peptidase families, using HMMER/blast and the results are presented in S12 Table. Both genomes contain at least 376 protease genes belonging to several families (64 *Lu. longipalpis* and 62 in *Ph. papatasi*), with a predominance of serine proteinases (48.4 and 46.3% of total protease genes, respectively). This distribution is similar to the observed in other dipterans known genomes, where serine proteases account for 45-64 % of all protease genes. This is consistent with the involvement of serine proteases in larval and adult sand fly digestion. Because larval and adult initial protein digestion rely mainly on the same type of proteinases from family S1, tissue specific gene expression studies are necessary to elucidate if some gene expansion in this family occurred during sand fly adaptation to hematophagy.

Interestingly, two proteinase families were found to be unique to sand flies among dipterans. These are families C95 and M87. Families C95 and M87 are proteinases which are present in other animals [59, 60] and its occurrence in *Acyrtosiphon pisum* suggests that these are ancient sequences which might be present in the hexapodan ancestor. However, its occurrence is sparse throughout the animal kingdom, and more detailed studies are necessary to elucidate their evolutionary origin in sandflies. Notably, family M87 correspond to chloride channel associated proteins, and as the chloride channel is the target of several insecticides, its occurrence in sand flies can be the basis for insecticide selectivity between these vectors and other organisms.

Nevertheless, the number of protease genes in the sand fly genomes seem to be lower than the observed in other dipteran species. This could be a result from under representation arising from assembly or search strategy issues, or just a reflection of different protease gene expansions in culicidae and brachycera. These expansions might have occurred mainly in family S1, which harbor the majority of serine proteinase sequences. However, the number of serine proteinase genes found in sand fly genomes (177 and 174 for *Lu. longipalpis* and *Ph. papatasi*) is similar to the observed in other insect genomes (like *Ac. pisum*, 197 genes, or *Apis mellifera*, 108 genes).

*Carbohydrases*

Carbohydrases, also known as glycoside hydrolases, are essential enzymes involved in sugar metabolism. They hydrolyse glycosidic bonds and are mainly classified by the substrate and glycosidic bond they recognize. Recently, they were categorized by amino acid sequence similarities in families (Glycoside Hydrolase Families, or GHFs), which reflect the overall enzyme structure and catalytic mechanism [61]. In sand fly genomes, the number of GH genes we found using a general approach (HMMER/Blast) is quite low (±30) when compared with other insect genomes (~100). This is probably a result of gene fragmentation and masking of low-quality sequences. Because of that, we focused in two GH families of special interest, GHF13 and GHF18.

*Glycoside Hydrolase Family 13 – Starch, Glycogen and Sucrose metabolism*

In general, members of GHF13 are enzymes active on alpha-glycosidic bonds which are typical of starch or glycogen. Most members of GHF13 are typical amylases and alpha-glucosidases, but GHF13 also contains enzymes with pullulanase, cyclomaltodextrinase or transferase activities, among others. Both *Lu. longipalpis* and *Ph. papatasi* genomes contain at least 15 GHF13 genes, summarized in S13 Table. These numbers were updated in a more recent and depth analysis of GH13 proteins in *Lu. longipalpis* that was published elsewhere [62], with an account of at least 21 proteins. These high copy numbers reflect not only the multi enzymatic nature of this glycosidase family, but also the importance of these enzymes in larval and adult metabolism. These enzymes are involved mainly in starch/glycogen digestion in larvae and sucrose digestion in adults of both sexes. Accordingly, amylase and alpha-glucosidase were already detected and partly characterized in *Lu. longipalpis* [63] and some *Phlebotomus* species [64, 65] .

Sucrose digestion in insects is routinely performed by alpha-glucosidases (E.C. 3.2.1.20), as reports for beta-fructosidase (E.C. 3.2.1.26) are very scarce [66]. As expected, sand fly genomes do not contain genes belonging to GHFs 32, 68 and 100, which are the gene families for which this activity was already reported [61]. Comparison with other insect genomes suggest the presence of genes orthologous to sand fly GHF13 genes in other dipterans genomes, as described in S13 Table. In many cases, several sand fly sequences are more similar to the same ortholog in *D. melanogaster*, *An. gambiae*, *Ae. aegypti* or *Culex quinquefasciatus*, which suggest that GHF13 diversification might have happened after the divergence of dipteran families.

The initially discovered number of GHF13 genes in sand fly genomes (15) is similar to the observed in *D. melanogaster* (16) and *An. gambiae* (17), higher than the number in other vectors as *Rhodinus prolixus* (6) and *G. morsitans* (8), but lower than the numbers found in *Ae. aegypti* (27) and *C. quinquefasciatus* (33). This expansion in GH13 gene family in some insect genomes could be related to the fact that alpha glucosidase is a major membrane protein in the several insect midgut epithelia (especially in the order Diptera), being the target receptor of several bacterial endotoxins [67]. This might be the motor of evolutionary co-adaptations between insects and bacterial pathogens, which could involve gene duplications, mutations and presence of isoforms conferring resistance to the insect against entomopathogenic microorganisms. Interestingly, the infection of *Lu. longipalpis* adult females with *Le.* *mexicana* changes the expression of alpha-glucosidase genes in the gut, but it is still not clear if this is a direct mechanism involving regulation by the pathogen, or of it is a indirect consequence of the general effects of the parasite in the digestion of sugar and blood [62].

*Chitinase and Chitinase-like proteins*

Insect glycoside hydrolases from family 18 (GH18) comprise chitinases and chitinase-like proteins. Insect genomes contain several copies of GH18 genes, as a reflection of the pivotal role of chitin structures in insect anatomy, development and physiology. Chitinases and chitinase-like proteins are categorized into eight groups based on their catalytic activity, domain structure (presence of catalytic domains, linker regions and chitin-binding domains) and amino acid sequence similarities. These groups have distinct physiological roles which were assigned using expression pattern and RNAi silencing studies in *D. melanogaster*, *Tribolium castaneum*, *An. gambiae* and *Ae. aegypti* [68, 69]. They are involved, for example, in molting, digestion, peritrophic matrix turnover, developmental morphogenesis and cell signaling. In the sand fly genomes we have found 11 (*Lu. longipalpis*) and 12 (*Ph. papatasi*) GH18 genes, representing all known chitinase groups and including 1 Imaginal Disc Growth Factors for each specie. Original VectorBase [70-72] gene sequences were completed and corrected using de novo assembly of transcriptome data. Characteristics of curated chitinase gene sequences are summarized in S14 Table. In the case of *Lu. longipalpis* chitinase genes, ten are present in the original assembly and have VectorBase Accession numbers, and 1 were found only in de novo assembly of RNA-Seq data. GH18 gene copy numbers in sand fly genomes are more similar to the observed in *Drosophila* (16 genes, with 6 IDGF copies) than the genomes of *An. gambiae* (20 genes, 2 IDGFs), *Ae. aegypti* (16, 2 IDGFs) or *T. castaneum* (22, 2 IDGFs). GH18 proteins in sand fly genomes contain a variable number of catalytic domains (1-4) and chitin binding domains (0-4), presence or absence of signal peptide or transmembrane regions and highly glycosylated linker regions. The conservation observed in the chitinase gene number and gene structure among the dipteran genomes is probably related to the conservation of chitin role and metabolism in the insect orders. As observed in other insects, the multigenic nature of GH18 family points their members as interesting models for gene expression and physiological studies as well as targets for insecticide molecules.

*N-acetylhexosaminidases*

N-acetylhexosaminidases (HEXs) belong to the glycosyl-hydrolase family 20 [73], and participate in N-glycan processing (fused lobe proteins - FDLs; [74] as well as digestion of carbohydrate oligomers to monomers [75]. The HEX family member exo-splitting β-N-acetylglucosaminidases (NAGs) have been implicated in cuticular chitin turnover during insect molting as well as in peritrophic matrix degradation [75-77]. In sand flies, four orthologs belonging to n-acetylhexosaminidases groups I-IV were identified in each sand fly species (S15 Table; Fig S3). Interestingly, a gene duplication event has taken place only in the sand flies NAG1 gene, giving rise to an extra NAG gene (NAG3), which is shared by both sand fly species (S15 Table; Fig S3), therefore, sand flies bear the necessary machinery to recycle chitin along with an extra gene copy that may perform a more specialized function.

*Chitin deacetylases*

Chitin deacetylase proteins (CDA) belong to the carbohydrate esterase family CE4 and converts chitin into chitosan via N-deacetylation. This reaction might contribute to the binding of some proteins to chitosan in chitinous structures in the cuticle and peritrophic matrix [78]. In the red flour beetle (*T castaneum*), five CDA groups have been described, displaying specific expression in either cuticle or peritrophic matrix [79]. In sand flies, CDAs belonging to the five groups presented in insects were identified (S16 Table; S4 Fig.), suggesting chitin deacetylation may also be an important biological process in the sand fly’s cuticle and PM.

*Peritrophin-like proteins*

Peritrophins are abundant component of insect PM and cuticle, in which they create the structural scaffold by cross-linking chitin fibrils [80]. These proteins bear similar chitin-binding domains (CBDs) to chitinases and can also display mucin-like motifs, which are heavily glycosylated [81]. In sand flies, 30 peritrophin genes were identified in *Ph. papatasi*, whereas 22 homologs were described in *Lu. longipalpis* (S17 Table). As far as domain number and diversity, up to nine CBD domains were found in a single peritrophin (PpPer9), and multiple sand fly peritrophins were predicted to be glycosylated by exhibiting mucin-like motifs (S17 Table). In a comparative analysis between sand flies and *T. castaneum* [82] CBD domains, multiple sand fly and RFB domains belonging to the CPAP subgroup (cuticular proteins analogous to peritrophins) clustered together (S5 Fig.). On the other hand, only one sand fly CBD domain clustered together with the *T. castaneum* CBD domains of the PMP subgroup (peritrophic matrix proteins) associated with PM scaffolding (S5 Fig.). These differences highlight the functional specialization of peritrophin-like proteins playing a role in peritrophic matrix formation as well as a more conserved role for the peritrophins associated with the insect exoskeleton.

Aquaporins

Aquaporins (AQPs) are required for the transportation of water and other small solutes across cell membranes. We have identified six aquaporin genes from both species of sand flies (S18 Table; S6 Fig.). This is similar to the number present in mosquitoes (N = 6), but two and four less than *Drosophila* and *Glossina*, respectively [83]. Members of each AQP group previously identified from insects are present in the sand fly genomes, which includes those involved in the water and the recently assigned to be involved in glycerol transport [84].

Circadian Rhythm Genes

Most organisms present an endogenous mechanism that allows the responsiveness to different environmental stimuli such as light and temperature, the biological clocks. Among them, the circadian clocks (which, in absence of external cues, last about 24 hours), regulate rhythms most closely related to daily behaviours such as locomotion, oviposition and larval eclosion.

In the model species, *D. melanogaster*, the molecular basis of the circadian clock has been well studied for decades. Genes that are known to influence several behaviours or encode for receptors involved in the detection of heat, water or mechanical stimuli are controlled by clock genes and have been characterized, mainly in *D. melanogaster*, *Ap. mellifera,* and *An. gambiae* [85-95].

The major components of the circadian clock form three interlocked feedback loops. In the main loop, the *Clock (Clk)* and *cycle (cyc)* genes encode two activators, CLK and CYC, that form a heterodimer and bind in regulatory sequences called E-boxes (CACGTG) in the promoters of *period (per)* and *timeless* (*tim*) genes, activating their transcription [88]. After a series of post-translational changes, PER and TIM proteins form a dimer, enter in the nucleus and inhibit CLK/CYC function, in a cyclic manner. In the second loop, the genes *vrille* (*vri*) and *PAR domain protein 1* (*Pdp1*), which are also cyclically activated by the CLK/CYC heterodimer, encode a repressor (VRI) and an activator (PDP1), respectively, which in turn compete for the same site in the *Clk* promoter, regulating its transcription. The last described loop involves the regulation of CLK/CYC targets by *clockwork-orange* (*cwo*) gene that encodes a repressor that competes with CLK/CYC for E-box binding to repress the transcription of clock-controlled genes [88].

Insects that are vectors of many pathogens, such as virus, protozoans and worms, for example, have a precise influence of the circadian clock to a successful vectorial capacity [96]. Although hematophagy and host seeking are controlled by the clock, the molecular regulation is poorly understood in sand flies. In *Lu.* *longipalpis*, the expression pattern of the core loop was described and present striking differences when compared to the *Drosophila* pattern, mainly in *cyc* expression. While *Drosophila* presents a constitutive *cyc* expression, *Lu. longipalpis* has a cyclic expression of this gene, with great amplitude [97]. Until now, no description of the molecular clock of *Ph. papatasi* has been available.

All core clocks genes were found in *Lu. longipalpis* (S19 Table) and *Ph. papatasi* (S20 Table) genomes (*period, timeless, cycle, Clock*, photolyases, *clockwork orange*, *vrille*, *Pdp1*), as well as post-translational modifiers (kinases and phosphatases). Other output circadian genes were successfully confirmed in both genomes (*timeout, single minded*, *slimb, tango, nemo*, *cacophony, paralytic, narrow abdomen, slowpoke, nocte, ataxin-2, circadian trip* and *takeout-like*). The amount of takeout/Juvenile Hormone Binding Protein copies found in *Lu. longipalpis* and *Ph. papatasi* was far below than it is found in other dipterans (5-6 in sand flies against 24-30 copies in the other genomes). Surprisingly, it was found two copies of the *casein-kinase-2* gene in *Lu. longipalpis* genome, whereas in *Ph. papatasi* and other dipterans there is only one copy of this gene. Another difference between the sand fly genomes is that serine/threonine phosphatase 2-beta (Pp2-b) is not found in *Lu. longipalpis*, while Pp1-beta and Pp4 are absent in *Ph. papatasi*. The most striking finding is the absence of a cryptochrome-1 in *Lu. longipalpis* genome and transcriptome, and its presence in the species *Ph. papatasi* (S7 Fig.).

Nine and ten members of the transient receptor potential (TRP) cation channel family have been found in *Lu. longipalpis* and *Ph. papatasi* genomes, respectively. The presence of six predicted transmembrane domains and the PF00520 (ion transport protein family) and PF12796 (ankyrin repeats) domains, characteristic features of this protein family, was confirmed in the sequences of all candidates. *D. melanogaster* orthologous sequences of *pyrexia*, *TRPA5* and *TRPP* seem to be absent in both sand fly genomes. No differences in TRPM, TRPML, TRPC and TRPA subfamilies were detected between both sand fly species, except for water which gene (belonging to TRPA subfamily) with five and three paralogs in *Lu. longipalpis* and *Ph. papatasi*, respectively. Interestingly, NompC (belonging to TRPN subfamily) has been identified in *Lu. longipalpis* genome; however, it is absent in *Ph. papatasi*. Regarding TRPV subfamily, *inactive* and *nanchung* ortholog were detected only in *Ph. papatasi*. The TRP phylogenetic tree showed a separation of the different TRP subfamilies [98] and high bootstrap values supporting the different clades (S8 Fig.).

In the case of pickpocket (PPK) family, fourteen and thirteen members have been identified in *Lu. longipalpis* and *Ph. papatasi* genomes, respectively. All candidates presented the two predicted transmembrane domains and the PF00858 domain corresponding to amiloride-sensitive sodium channel superfamily. The phylogenetic tree demonstrated a division of the six different PPK subfamilies, as proposed by Zelle et al. [99] (S9 Fig.). None of the genes represented in *D. melanogaster* PPK subfamily III was present in the sand fly genomes. The orthologue of *ppk28*, which is related to water perception in *D. melanogaster* [100, 101], has been identified in both sand fly species (S9 Fig.). Other *D. melanogaster* *ppk* orthologues (*ppk16*, *ppk31* and *ppk3*) were identified in *Ph. papatasi* and *Lu. longipalpis* (S9 Fig.). In the case of sand fly *ppk100-105*, it was impossible to identify clear fruit fly orthologous sequences. Interestingly, sand fly *ppk100*-103 and *ppk-like* from *Lu. longipalpis* grouped to the conserved subgroup *ppk*, *rpk* and *ppk26* (S9 Fig.). It is important to mention that *Lu. longipalpis* *ppk101*, *ppk102* and *ppk*-like are clustered in the same region of the genome (S19 Table). An identical situation was observed for *Ph. papatasi* *ppk100a* and *ppk100b* and *ppk102* and *ppk103* (S20 Table).

Other behavioural genes identified include: 1) *foraging* (*for*), which has been related to different patterns of locomotor activity in *Drosophila* [91, 102], locusts [103] and honeybees [85, 104]; 2) *malvolio* (*mlv*), which has been associated to labour division in honey bee [105] and normal taste behaviour in *Drosophila* [106]; 3) *stripe* (*sr*), involved in fly orientation in *Ap. mellifera* [107]; and 4) *piezo*, which mediates noxious mechanosensory stimuli for *Drosophila* [86].

Interestingly, both sand fly species presented a number of receptors related to the detection of moist air (Llonwtwr1-5 and Ppapwtrw1-3) [108] greater than that observed in other insects [109]. Besides the Dmelppk28 orthologue, related to water reception [100], has been identified in both sand fly genomes. The presence of these receptors could be related to the susceptibility to dehydration of these insects due to its small size. In fact, a high constant humidity is required for rearing *Lu. longipalpis* and *Ph. papatasi* in laboratory [110]. Regarding TRP superfamily, the main difference in those members (*nanchung, inactive* and *NompC*) is related to hearing [111]. Functional genetics studies would be necessary to assess the meaning of these differences between both species. The number of PPKs identified in both sand fly species (13 and 14) is lower than those reported from *An. gambiae* and *D. melanogaster*, 31 and 18 respectively [99]. Both sand fly genomes encode five different *ppks*, which clustered with the conserved group *ppk1, ppk2* and *ppk26* of subfamily IV. A similar gene expansion has been observed in *An. gambiae*, specifically with *ppk26* and four related subunits [99], however, in case of sand flies its relation to the IV subfamily members was not well resolved in our phylogenetic tree. Besides, at least one *ppk* from one sand fly genome has been identified in all PPK subfamilies, except for subfamily III, which is not shared by all dipteran species [99]. The differences found between *Lu. longipalpis* and *Ph. papatasi* *ppk* members, e.g. Llonppk9, Llonppk13 or Ppapppk23; need further investigation. Regarding other genes identified, e.g. *foraging* or *stripe*, behavioural and functional genetic studies would be necessary to confirm the conservation of these gene functions in sand flies [112].

Cytochrome P450s

CYP6AK and CYP6AG have been considerably expanded in *Lu. longipalpis* and at a lesser extent in *Ph. papatasi*, while CYP9J/9L has been almost equally expanded in both species (S10 Fig.). Further examination suggests that these expansions have been probably caused by tandem gene duplications. In particular, expansions of CYP9J/9L and CYP6AK have each formed two large gene clusters in the *Lu. longipalpis* genome*,* which consist of 15 consecutive CYP9J/9L-like and 10 consecutive CYP6AK-like genes, respectively. The CYP6AG expansion also formed a gene cluster that is conserved in both sand flies and it is composed of seven and five tandem genes in *Lu. longipalpis* and *Ph. papatasi*, respectively.

The CYP3 clan is generally associated with xenobiotic detoxification [113]. Indeed, two of the three expanded subfamilies have been previously implicated in xenobiotic metabolism and insecticide resistance in mosquito vector species. In particular, CYP9J enzymes are involved in xenobiotic detoxification and pyrethroid resistance in the dengue vector, *Ae. aegypti* [114]. *CYP6AK1* has been associated with *A. gambiae* permethrin resistance. Therefore, expansions of these subfamilies in *Lu. longipalpis* and *Ph. papatasi* could possibly reflect sand fly-specific adaptations to environmental challenges posed by xenobiotics.

Sand fly P450 diversity is much more limited in the other three CYP clans. The Mito clan is complete in *Lu. longipalpis* and *Ph. papatasi*, since all the conserved genes are present in both sand flies (S10 Fig.). Importantly, the Mito clan genes implicated in ecdysteroid metabolism are present in both sand flies, even though they are all fragmented in *Ph. papatasi*. Namely, CYP315A1, CYP302A1, and CYP314A1 are present and cluster confidently with their *A. gambiae* orthologs (S10 Fig.).

The CYP2 clan is also complete, with a sand fly ortholog in each of the major clades of this clan (S10 Fig.). There is a total of 13 sand fly CYPs, three of which are full-length. These full-length genes are the orthologs of CYP303A1, CYP304B1, and CYP305A1. More specifically, *Lu. longipalpis* and *Ph. papatasi* have three and four CYP304B1-like genes respectively, most probably indicating species-specific duplications. The ecdysteroid metabolism genes of this clan, CYP306A1 and CYP307A2 are also present. Finally, orthologs of the mosquito CYP15B1, which is implicated in the juvenile hormone biosynthesis, is also detected in both sand fly genomes.

*Lu. longipalpis* and *Ph. papatasi* have contracted CYP4 clans, mostly caused by an *An. gambiae*-specific expansion of the CYP4H subfamily. Orthologs for each member of the conserved CYP4G subfamily which is involved in cuticular hydrocarbon biosynthesis, also exist in both sand fly genomes.

It is worth noting that there are some conserved P450s that appear to be duplicated in *Ph. papatasi*, such as CYP314A1, CYP307A2, CYP15B1 and CYP4G17. However, all these genes are known single-copy genes in insects and they are also single-copy in *Lu. longipalpis*. As a result, the apparent *Ph. papatasi*-specific duplication is most likely due to the fragmentation of the *Ph. papatasi* genome assembly.

RNA genes and MicroRNAs

RNA interference (RNAi) refers to pathways that utilize small non-coding RNAs (ncRNAs) associated with Argonaute proteins to regulate gene expression. Most animals, including insects, have at least three separate classes of small ncRNAs known as microRNAs (miRNAs), small interfering RNAs (siRNAs) and piwi-interacting RNAs (piRNAs). These classes of small RNA differ in their mechanism of biogenesis and action. Specifically, each class of small RNA is associated with different argonaute proteins and, in the case of miRNAs and siRNAs, also ribonuclease III proteins such as Dicer and Drosha and small dsRNA binding protein partners. The miRNA pathway regulates the expression endogenous gene thus being required for development and most biological responses. The siRNA pathway controls the expression of transposable elements in somatic tissues and also mediates a powerful antiviral defense. The piRNA pathway regulates the activity of transposable elements in the animal germline.

The mechanism of RNAi has been extensively characterized utilizing the fruit fly *D. melanogaster* as an animal model. Thus, we have utilized *D. melanogaster* RNAi genes as a reference to help identify and characterize core RNAi genes and small ncRNAs in *Lu. longipalpis*. Utilizing this approach, we have identified genes encoding core genes for all three RNAi pathways in the *Lutzomyia* genome: *Drosha, Pasha, loqs, Dcr-1 and AGO1* (two separate copies) for the miRNA pathway; *R2D2, Dcr-2* and *AGO2* for the siRNA pathway; *AGO3* and *AGO4* Piwi genes for the piRNA pathway (S22 Table). We have also found orthologs for most RNAi genes in the *Ph. papatasi* genome although we could not find genes corresponding to R2D2 and AGO2 and only found 2 copies of piwi genes. These genes are not likely to be absent in the *Ph. papatasi* genome but rather just not included in the current assembly.

In addition, we have deep sequenced and analyzed small RNA libraries from female adults of *Lu. longipalpis* in order to annotated potential ncRNA genes corresponding to miRNA genes and piRNA clusters. We annotated 88 unique and 113 total miRNA precursor genes, including 58 unique genes that are conserved in other insects and 30 unique miRNA genes that seem to be *Lu. longipalpis* specific. Total number of miRNA genes is comparable to what has been observed in *Ae. aegypti* that has 101 annotated miRNA precursors. However, it is likely that we have missed a few miRNA genes as *D. melanogaster* has 256 miRNA precursor genes annotated. We also identified 29 genomic clusters that generate piRNAs in the *Lu. longipalpis* genome based on a pattern search approach first applied to *D. melanogaster*. Although piRNA clusters tend to be repeat rich regions and show low sequence conservation comparing different insects, we have clearly identified conserved characteristics in *Lutzomyia* piRNAs compared to *Drosophila* such as size and a clear ping-pong signature associated with a piRNA-specific amplification mechanism.

In summary, we have found RNAi genes and non-coding RNAs corresponding to all three major RNAi pathways in *Lu. longipalpis* and *Ph. papatasi*. These RNAi pathways show different degrees of conservation compared to *D. melanogaster* and other Dipteran insects, with miRNAs being the most conserved and piRNAs the most divergent.

Heat Shock Protein Genes

Comparison of *D. melanogaster* proteins with GO terms relating to heat shock and response to hypoxia to the hypothetical scaffolds of the two sand fly species *Lu. longipalpis* and *Ph. papatasi* allowed for identification of 83 and 78 orthologs, respectively, in each species (S23 Table). These comparisons were accomplished by BLAST comparison of the known *D. melanogaster* heat shock and hypoxia proteins to the scaffolds and predicted genes in *Lu. longipalpis* and *Ph. papatasi*. Genes that were identified as possible orthologs were then compared by BLAST to the arthropod-specific BLAST database using CLC Genomics Workbench 7. Due to a high degree of similarity, 53 of the *Lu. longipalpis* proteins require no revisions while 14 require minor revisions and 16 require large-scale revisions. For *Ph. papatasi* gene predictions, 44 require no revisions while 13 require minor revisions and 21 require major revisions. Overall, there appears to be no interesting expansions or retractions in gene families associated with heat shock or hypoxia for either sand fly species.

Cuticular Protein Genes

Cuticular protein sequences containing the R&R consensus chitin-binding domain, hence belonging to the Cuticle Protein R&R (CPR) family, were identified from *Lu. longipalpis* and manually annotated using CLC Genomics Workbench 7. Relatively all of the predicted CPR family genes need substantial revision (these are marked with a Y in cuticular (S24 Table).  *Lu. longipalpis*, 74, and *Ph. papatasi,* 84, have far fewer genes encoding CPR proteins than *D.* *melanogaster*, *An.* *gambiae*, and *Ae. aegypti*  (101, 158 and 240, respectively, [115]). An additional 43 sequences belonging to other cuticular protein families were identified for *Lu. longipalpis* and 41 additional sequences for *Ph. papatasi*. All of these putative cuticular proteins were defined based upon sequence similarity through BLAST to proteins that were either isolated directly from cuticle or appeared in genomic analysis of *Drosophila* as cuticle proteins. Although we note a major reduction in the number of cuticle proteins, we are hesitant to confirm this is the case due to the fact that many of the CPR gene predictions were of low quality and need revision, suggesting that a few to many genes might not have been predicted or are predicted incorrectly.

Hormonal Signaling

*Juvenile hormone signaling*

Juvenile hormone signaling gene were identified by BLAST comparison of the known *D. melanogaster* heat shock and hypoxia proteins to the scaffolds and predicted genes in *Lu. longipalpis* and *Ph. papatasi*. Genes that were identified as possible orthologs were then compared by BLAST to the arthropod-specific BLAST database using CLC Genomics Workbench 7. Genes identified as components of the juvenile hormone signaling pathway are highly conserved between *D. melanogaster*, *An. gambiae*, *Ae. aegypti* and the two sand fly species, *Lu. longipalpis* and *Ph. papatasi* (S25 Table). This is fairly unsurprising given the deep evolutionary conservation for the fundamental aspects of juvenile hormone signaling [116]. The methoprene tolerant (met) gene is recognized as the best candidate juvenile hormone receptor. In Brachycera, two paralogous JH receptor candidates Met and gce are thought to transduce the juvenile hormone signaling pathway. The *gce* gene is homologous to “Met” in other Diptera, such as mosquitoes. In the two sand flies, the *gce* gene is more homologous to given gene model rather than the met gene, which is similar to that in *An. gambiae* and *Ae. aegypti*.

*Insulin signaling*

Comparison of *D. melanogaster* proteins with GO terms relating to insulin signaling processes to the scaffolds of the two sand fly species *Lu. longipalpis* and *Ph. papatasi* using BLAST methods described in the insulin signaling section allowed for identification of 31 and 30 orthologs, respectively, in each species associated with insulin signaling (S26 Table). Due to a high degree of similarity, 14 of the *Lu. longipalpis* proteins require no revisions while 5 require minor revisions and 12 require large-scale revisions. 13 of the *Ph. papatasi* gene predictions also require no revisions while 7 require minor revisions and 10 require major revisions. While not all insulins signaling proteins were identified this does not indicate a lack of missing proteins in the genome. It is likely that further assessment of the genomes would identify the potential missing genes.

Antioxidants

Comparison of *D. melanogaster* proteins with GO terms relating to antioxidant activity and response to oxidative stress to predicted genes of the two sand fly species *Lu. longipalpis* and *Ph. papatasi* allowed for identification of orthologs in each species. Due to a high degree of similarity, 38 of the 65 identified *Lu. longipalpis* orthologs require no revisions while 13 require minor revisions and 14 require large-scale revisions. 31 of the 61 identified *Ph. papatasi* predicted models also require no revisions while 19 require minor revisions and 11 require major revisions (S27 Table). This analysis revealed a likely expansion of proteins with peroxidase and peroxiredoxin functions in both sand fly species analyzed, which was also noted in mosquito species [117-119].

Vitamin Metabolism

A comparison between *D. melanogaster* and the hypothetical scaffolds of *Ph. papatasi* and *Lu. longipalpis* revealed high levels of similarity between the three species genes known to be involved in the in vitamin metabolic processes. In *Ph. papatasi* and *Lu. longipalpis*, a total of 59 and 58 genes were found, respectively, to be potentially associated with vitamin metabolism. High similarity was noted between *D. melanogaster, An. gambiae*and *Ae.aegypti*and the two sand fly species. Only a small percentage of the hypothetical transcripts required revision (marked Y in revision required column in S28 Table).

Novel Viruses

The *Ph. papatasi* and *Lu. longipalpis* genomes are essentially similar in terms of their integrated virus complement. For *Lu. longipalpis* there appear to be traces of ancient bracoviral insertions in to the *Lutozmyia* genome, and perhaps some retroviral sequences. HF586479: Cotesia congregata bracovirus, pos. 140201-140316 is annotated as a “proviral locus”. It is found in a variety of genomes of insects *Dendroctonus ponderosae* (mountain pine beetle), and *Drosophila* but also in *Schistosoma*. EU001284: *Glyptapanteles flavicoxis* (parasitic wasp) bracovirus segment 29, pos. 11878-11995, is also found in various insect genomes. The region of assembly of the *Lu. longipalpis* reads is in a long non-coding 3’ stretch. AJ289710: human endogenous retrovirus (hERV) H, pos. 3187-3452. BXU82084: Bacteriophage X transposon IS2, pos. 872-1204. Phage DNA is frequently inserted into deep sequencing experiments as a quantitative control.

To further investigate the bracoviral insertions, RNAseq *.fq reads were aligned onto the bracovirus fraction alone of GenBank (1303 polydnaviridae family GenBank sequences). Like the *.sff reads, the RNASeq reads aligning to the bracovirus fraction are mostly, with the exception of the hits to EU001284.

Unlike *Lu. longipalpis*, there are several hits to *Wolbachia* phages, indicating probably *Wolbachia* infection of flies used for sequencing, not found in *Lutzomyia*. AB161975: W0CauB1 appears to be present in its entirety and AB036666: WO is nearly complete. HQ906663: wVitA has its entire 5'-half present and HQ906662: wVitA has patchier coverage.

BLAST of transcripts with viral reference genomes: In both *Ph. papatasi* and *Lu. longipalpis*, all matches of longer than 100 nucleotides can be accounted for as cellular genes. In particular, ribonucleoside diphosphate reductase is found in both viral and cellular genomes. Heat shock proteins, thymidylate kinases, signal recognition particles and ubiquitins account for the rest. BLAST of transcripts and genome contigs onto viral GenBank fraction: In *Lu. longipalpis*, 19 transcripts have BLAST hits to retroviral sequences, and 79 to bracoviral sequences. In *Ph. papatasi*, the corresponding figures are 18 and 63 respectively.

**References**

1. Copeland CS, Mann VH, Morales ME, Kalinna BH, Brindley PJ. The Sinbad retrotransposon from the genome of the human blood fluke, Schistosoma mansoni, and the distribution of related Pao-like elements. BMC Evol Biol. 2005;5:20. Epub 2005/02/24. doi: 10.1186/1471-2148-5-20. PubMed PMID: 15725362; PubMed Central PMCID: PMCPMC554778.

2. de la Chaux N, Wagner A. BEL/Pao retrotransposons in metazoan genomes. BMC Evol Biol. 2011;11:154. Epub 2011/06/07. doi: 10.1186/1471-2148-11-154. PubMed PMID: 21639932; PubMed Central PMCID: PMCPMC3118150.

3. Biology IfS. Institute for Systems Biology, RepeatMasker Genomic Datasets 2014 [cited 2016]. Available from: <http://www.repeatmasker.org/species/dm.html>.

4. Boulanger N, Lowenberger C, Volf P, Ursic R, Sigutova L, Sabatier L, et al. Characterization of a defensin from the sand fly Phlebotomus duboscqi induced by challenge with bacteria or the protozoan parasite Leishmania major. Infect Immun. 2004;72(12):7140-6. PubMed PMID: 15557638.

5. Telleria EL, Sant'Anna MR, Alkurbi MO, Pitaluga AN, Dillon RJ, Traub-Cseko YM. Bacterial feeding, Leishmania infection and distinct infection routes induce differential defensin expression in Lutzomyia longipalpis. Parasites & vectors. 2013;6:12. doi: 10.1186/1756-3305-6-12. PubMed PMID: 23311993; PubMed Central PMCID: PMC3573903.

6. Telleria EL, Sant'Anna MR, Ortigao-Farias JR, Pitaluga AN, Dillon VM, Bates PA, et al. Caspar-like gene depletion reduces Leishmania infection in sand fly host Lutzomyia longipalpis. J Biol Chem. 2012;287(16):12985-93. doi: 10.1074/jbc.M111.331561. PubMed PMID: 22375009; PubMed Central PMCID: PMC3339954.

7. Buchon N, Silverman N, Cherry S. Immunity in Drosophila melanogaster--from microbial recognition to whole-organism physiology. Nat Rev Immunol. 2014;14(12):796-810. Epub 2014/11/26. doi: 10.1038/nri3763. PubMed PMID: 25421701.

8. Chang CI, Pili-Floury S, Herve M, Parquet C, Chelliah Y, Lemaitre B, et al. A Drosophila pattern recognition receptor contains a peptidoglycan docking groove and unusual L,D-carboxypeptidase activity. PLoS biology. 2004;2(9):E277. doi: 10.1371/journal.pbio.0020277. PubMed PMID: 15361936; PubMed Central PMCID: PMC515366.

9. Hultmark D. Drosophila immunity: paths and patterns. Curr Opin Immunol. 2003;15(1):12-9. PubMed PMID: 12495727.

10. Kim T, Kim YJ. Overview of innate immunity in Drosophila. Journal of biochemistry and molecular biology. 2005;38(2):121-7. PubMed PMID: 15826489.

11. Kleino A, Silverman N. The Drosophila IMD pathway in the activation of the humoral immune response. Dev Comp Immunol. 2014;42(1):25-35. doi: 10.1016/j.dci.2013.05.014. PubMed PMID: 23721820; PubMed Central PMCID: PMC3808521.

12. Kuraishi T, Hori A, Kurata S. Host-microbe interactions in the gut of Drosophila melanogaster. Frontiers in physiology. 2013;4:375. doi: 10.3389/fphys.2013.00375. PubMed PMID: 24381562; PubMed Central PMCID: PMC3865371.

13. Ha EM, Lee KA, Seo YY, Kim SH, Lim JH, Oh BH, et al. Coordination of multiple dual oxidase-regulatory pathways in responses to commensal and infectious microbes in drosophila gut. Nat Immunol. 2009;10(9):949-57. doi: 10.1038/ni.1765. PubMed PMID: 19668222.

14. Leulier F, Royet J. Maintaining immune homeostasis in fly gut. Nat Immunol. 2009;10(9):936-8. doi: 10.1038/ni0909-936. PubMed PMID: 19692992.

15. Vasta GR. Roles of galectins in infection. Nature reviews Microbiology. 2009;7(6):424-38. doi: 10.1038/nrmicro2146. PubMed PMID: 19444247; PubMed Central PMCID: PMC3759161.

16. Kamhawi S, Ramalho-Ortigao M, Pham VM, Kumar S, Lawyer PG, Turco SJ, et al. A role for insect galectins in parasite survival. Cell. 2004;119(3):329-41. PubMed PMID: 15543683.

17. Jochim RC, Teixeira CR, Laughinghouse A, Mu J, Oliveira F, Gomes RB, et al. The midgut transcriptome of Lutzomyia longipalpis: comparative analysis of cDNA libraries from sugar-fed, blood-fed, post-digested and Leishmania infantum chagasi-infected sand flies. BMC genomics. 2008;9:15. doi: 10.1186/1471-2164-9-15. PubMed PMID: 18194529; PubMed Central PMCID: PMC2249575.

18. Massague J. The transforming growth factor-beta family. Annual review of cell biology. 1990;6:597-641. doi: 10.1146/annurev.cb.06.110190.003121. PubMed PMID: 2177343.

19. Kingsley DM. The TGF-beta superfamily: new members, new receptors, and new genetic tests of function in different organisms. Genes & development. 1994;8(2):133-46. PubMed PMID: 8299934.

20. Santibanez JF, Quintanilla M, Bernabeu C. TGF-beta/TGF-beta receptor system and its role in physiological and pathological conditions. Clinical science. 2011;121(6):233-51. doi: 10.1042/CS20110086. PubMed PMID: 21615335.

21. Ellis JE, Parker L, Cho J, Arora K. Activin signaling functions upstream of Gbb to regulate synaptic growth at the Drosophila neuromuscular junction. Developmental biology. 2010;342(2):121-33. doi: 10.1016/j.ydbio.2010.03.012. PubMed PMID: 20346940.

22. Crampton A, Luckhart S. The role of As60A, a TGF-beta homolog, in Anopheles stephensi innate immunity and defense against Plasmodium infection. Infect Genet Evol. 2001;1(2):131-41. PubMed PMID: 12798028.

23. Luckhart S, Crampton AL, Zamora R, Lieber MJ, Dos Santos PC, Peterson TM, et al. Mammalian transforming growth factor beta1 activated after ingestion by Anopheles stephensi modulates mosquito immunity. Infect Immun. 2003;71(6):3000-9. PubMed PMID: 12761076; PubMed Central PMCID: PMC155698.

24. Di-Blasi T, Telleria EL, Marques C, Couto RM, da Silva-Neves M, Jancarova M, et al. Lutzomyia longipalpis TGF-beta Has a Role in Leishmania infantum chagasi Survival in the Vector. Frontiers in cellular and infection microbiology. 2019;9:71. Epub 2019/04/12. doi: 10.3389/fcimb.2019.00071. PubMed PMID: 30972305; PubMed Central PMCID: PMCPMC6445956.

25. Garrington TP, Johnson GL. Organization and regulation of mitogen-activated protein kinase signaling pathways. Curr Opin Cell Biol. 1999;11(2):211-8. PubMed PMID: 10209154.

26. Keyse SM. Protein phosphatases and the regulation of mitogen-activated protein kinase signalling. Curr Opin Cell Biol. 2000;12(2):186-92. PubMed PMID: 10712927.

27. Luckhart S, Vodovotz Y, Cui L, Rosenberg R. The mosquito Anopheles stephensi limits malaria parasite development with inducible synthesis of nitric oxide. Proc Natl Acad Sci U S A. 1998;95(10):5700-5. PubMed PMID: 9576947; PubMed Central PMCID: PMC20442.

28. Surachetpong W, Singh N, Cheung KW, Luckhart S. MAPK ERK signaling regulates the TGF-beta1-dependent mosquito response to Plasmodium falciparum. PLoS pathogens. 2009;5(4):e1000366. doi: 10.1371/journal.ppat.1000366. PubMed PMID: 19343212; PubMed Central PMCID: PMC2658807.

29. Horton AA, Wang B, Camp L, Price MS, Arshi A, Nagy M, et al. The mitogen-activated protein kinome from Anopheles gambiae: identification, phylogeny and functional characterization of the ERK, JNK and p38 MAP kinases. BMC genomics. 2011;12:574. doi: 10.1186/1471-2164-12-574. PubMed PMID: 22111877; PubMed Central PMCID: PMC3233564.

30. Lu Z, Jiang H. Regulation of phenoloxidase activity by high- and low-molecular-weight inhibitors from the larval hemolymph of Manduca sexta. Insect Biochem Mol Biol. 2007;37(5):478-85. doi: 10.1016/j.ibmb.2007.02.004. PubMed PMID: 17456442; PubMed Central PMCID: PMC2239305.

31. Povelones M, Waterhouse RM, Kafatos FC, Christophides GK. Leucine-rich repeat protein complex activates mosquito complement in defense against Plasmodium parasites. Science. 2009;324(5924):258-61. doi: 10.1126/science.1171400. PubMed PMID: 19264986; PubMed Central PMCID: PMC2790318.

32. Stanley D, Miller J, Tunaz H. Eicosanoid actions in insect immunity. Journal of innate immunity. 2009;1(4):282-90. doi: 10.1159/000210371. PubMed PMID: 20375586.

33. Tootle TL, Spradling AC. Drosophila Pxt: a cyclooxygenase-like facilitator of follicle maturation. Development. 2008;135(5):839-47. doi: 10.1242/dev.017590. PubMed PMID: 18216169; PubMed Central PMCID: PMC2818214.

34. Abdeladhim M, Kamhawi S, Valenzuela JG. What's behind a sand fly bite? The profound effect of sand fly saliva on host hemostasis, inflammation and immunity. Infect Genet Evol. 2014;28:691-703. doi: 10.1016/j.meegid.2014.07.028. PubMed PMID: 25117872.

35. Grespan R, Lemos HP, Carregaro V, Verri WA, Jr., Souto FO, de Oliveira CJ, et al. The protein LJM 111 from Lutzomyia longipalpis salivary gland extract (SGE) accounts for the SGE-inhibitory effects upon inflammatory parameters in experimental arthritis model. Int Immunopharmacol. 2012;12(4):603-10. doi: 10.1016/j.intimp.2012.02.004. PubMed PMID: 22366405; PubMed Central PMCID: PMC3438676.

36. Xu X, Oliveira F, Chang BW, Collin N, Gomes R, Teixeira C, et al. Structure and function of a "yellow" protein from saliva of the sand fly Lutzomyia longipalpis that confers protective immunity against Leishmania major infection. J Biol Chem. 2011;286(37):32383-93. doi: 10.1074/jbc.M111.268904. PubMed PMID: 21795673; PubMed Central PMCID: PMC3173228.

37. Oliveira F, Lawyer P, Kamhawi S, Valenzuela J. Immunity to distinct sand fly salivary proteins primes the anti-Leishmania immune response towards protection or exacerbation of disease. PLoS Negl Trop Dis. 2008;2:e226.

38. Valenzuela JG, Garfield M, Rowton ED, Pham VM. Identification of the most abundant secreted proteins from the salivary glands of the sand fly Lutzomyia longipalpis, vector of Leishmania chagasi. J Exp Biol. 2004;207(Pt 21):3717-29. PubMed PMID: 15371479.

39. Mans BJ, Calvo E, Ribeiro JM, Andersen JF. The crystal structure of D7r4, a salivary biogenic amine-binding protein from the malaria mosquito Anopheles gambiae. J Biol Chem. 2007;282(50):36626-33. doi: 10.1074/jbc.M706410200. PubMed PMID: 17928288.

40. Marzouki S, Abdeladhim M, Abdessalem CB, Oliveira F, Ferjani B, Gilmore D, et al. Salivary antigen SP32 is the immunodominant target of the antibody response to Phlebotomus papatasi bites in humans. PLoS Negl Trop Dis. 2012;6(11):e1911. doi: 10.1371/journal.pntd.0001911. PubMed PMID: 23209854; PubMed Central PMCID: PMC3510156.

41. Abdeladhim M, Jochim RC, Ben Ahmed M, Zhioua E, Chelbi I, Cherni S, et al. Updating the salivary gland transcriptome of Phlebotomus papatasi (Tunisian strain): the search for sand fly-secreted immunogenic proteins for humans. PloS one. 2012;7(11):e47347. doi: 10.1371/journal.pone.0047347. PubMed PMID: 23139741; PubMed Central PMCID: PMC3491003.

42. Lerner EA, Ribeiro JM, Nelson RJ, Lerner MR. Isolation of maxadilan, a potent vasodilatory peptide from the salivary glands of the sand fly Lutzomyia longipalpis. J Biol Chem. 1991;266(17):11234-6. PubMed PMID: 2040631.

43. Morris RV, Shoemaker CB, David JR, Lanzaro GC, Titus RG. Sandfly maxadilan exacerbates infection with Leishmania major and vaccinating against it protects against L. major infection. J Immunol. 2001;167(9):5226-30. PubMed PMID: 11673536.

44. Collin N, Assumpcao TC, Mizurini DM, Gilmore DC, Dutra-Oliveira A, Kotsyfakis M, et al. Lufaxin, a novel factor Xa inhibitor from the salivary gland of the sand fly Lutzomyia longipalpis blocks protease-activated receptor 2 activation and inhibits inflammation and thrombosis in vivo. Arteriosclerosis, thrombosis, and vascular biology. 2012;32(9):2185-98. doi: 10.1161/ATVBAHA.112.253906. PubMed PMID: 22796577; PubMed Central PMCID: PMC3421056.

45. Chagas AC, Oliveira F, Debrabant A, Valenzuela JG, Ribeiro JM, Calvo E. Lundep, a sand fly salivary endonuclease increases Leishmania parasite survival in neutrophils and inhibits XIIa contact activation in human plasma. PLoS pathogens. 2014;10(2):e1003923. doi: 10.1371/journal.ppat.1003923. PubMed PMID: 24516388; PubMed Central PMCID: PMC3916414.

46. Charlab R, Valenzuela JG, Rowton ED, Ribeiro JM. Toward an understanding of the biochemical and pharmacological complexity of the saliva of a hematophagous sand fly Lutzomyia longipalpis. Proceedings of the National Academy of Sciences. 1999;96(26):15155-60.

47. Černá P, Mikeš L, Volf P. Salivary gland hyaluronidase in various species of phlebotomine sand flies (Diptera: psychodidae). Insect biochemistry and molecular biology. 2002;32(12):1691-7.

48. Ribeiro JM, Charlab R, Rowton ED, Cupp EW. Simulium vittatum (Diptera: Simuliidae) and Lutzomyia longipalpis (Diptera: Psychodidae) salivary gland hyaluronidase activity. Journal of medical entomology. 2000;37(5):743-7.

49. Volfova V, Volf P. The salivary hyaluronidase and apyrase of the sand fly Sergentomyia schwetzi (Diptera, Psychodidae). Insect biochemistry and molecular biology. 2018;102:67-74.

50. Volfova V, Hostomska J, Cerny M, Votypka J, Volf P. Hyaluronidase of bloodsucking insects and its enhancing effect on leishmania infection in mice. PLoS neglected tropical diseases. 2008;2(9):e294.

51. Martin-Martin I, Chagas AC, Guimaraes-Costa AB, Amo L, Oliveira F, Moore IN, et al. Immunity to LuloHya and Lundep, the salivary spreading factors from Lutzomyia longipalpis, protects against Leishmania major infection. PLoS Pathog. 2018;14(5):e1007006. Epub 2018/05/04. doi: 10.1371/journal.ppat.1007006. PubMed PMID: 29723281; PubMed Central PMCID: PMCPMC5953502.

52. de Moura TR, Oliveira F, Carneiro MW, Miranda JC, Clarencio J, Barral-Netto M, et al. Functional transcriptomics of wild-caught Lutzomyia intermedia salivary glands: identification of a protective salivary protein against Leishmania braziliensis infection. PLoS Negl Trop Dis. 2013;7(5):e2242. doi: 10.1371/journal.pntd.0002242. PubMed PMID: 23717705; PubMed Central PMCID: PMC3662654.

53. Tavares NM, Silva RA, Costa DJ, Pitombo MA, Fukutani KF, Miranda JC, et al. Lutzomyia longipalpis saliva or salivary protein LJM19 protects against Leishmania braziliensis and the saliva of its vector, Lutzomyia intermedia. PLoS Negl Trop Dis. 2011;5(5):e1169. PubMed PMID: 21655303.

54. Gomes R, Teixeira C, Teixeira M, Oliveira F, Menezes M, Silva C, et al. Immunity to a salivary protein of a sand fly vector protects against the fatal outcome of visceral leishmaniasis in a hamster model. Proc Natl Acad Sci U S A. 2008;105:7845-50.

55. Rohousova I, Subrahmanyam S, Volfova V, Mu J, Volf P, Valenzuela JG, et al. Salivary gland transcriptomes and proteomes of Phlebotomus tobbi and Phlebotomus sergenti, vectors of leishmaniasis. PLoS Negl Trop Dis. 2012;6(5):e1660. doi: 10.1371/journal.pntd.0001660. PubMed PMID: 22629480; PubMed Central PMCID: PMC3358328.

56. Telleria EL, de Araujo AP, Secundino NF, d'Avila-Levy CM, Traub-Cseko YM. Trypsin-like serine proteases in Lutzomyia longipalpis--expression, activity and possible modulation by Leishmania infantum chagasi. PloS one. 2010;5(5):e10697. doi: 10.1371/journal.pone.0010697. PubMed PMID: 20502532; PubMed Central PMCID: PMC2872664.

57. Sant'anna MR, Diaz-Albiter H, Mubaraki M, Dillon RJ, Bates PA. Inhibition of trypsin expression in Lutzomyia longipalpis using RNAi enhances the survival of Leishmania. Parasites & vectors. 2009;2(1):62. PubMed PMID: 20003192.

58. Rawlings ND, Waller M, Barrett AJ, Bateman A. MEROPS: the database of proteolytic enzymes, their substrates and inhibitors. Nucleic Acids Res. 2014;42(Database issue):D503-9. Epub 20131023. doi: 10.1093/nar/gkt953. PubMed PMID: 24157837; PubMed Central PMCID: PMCPMC3964991.

59. Lakomek K, Dickmanns A, Kettwig M, Urlaub H, Ficner R, Lübke T. Initial insight into the function of the lysosomal 66.3 kDa protein from mouse by means of X-ray crystallography. BMC Struct Biol. 2009;9:56. Epub 20090825. doi: 10.1186/1472-6807-9-56. PubMed PMID: 19706171; PubMed Central PMCID: PMCPMC2739207.

60. Yurtsever Z, Sala-Rabanal M, Randolph DT, Scheaffer SM, Roswit WT, Alevy YG, et al. Self-cleavage of human CLCA1 protein by a novel internal metalloprotease domain controls calcium-activated chloride channel activation. J Biol Chem. 2012;287(50):42138-49. Epub 20121030. doi: 10.1074/jbc.M112.410282. PubMed PMID: 23112050; PubMed Central PMCID: PMCPMC3516759.

61. Lombard V, Golaconda Ramulu H, Drula E, Coutinho PM, Henrissat B. The carbohydrate-active enzymes database (CAZy) in 2013. Nucleic Acids Res. 2014;42(Database issue):D490-5. Epub 20131121. doi: 10.1093/nar/gkt1178. PubMed PMID: 24270786; PubMed Central PMCID: PMCPMC3965031.

62. da Costa-Latgé SG, Bates P, Dillon R, Genta FA. Characterization of Glycoside Hydrolase Families 13 and 31 Reveals Expansion and Diversification of α-Amylase Genes in the Phlebotomine. Front Physiol. 2021;12:635633. Epub 20210409. doi: 10.3389/fphys.2021.635633. PubMed PMID: 33897451; PubMed Central PMCID: PMCPMC8063059.

63. Vale VF, Moreira BH, Moraes CS, Pereira MH, Genta FA, Gontijo NF. Carbohydrate digestion in Lutzomyia longipalpis' larvae (Diptera - Psychodidae). J Insect Physiol. 2012;58(10):1314-24. Epub 2012/07/31. doi: 10.1016/j.jinsphys.2012.07.005. PubMed PMID: 22841889.

64. El Kordy EA, Kamel KE, Shoukry MA, Shehata MG. Induction of some digestive enzymes in the midgut of the sandfly Phlebotomus langeroni after sugar and blood meals. J Egypt Soc Parasitol. 1999;29(3):669-86. PubMed PMID: 12561909.

65. Jacobson RL, Schlein Y. Phlebotomus papatasi and Leishmania major parasites express alpha-amylase and alpha-glucosidase. Acta Trop. 2001;78(1):41-9. doi: 10.1016/s0001-706x(00)00164-9. PubMed PMID: 11164750.

66. Terra WR, Ferreira C. Insect digestive enzymes: properties, compartmentalization and function. Comparative Biochemistry and Physiology. 1-62;109(1):1-62. doi: 10.1016/0305-0491(94)90141-4.

67. Menezes HSG, Nascimento NA, Paiva-Cavalcanti M, da Costa-Latgé SG, Genta FA, Oliveira CM, et al. Molecular and biological features of Culex quinquefasciatus homozygous larvae for two cqm1 alleles that confer resistance to Lysinibacillus sphaericus larvicides. Pest Manag Sci. 2021;77(7):3135-44. Epub 20210313. doi: 10.1002/ps.6349. PubMed PMID: 33644981.

68. Zhang J, Zhang X, Arakane Y, Muthukrishnan S, Kramer KJ, Ma E, et al. Comparative genomic analysis of chitinase and chitinase-like genes in the African malaria mosquito (Anopheles gambiae). PloS one. 2011;6(5):e19899. doi: 10.1371/journal.pone.0019899. PubMed PMID: 21611131; PubMed Central PMCID: PMC3097210.

69. Zhu Q, Arakane Y, Beeman RW, Kramer KJ, Muthukrishnan S. Functional specialization among insect chitinase family genes revealed by RNA interference. Proc Natl Acad Sci U S A. 2008;105(18):6650-5. doi: 10.1073/pnas.0800739105. PubMed PMID: 18436642; PubMed Central PMCID: PMC2373347.

70. Giraldo-Calderón GI, Harb OS, Kelly SA, Rund SS, Roos DS, McDowell MA. VectorBase.org updates: bioinformatic resources for invertebrate vectors of human pathogens and related organisms. Curr Opin Insect Sci. 2021;50:100860. Epub 20211203. doi: 10.1016/j.cois.2021.11.008. PubMed PMID: 34864248.

71. Giraldo-Calderon GI, Emrich SJ, MacCallum RM, Maslen G, Dialynas E, Topalis P, et al. VectorBase: an updated bioinformatics resource for invertebrate vectors and other organisms related with human diseases. Nucleic Acids Res. 2015;43(Database issue):D707-13. Epub 2014/12/17. doi: 10.1093/nar/gku1117. PubMed PMID: 25510499; PubMed Central PMCID: PMCPMC4383932.

72. Megy K, Emrich SJ, Lawson D, Campbell D, Dialynas E, Hughes DST, et al. VectorBase: improvements to a bioinformatics resource for invertebrate vector genomics. Nucleic acids research. 2012;40:D729-34. doi: 10.1093/nar/gkr1089. PubMed PMID: 22135296.

73. Merzendorfer H, Zimoch L. Chitin metabolism in insects: structure, function and regulation of chitin synthases and chitinases. J Exp Biol. 2003;206(Pt 24):4393-412. PubMed PMID: 14610026.

74. Leonard R, Rendic D, Rabouille C, Wilson IB, Preat T, Altmann F. The Drosophila fused lobes gene encodes an N-acetylglucosaminidase involved in N-glycan processing. J Biol Chem. 2006;281(8):4867-75. doi: 10.1074/jbc.M511023200. PubMed PMID: 16339150.

75. Zen KC, Choi HK, Krishnamachary N, Muthukrishnan S, Kramer KJ. Cloning, expression, and hormonal regulation of an insect beta-N-acetylglucosaminidase gene. Insect Biochem Mol Biol. 1996;26(5):435-44. PubMed PMID: 8763162.

76. Filho BP, Lemos FJ, Secundino NF, Pascoa V, Pereira ST, Pimenta PF. Presence of chitinase and beta-N-acetylglucosaminidase in the Aedes aegypti. a chitinolytic system involving peritrophic matrix formation and degradation. Insect Biochem Mol Biol. 2002;32(12):1723-9. PubMed PMID: 12429124.

77. Hogenkamp DG, Arakane Y, Kramer KJ, Muthukrishnan S, Beeman RW. Characterization and expression of the beta-N-acetylhexosaminidase gene family of Tribolium castaneum. Insect Biochem Mol Biol. 2008;38(4):478-89. doi: 10.1016/j.ibmb.2007.08.002. PubMed PMID: 18342252.

78. Dixit R, Arakane Y, Specht CA, Richard C, Kramer KJ, Beeman RW, et al. Domain organization and phylogenetic analysis of proteins from the chitin deacetylase gene family of Tribolium castaneum and three other species of insects. Insect Biochem Mol Biol. 2008;38(4):440-51. doi: 10.1016/j.ibmb.2007.12.002. PubMed PMID: 18342249.

79. Arakane Y, Dixit R, Begum K, Park Y, Specht CA, Merzendorfer H, et al. Analysis of functions of the chitin deacetylase gene family in Tribolium castaneum. Insect Biochem Mol Biol. 2009;39(5-6):355-65. doi: 10.1016/j.ibmb.2009.02.002. PubMed PMID: 19268706.

80. Tellam RL, Wijffels G, Willadsen P. Peritrophic matrix proteins. Insect Biochem Mol Biol. 1999;29(2):87-101. PubMed PMID: 10196732.

81. Coutinho-Abreu IV, Sharma NK, Robles-Murguia M, Ramalho-Ortigao M. Characterization of Phlebotomus papatasi peritrophins, and the role of PpPer1 in Leishmania major survival in its natural vector. PLoS Negl Trop Dis. 2013;7(3):e2132. doi: 10.1371/journal.pntd.0002132. PubMed PMID: 23516661; PubMed Central PMCID: PMC3597473.

82. Jasrapuria S, Arakane Y, Osman G, Kramer KJ, Beeman RW, Muthukrishnan S. Genes encoding proteins with peritrophin A-type chitin-binding domains in Tribolium castaneum are grouped into three distinct families based on phylogeny, expression and function. Insect Biochem Mol Biol. 2010;40(3):214-27. doi: 10.1016/j.ibmb.2010.01.011. PubMed PMID: 20144715.

83. Benoit JB, Hansen IA, Szuter EM, Drake LL, Burnett DL, Attardo GM. Emerging roles of aquaporins in relation to the physiology of blood-feeding arthropods. Journal of comparative physiology B, Biochemical, systemic, and environmental physiology. 2014;184(7):811-25. doi: 10.1007/s00360-014-0836-x. PubMed PMID: 24942313.

84. Finn RN, Chauvigne F, Stavang JA, Belles X, Cerda J. Insect glycerol transporters evolved by functional co-option and gene replacement. Nature communications. 2015;6:7814. Epub 2015/07/18. doi: 10.1038/ncomms8814. PubMed PMID: 26183829; PubMed Central PMCID: PMCPMC4518291.

85. Ben-Shahar Y, Leung HT, Pak WL, Sokolowski MB, Robinson GE. cGMP-dependent changes in phototaxis: a possible role for the foraging gene in honey bee division of labor. J Exp Biol. 2003;206(Pt 14):2507-15. PubMed PMID: 12796464.

86. Coste B, Mathur J, Schmidt M, Earley TJ, Ranade S, Petrus MJ, et al. Piezo1 and Piezo2 are essential components of distinct mechanically activated cation channels. Science. 2010;330(6000):55-60. doi: 10.1126/science.1193270. PubMed PMID: 20813920; PubMed Central PMCID: PMC3062430.

87. Denison R, Raymond-Delpech V. Insights into the molecular basis of social behaviour from studies on the honeybee, Apis mellifera. Invertebrate neuroscience : IN. 2008;8(1):1-9. doi: 10.1007/s10158-008-0066-6. PubMed PMID: 18274798.

88. Hardin PE. Molecular genetic analysis of circadian timekeeping in Drosophila. Adv Genet. 2011;74:141-73. doi: 10.1016/B978-0-12-387690-4.00005-2. PubMed PMID: 21924977; PubMed Central PMCID: PMC4108082.

89. Liu L, Leonard AS, Motto DG, Feller MA, Price MP, Johnson WA, et al. Contribution of Drosophila DEG/ENaC genes to salt taste. Neuron. 2003;39(1):133-46. PubMed PMID: 12848938.

90. Montell C. Drosophila TRP channels. Pflugers Archiv : European journal of physiology. 2005;451(1):19-28. doi: 10.1007/s00424-005-1426-2. PubMed PMID: 15952038.

91. Osborne KA, Robichon A, Burgess E, Butland S, Shaw RA, Coulthard A, et al. Natural behavior polymorphism due to a cGMP-dependent protein kinase of Drosophila. Science. 1997;277(5327):834-6. PubMed PMID: 9242616.

92. Rund SS, Bonar NA, Champion MM, Ghazi JP, Houk CM, Leming MT, et al. Daily rhythms in antennal protein and olfactory sensitivity in the malaria mosquito Anopheles gambiae. Sci Rep. 2013;3:2494. doi: 10.1038/srep02494. PubMed PMID: 23986098; PubMed Central PMCID: PMCPMC3756343.

93. Rund SS, Gentile JE, Duffield GE. Extensive circadian and light regulation of the transcriptome in the malaria mosquito Anopheles gambiae. BMC Genomics. 2013;14:218. Epub 20130403. doi: 10.1186/1471-2164-14-218. PubMed PMID: 23552056; PubMed Central PMCID: PMCPMC3642039.

94. Rund SS, Hou TY, Ward SM, Collins FH, Duffield GE. Genome-wide profiling of diel and circadian gene expression in the malaria vector Anopheles gambiae. Proc Natl Acad Sci U S A. 2011;108(32):E421-30. Epub 20110629. doi: 10.1073/pnas.1100584108. PubMed PMID: 21715657; PubMed Central PMCID: PMCPMC3156198.

95. Rund SS, Lee SJ, Bush BR, Duffield GE. Strain- and sex-specific differences in daily flight activity and the circadian clock of Anopheles gambiae mosquitoes. J Insect Physiol. 2012;58(12):1609-19. Epub 20121013. doi: 10.1016/j.jinsphys.2012.09.016. PubMed PMID: 23068991.

96. Marques MD. Biological rhythms and vector insects. Mem Inst Oswaldo Cruz. 2013;108 Suppl 1:59-62. doi: 10.1590/0074-0276130396. PubMed PMID: 24473803; PubMed Central PMCID: PMC4109180.

97. Meireles-Filho AC, Amoretty PR, Souza NA, Kyriacou CP, Peixoto AA. Rhythmic expression of the cycle gene in a hematophagous insect vector. BMC molecular biology. 2006;7:38. doi: 10.1186/1471-2199-7-38. PubMed PMID: 17069657; PubMed Central PMCID: PMC1636064.

98. Peng G, Shi X, Kadowaki T. Evolution of TRP channels inferred by their classification in diverse animal species. Mol Phylogenet Evol. 2014;84C:145-57. doi: 10.1016/j.ympev.2014.06.016. PubMed PMID: 24981559.

99. Zelle KM, Lu B, Pyfrom SC, Ben-Shahar Y. The genetic architecture of degenerin/epithelial sodium channels in Drosophila. G3. 2013;3(3):441-50. doi: 10.1534/g3.112.005272. PubMed PMID: 23449991; PubMed Central PMCID: PMC3583452.

100. Cameron P, Hiroi M, Ngai J, Scott K. The molecular basis for water taste in Drosophila. Nature. 2010;465(7294):91-5. doi: 10.1038/nature09011. PubMed PMID: 20364123; PubMed Central PMCID: PMC2865571.

101. Chen Z, Wang Q, Wang Z. The amiloride-sensitive epithelial Na+ channel PPK28 is essential for drosophila gustatory water reception. The Journal of neuroscience : the official journal of the Society for Neuroscience. 2010;30(18):6247-52. doi: 10.1523/JNEUROSCI.0627-10.2010. PubMed PMID: 20445050.

102. Hofmann F, Feil R, Kleppisch T, Schlossmann J. Function of cGMP-dependent protein kinases as revealed by gene deletion. Physiol Rev. 2006;86(1):1-23. doi: 10.1152/physrev.00015.2005. PubMed PMID: 16371594.

103. Lucas C, Kornfein R, Chakaborty-Chatterjee M, Schonfeld J, Geva N, Sokolowski MB, et al. The locust foraging gene. Archives of insect biochemistry and physiology. 2010;74(1):52-66. doi: 10.1002/arch.20363. PubMed PMID: 20422718.

104. Ben-Shahar Y. The foraging gene, behavioral plasticity, and honeybee division of labor. Journal of comparative physiology A, Neuroethology, sensory, neural, and behavioral physiology. 2005;191(11):987-94. doi: 10.1007/s00359-005-0025-1. PubMed PMID: 16133503.

105. Ben-Shahar Y, Dudek NL, Robinson GE. Phenotypic deconstruction reveals involvement of manganese transporter malvolio in honey bee division of labor. J Exp Biol. 2004;207(Pt 19):3281-8. doi: 10.1242/jeb.01151. PubMed PMID: 15326204.

106. Orgad S, Nelson H, Segal D, Nelson N. Metal ions suppress the abnormal taste behavior of the Drosophila mutant malvolio. J Exp Biol. 1998;201(Pt 1):115-20. PubMed PMID: 9390942.

107. Lutz CC, Robinson GE. Activity-dependent gene expression in honey bee mushroom bodies in response to orientation flight. J Exp Biol. 2013;216(Pt 11):2031-8. doi: 10.1242/jeb.084905. PubMed PMID: 23678099; PubMed Central PMCID: PMC3656508.

108. Liu L, Li Y, Wang R, Yin C, Dong Q, Hing H, et al. Drosophila hygrosensation requires the TRP channels water witch and nanchung. Nature. 2007;450(7167):294-8. doi: 10.1038/nature06223. PubMed PMID: 17994098.

109. Matsuura H, Sokabe T, Kohno K, Tominaga M, Kadowaki T. Evolutionary conservation and changes in insect TRP channels. BMC evolutionary biology. 2009;9:228. doi: 10.1186/1471-2148-9-228. PubMed PMID: 19740447; PubMed Central PMCID: PMC2753570.

110. Modi GB, Tesh RB. A simple technique for mass rearing Lutzomyia longipalpis and Phlebotomus papatasi (Diptera: Psychodidae) in the laboratory. J Med Entomol. 1983;20(5):568-9. PubMed PMID: 6644754.

111. Lehnert BP, Baker AE, Gaudry Q, Chiang AS, Wilson RI. Distinct roles of TRP channels in auditory transduction and amplification in Drosophila. Neuron. 2013;77(1):115-28. doi: 10.1016/j.neuron.2012.11.030. PubMed PMID: 23312520; PubMed Central PMCID: PMC3811118.

112. Fitzpatrick MJ, Ben-Shahar Y, Smid HM, Vet LE, Robinson GE, Sokolowski MB. Candidate genes for behavioural ecology. Trends in ecology & evolution. 2005;20(2):96-104. doi: 10.1016/j.tree.2004.11.017. PubMed PMID: 16701349.

113. Fevereisen, R. Insect CYP, genes and P450 enzymes. In: Gilbert, LI, editors. Insect Molecular Biology and Biochemistry. Amsterdam: Elsevier; 2012. p. 236-316.

114. Vontas J, Katsavou E, Mavridis K. Cytochrome P450-based metabolic insecticide resistance in Anopheles and Aedes mosquito vectors: Muddying the waters. Pestic Biochem Physiol. 2020;170:104666. Epub 20200726. doi: 10.1016/j.pestbp.2020.104666. PubMed PMID: 32980073.

115. Willis JH. Structural cuticular proteins from arthropods: annotation, nomenclature, and sequence characteristics in the genomics era. Insect Biochem Mol Biol. 2010;40(3):189-204. doi: 10.1016/j.ibmb.2010.02.001. PubMed PMID: 20171281; PubMed Central PMCID: PMC2872936.

116. Jindra M, Palli SR, Riddiford LM. The juvenile hormone signaling pathway in insect development. Annu Rev Entomol. 2013;58:181-204. doi: 10.1146/annurev-ento-120811-153700. PubMed PMID: 22994547.

117. Corona M, Robinson GE. Genes of the antioxidant system of the honey bee: annotation and phylogeny. Insect molecular biology. 2006;15(5):687-701. Epub 2006/10/31. doi: 10.1111/j.1365-2583.2006.00695.x. PubMed PMID: 17069640; PubMed Central PMCID: PMCPMC1847502.

118. Neafsey DE, Waterhouse RM, Abai MR, Aganezov SS, Alekseyev MA, Allen JE, et al. Mosquito genomics. Highly evolvable malaria vectors: the genomes of 16 Anopheles mosquitoes. Science. 2015;347(6217):1258522. doi: 10.1126/science.1258522. PubMed PMID: 25554792.

119. Shi GQ, Yu QY, Zhang Z. Annotation and evolution of the antioxidant genes in the silkworm, Bombyx mori. Archives of insect biochemistry and physiology. 2012;79(2):87-103. Epub 2012/03/07. doi: 10.1002/arch.21014. PubMed PMID: 22392770.
