# Supplementary material for: Genomic analysis of two phlebotomine sand fly vectors of Leishmania from the New and Old World
Source: PLoS Negl Trop Dis. 2023 Apr 12;17(4):e0010862. doi: 10.1371/journal.pntd.0010862 (PMC10138862; doi:10.1371/journal.pntd.0010862)
Supplement: S1 Table — (DOCX) [file pntd.0010862.s003.docx]

| **Table S1. Assembly statistics of *Phlebotomus papatasi* and *Lutzomyia longipalpis* draft genome assemblies** | | |
| --- | --- | --- |
| **Feature** | ***P. papatasi*** | ***Lu. longipalpis*** |
| Assembly Version | Ppap_1.0 | Llon_1.0 |
| GenBank Assembly | GCA_000262795.1 | GCA_000265325.1 |
| Assembly Size | 363.768 Mb | 154.229 Mb |
| Gap Length | 18,378,439 | 11,456,874 |
| Spanned Gaps | 32,373 | 24,164 |
| Sequencing coverage | 345.39 Mb | 147.77 Mb |
| Total number of scaffolds | 106,826 | 11,532 |
| Scaffold N50 | 28 kb | 85.1 kb |
| Scaffold L50 | 2,066 | 491 |
| Total number of contigs | 139,199 | 35,696 |
| Contig N50 | 5,795 | 7,481 |
| Contig L50 | 16,042 | 5,523 |
| GC Content | 34.3 % | 35.9 % |
| Gene Set | Ppapl1.4 | LlonJ1.4 |
| # Predicted protein-coding genes | 11,216 | 10,311 |
| % of Genome | 0.003 % | 0.007 % |
| Non-coding genes | 444 | 339 |
| Number of RNAseq transcripts | 11,664 | 10,699 |
| Protein-coding transcripts | 11,220 | 10,330 |
