# Supplementary material for: Genomic analysis of two phlebotomine sand fly vectors of Leishmania from the New and Old World
Source: PLoS Negl Trop Dis. 2023 Apr 12;17(4):e0010862. doi: 10.1371/journal.pntd.0010862 (PMC10138862; doi:10.1371/journal.pntd.0010862)
Supplement: S2 Table — (DOCX) [file pntd.0010862.s004.docx]

| **Table S2. BUSCO assessment** | | |
| --- | --- | --- |
| **BUSCOs** | ***Ph. papatasi*** | ***Lu. longipalpis*** |
| Total | 262 | 261 |
| Complete and single copy | 249 | 238 |
| Complete and duplicate B | 13 | 23 |
| Fragmented | 32 | 9 |
| Missing | 9 | 33 |
| Total searched | 303 | 303 |
| **Completeness** | 86.5% | 86.1% |
