# Supplementary material for: Genomic analysis of two phlebotomine sand fly vectors of Leishmania from the New and Old World
Source: PLoS Negl Trop Dis. 2023 Apr 12;17(4):e0010862. doi: 10.1371/journal.pntd.0010862 (PMC10138862; doi:10.1371/journal.pntd.0010862)
Supplement: S12 Table — (PDF) [file pntd.0010862.s014.pdf]

Peptidase group	Fam.	PFAM	Short Description	Lu. lon	Ph. pap	Aed. aeg.	Anp. gam	Cul. qui	Dro mel	Dro pse	Glo mor
Aspartic	A01	PF00026	Eukaryotic aspartyl protease	4	2	1	1	1	13	13	3
	A22	PF01080	Presenilin	1	1	3	3	4	3	3	3
	A25	PF03418	Germination protease	1	0	0	0	0	0	0	0
	A28	PF09668	Aspartate protease	0	1	1	1	0	1	1	0
	A33	-	skin SASPase	0	1	0	0	0	0	0	0
Cysteine	C01	PF00112	Papain family cysteine protease	5	8	15	13	19	15	12	10
	C02	PF00648	Calpain family cysteine protease	5	4	9	20	7	4	3	3
	C12	PF01088	Ubiquitin carboxyl-terminal hydrolase, family 1	2	3	3	3	3	4	4	4
	C13	PF01650	Peptidase C13 family	1	1	1	1	1	1	1	1
	C14	PF00656	Caspase domain	2	5	9	16	13	7	7	6
	C15	PF01470	Pyroglutamyl peptidase	0	1	1	1	1	1	1	1
	C19	PF00443	Ubiquitin carboxyl-terminal hydrolase	18	17	21	39	29	23	18	24
	C26	PF07722	Peptidase C26	2	1	4	5	4	8	6	7
	C44	PF00310	Glutamine amidotransferases class-II	3	1	5	4	5	7	6	7
	C46	PF01079	Hint module	1	0	1	1	1	1	1	1
	C48	PF02902	Ulp1 protease family, C-terminal catalytic domain	3	3	3	4	3	7	5	9
	C54	PF03416	Peptidase family C54	2	3	2	2	2	2	2	2
	C56	PF01965	DJ-1/PfpI family	2	1	1	2	1	2	1	5
	C64/C85	PF02338	OTU-like cysteine protease	5	3	4	5	4	7	6	4
	C65	PF10275	Peptidase C65 Otubain	1	0	1	1	1	2	1	1
	C67	-	CylD peptidase*	1	1	1	1	1	0	1	1
	C78	PF07910	Peptidase family C78	3	2	2	2	2	2	2	2
	C86	PF02099	Josephin	1	1	0	1	1	1	2	1
	C95	-	lysosomal 66.3 kDa protein	1	1	0	0	0	0	0	0
	C97	PF05903	PPPDE putative peptidase domain	2	2	2	1	2	2	1	1
Metallo	M01	PF01433	Peptidase family M1	17	22	27	39	23	23	24	14
	M02	PF01401	Angiotensin-converting enzyme	5	5	8	11	9	6	7	7
	M03	PF01432	Peptidase family M3	2	2	2	2	2	2	2	2
	M08	PF01457	Leishmanolysin	1	1	1	1	1	1	1	1
	M10	PF00413	Matrixin	3	3	8	4	9	3	2	3
	M12B	PF01421	Reprolysin (M12B) family zinc metalloprotease	5	4	7	13	11	14	7	11
	M13	PF05649	Peptidase family M13	6	5	9	9	9	30	27	25
	M14	PF00246	Zinc carboxypeptidase	20	26	24	38	30	32	28	28
	M16	PF00675	Insulinase (Peptidase family M16)	5	4	10	13	12	12	16	15
	M17	PF00883	Cytosol aminopeptidase family, catalytic domain	6	6	5	5	6	9	13	11
	M19	PF01244	Membrane dipeptidase (Peptidase family M19)	5	6	7	6	4	6	4	4
	M20	PF01546	Peptidase family M20/M25/M40	6	3	1	2	0	7	0	4
	M22	PF00814	Glycoprotease family	2	1	2	2	2	2	4	4
	M23	PF01551	Peptidase family M23	0	0	0	1	0	0	0	0
	M24	PF00557	Metallopeptidase family M24	8	7	11	16	8	11	6	12
	M28	PF04389	Peptidase family M28	5	6	3	13	5	15	9	9
	M38	PF01979	Amidohydrolase family	4	4	0	1	1	4	1	6
	M41	PF01434	Peptidase family M41	3	1	3	3	3	4	3	4
	M48	PF01435	Peptidase family M48	1	1	1	1	1	4	4	1
	M49	PF03571	Peptidase family M49	1	0	0	1	1	1	1	1
	M67	PF01398	JAB1/Mov34/MPN/PAD-1 ubiquitin protease	5	5	5	7	4	8	4	10

	M76	PF09768	Peptidase M76 family	1	1	1	1	1	1	1	1
	M79	PF02517	CAAX protease self-immunity	1	1	1	1	1	1	1	1
	M87	PF08434	Chloride channel accessory protein	1	1	0	0	0	0	0	0
Serine	S01	PF00089	Trypsin	112	126	370	560	428	306	215	221
	S08	PF00082	Subtilase family	6	4	6	5	5	6	5	5
	S09	PF00326	Prolyl oligopeptidase family	7	6	10	18	13	45	11	52
	S10	PF00450	Serine carboxypeptidase	5	3	5	4	5	5	6	4
	S14	PF00574	Clp protease	1	1	1	2	1	1	1	3
	S16	PF05362	Lon protease (S16) C-terminal proteolytic domain	1	0	1	1	1	1	1	3
	S24	PF00717	Peptidase S24	2	1	0	0	0	0	0	0
	S28	PF05577	Serine carboxypeptidase S28	7	5	8	10	9	6	7	2
	S33	PF12697	Alpha/beta hydrolase family	24	17	11	24	11	25	9	13
	S54	PF01694	Rhomboid family	4	4	3	5	3	9	6	7
	S59	PF04096	Nucleoporin autopeptidase	1	1	1	1	1	1	1	1
	S60	PF00405	Transferrin	4	3	6	7	9	5	6	5
	S63	-	EGF-like module containing mucin-like hormone receptor-like 2	1	1	1	1	1	4	1	1
	S72	PF05454	Dystroglycan (Dystrophin-associated glycoprotein 1)	1	1	2	2	2	2	2	2
	S81	PF05497	Destabilase	1	1	2	2	2	7	3	1
Threonine	T01	PF00227	Proteasome subunit	12	13	15	21	18	29	37	21
	T02	PF01112	Asparaginase	3	4	2	3	5	5	4	6
	T03	PF01019	Gamma-glutamyltranspeptidase	2	3	6	3	5	4	4	6
	T06	-	polycystin-1	1	0	0	0	0	0	0	0
	Total*			376	376	703	983	800	808	586	646
