# Supplementary material for: Genomic analysis of two phlebotomine sand fly vectors of Leishmania from the New and Old World
Source: PLoS Negl Trop Dis. 2023 Apr 12;17(4):e0010862. doi: 10.1371/journal.pntd.0010862 (PMC10138862; doi:10.1371/journal.pntd.0010862)
Supplement: S19 Table — (DOCX) [file pntd.0010862.s021.docx]

**Table S19: Details of *Lutzomyia longipalpis* circadian and behavior genes and proteins.** Columns: Gene – the assigned gene and protein name (NTE – N-terminus missing, CTE – C-terminus missing, INT –problems in the assembly, FUS – two gene models located in the same scaffold were fused; JOI – gene model spans scaffolds); OGS – the official gene number in the 10,429 genes in LlonJ1.1, prefix is LLOJ; Scaffold (Sc) – the LlonJ1.1 genome assembly supercontig ID and Contig (Ct) – the LlongJ1 genome assembly contig ID, prefix is Scaffold; Coordinates – the nucleotide range from the first position of the start codon to the last position of the stop codon in the scaffold; Strand + is forward and - is reverse; Introns – number of introns; AAs – number of encoded amino acids in the protein; Comments – comments on the OGS gene model and repairs to be done in the genome assembly.

**Gene OGS Scaffold/Contig Coordinates Strand Introns AAs Comments**

***tim-*JOI** - Sc166 and Sc329 65630-74870 and 106250-107029 - ; + 12 1065 New gene model. LLOTMP002513 (Sc166) and LLOTMP005222 (Sc329) were fused and edited

***tim2*** 002988 Sc188 69219-79438 + 9 1031 Fine as it is

***per-*JOI *-*** Ct49844 and Sc1003 963-1046 and 7944-14194 - ; + 7 1169 New gene model. First exon of the model was localized on Contig49844 (LlonJ1 assembly). The rest of the model was in LLOTMP000134 (Sc1003).

**cwo** 007025 Sc520 47143-50439 - 5 923 Fine as it is

***Clk*-JOI** - Sc48 and Sc108 204225-210403 and 105635-106138 + 7 696 New gene model. LLOTMP006614 (Sc 48) and LLOTMP000502 (Sc108) were fused and edited

***cyc*** 000264 Sc103 104825-112093 + 7 622 Exons 1 to 4 were removed and initial methionine was properly fixed

***sim*-NTE** 000798 Sc1131 44553-56676 + 7 784 NTE region is missed

***tgo*** 003291 Sc2 436819-448928 + 8 621 Final part of the model was edited

***sgg*-CTE** 006077 Sc408 69292-72843 + 1 122 CTE region is missed

***dbt*** 004904 Sc3 424178-439399 + 6 359 Fine as it is

***CKII* *alpha*** 004026 2394 1353-2369 + 0 338 Fine as it is

***CKII beta-*JOI** - Sc2377 and Sc68 11469-13761 and 23168-25415 + 5 277 New gene model. LLOTMP004003 (Sc2377) and LLOTMP008200 (Sc68) were fused

***Pp1a*** - - - - - 162 Present only in the larval L4 transcriptome assembly

***Pp1beta*** 006762 Sc5 106939-123172 - 5 332 Fine as it is

***Pp2a*** 008900 Sc778 60175-68895 - 4 309 First exon was extend and four last exons were excluded

***Pp4*** 003815 Sc227 34843-37174 - 3 307 Fine as it is

***PpV-6*** 10016 Sc979 48828-51945 - 2 302 Maintained only the first three exons of the original model. Third exon extended

***Pp7*-FUS** - Sc874 8757-21833 - 9 695 New gene model. LLOTMP009458 and LLOTMP009459 were fused and edited

***nmo*-NTE** 003956 Sc235 3-10491 + 3 138 NTE region is missed

***cry2*-JOI** - - - - ; + ; - 5 742 New gene model. The initial part of the model was present in Contig06585 (3-1564), followed by Contig12558 (2-202) and Contig06584 (144-974)

**Gene OGS Scaffold/Contig Coordinates Strand Introns AAs Comments**

***Phr*-JOI** - Ct83811 and Sc741 1486-2245 and 34561-36367 + ; + 3 333 New gene model. The first 760 bp of the gene model were in Contig83811. The rest of the gene model was located in LLOTMP008682 (Sc741)

***norpA*** 001939 Sc1440 37196- 45904 - 8 1081 Fine as it is

***vri*** 007416 Sc568 62113-76422 + 2 469 Fine as it is

***Pdp1*** 006612 Sc48 18671-106058 + 4 267 Initial methionine was fixed including a new exon

***slmb*** 006764 Sc5 129351-138574 + 10 700 Fine as it is

***cac*-INT** 006699 Sc489 105764-130064 - 20 1185 Multiple changes

***Na*-FUS** - Sc137 64371-83060 - 10 3074 New gene model. LLOTMP001706 and LLOTMP001707 were fused and edited

***para*-NTE** 007479 Sc576 62487-85952 - 27 1600 Multiple changes

***slo*-NTE** 001559 Sc132 16087-51527 + 19 1018 NTE region is missed

***Slob*** 008864 Sc770 8281-9598 + 2 158 First, second and last exons were eliminated

***nocte*-CTE** 004544 Sc268 88697-92269 + 3 574 CTE region is missed

***Atax-2*** 004561 Sc269 109694-126353 - 9 935 Fine as it is

***ctrip*** 000138 Sc1003 20136-40553 - 11 2100 Exons 7^th^ and 10^th^ were edited

***to1*** 006030 Sc401 20817-21728 - 2 189 LLOTMP006030 was divided in two gene models (*to1* and *to2*). Last exon extended

***to2*** - Sc401 18743-19697 - 3 252 New gene model. First exon added and 2^nd^ exon extended

***to3*** 008362 Sc7 47865-49626 - 3 244 LLOTMP008362 was divided in two different models (*to3* and *to4*); last exon extended

***to4*-NTE** - Sc7 40986-41804 - 1 192 New gene model

***to5*** 006987 Sc514 25417-28591 - 2 248 Fine as it is

***to6*** 008792 Sc76 114913-117338 - 1 219 Fine as it is

***Rh3*** 005188 Sc326 40469 - 47369 + 2 380 The 3rd exon has been eliminated

***Rh7*** 009159 Sc813 59510 60663 + 2 325 Fine as it is

**P*iezo*-NTE** 005459 Sc346 1205-5153 + 3 682 NTE region is missed

***nompC*** 003746 Sc223 5365-38664 + 17 1928 Fine as it is

**pain-INT** 006694 Sc489 4579-1227 + 5 763 Multiple changes

***TrpA1*** 004961 Sc302 27240- 43231 + 13 1273 Fine as it is

***wtwr*** 009849 Sc941 53070-57180 + 5 971 Fine as it is

***wtwr*** 007806 Sc1655 3447-6841 + 1 997 Fine as it is

***wtwr*** 007983 Sc6425 8892 - 11961 - 3 977 Fine as it is

***wtwr*** 006605 Sc4796 21-545 + 2 988 Fine as it is

***wtwr*** 009848 Sc941 44585- 47985 + 2 979 Fine as it is

***trp*-JOI/CTE** - Sc1365 and Sc1033 46030-48856; 18801-23064 + ; + 17 902 New gene model. LLOTMP001696 (Sc1365) and LLOTMP000286 (Sc1033) were fused.

***trpgamma*-NTE** 006929 Sc508 80435-84475 - 4 591 The initial methionine is missed

***Trpl*-NTE** - Sc118 176287-179149 + 2 331 New gene model. The initial methionine is missed.

***Trpm*-NTE/INT** 007263 Sc552 32964-65802 - 24 1654 Initial methionine is missed and internal problems were detected

***Trpml*** 000170 Sc655 161373-163489 + 1 655 Fine as it is

***ppk3*** 003220 Sc198 22341-23961 - 2 487 Fine as it is

**Gene OGS Scaffold/Contig Coordinates Strand Introns AAs Comments**

***pkk9*** - Sc88 17945 - 176385 + 1 458 New gene model

***ppk4*** 007095 Sc530 14760-16306 - 3 451 Fine as it is

***ppk13*-FUS** - Sc2154 16046 -34392 + 9 448 LLOTMP003631 and LLOTMP003631were fused and edited

***ppk16*** 004198 Sc2475 17735-20905 - 2 530 Fine as it is

***ppk16-like*** 005025 Sc31 42322-47258 + 6 915 Fine as it is

***ppk26*** 009835 Sc94 68591-78879 + 6 787 Fine as it is

***ppk26-like*** 001025 Sc1188 22343-30678 - 4 575 Fine as it is

***ppk28*** 001020 Sc1187 19039-28307 + 4 504 Fine as it is

***ppk31*-NTE/CTE** 007955 Sc639 19353-20752 - 3 339 Partial model. CTE and NTE regions were missed

***ppk100*** 005117 Sc32 206361-210069 + 4 532 Fine as it is

***ppk101*** 009834 Sc94 56855-64178 - 5 533 Fine as it is

***ppk102* -**  Sc94 60671-64403 - 2 547 New gene model

***ppk*-NTE -**  Sc299 61443-63745 - 3 404 New gene model.NTE region was missed

***mlv*** 004951 Sc301 25631-32046 - 11 565 Fine as it is

***Pkg2*-1D** 001613 Sc134 123869-138596 - 7 1022 Fine as it is

***for*-NTE** 007793 Sc61 40100- 48763 - 7 515 NTE region is missed. The last seven exons were eliminated

***sr*-CTE** 003205 Sc970 5116-8025 + 0 184 CTE region was extended

**Official full names:**

*tim*: timeless

*per*: period

*cwo*: clockwork orange

Clk: clock

*cyc*: cycle

*sim*: Single minded

*tgo*: tango

*sgg*: shaggy

*dbt*: doubletime

*CKII alpha*: Casein kinase II alpha

*CKII beta*: Casein kinase II beta

*Pp1a:* serine/threonine-protein phosphatase 1 alpha

*Pp2a:* serine/threonine-protein phosphatase 2 alpha

*Pp4:* serine/threonine-protein phosphatase 4

*PpV-6:* serine/threonine-protein phosphatase V-6

*Pp7:* serine/threonine-protein phosphatase 7

*nmo*: nemo

*cry2*: Cryptochorme2

*phr*: DNA photolyase, photorepair (phr)

*norpA*: Phosphoinositide phospholipase C

*vri*: Vrille

*Pdp1*: Par-domain protein1

*slmb*: Supernumerary limbs

*cac*: cacophony

*na*: narrowabdomen

*para*: paralytic

*slo*: Calcium-activated potassium channel slowpoke

*slob*: Slowpoke binding protein

*nocte*: No circadian temperature entrainment

*Atax*-2: Ataxin-2

*ctrip*: Circadin trip

*to*: JHBP/takeout

*Rh3*: Ultraviolet sensitive opsin

*LWO*: long-wavelength opsin

*Rh7*: Rhodopsin

*nompC*: No mechanoreceptor potential C

*pain*: painless

*TrpA1*: transient receptor potential A

*wtrw*: water witch

*trp*: transient receptor potential protein

*trp* gamma: transient receptor potential gamma

*trpL*: transient receptor potential L

*trpm*: transient receptor potential cation channel, melastatin subfamily

*trpml*: transient receptor potential cation channel, mucolipin subfamily

*ppk*: pickpocket

*mlv*: malvolio

*Pkg2-1D*: cGMP-dependent protein kinase, isozyme 1

*for*: *foraging* or cGMP-dependent protein kinase, isozyme 2

*sr*: stripe
