# Supplementary material for: Genomic analysis of two phlebotomine sand fly vectors of Leishmania from the New and Old World
Source: PLoS Negl Trop Dis. 2023 Apr 12;17(4):e0010862. doi: 10.1371/journal.pntd.0010862 (PMC10138862; doi:10.1371/journal.pntd.0010862)
Supplement: S20 Table — (DOCX) [file pntd.0010862.s022.docx]

**Table S20: Detailed information about *Phlebotomus papatasi* circadian and behavior genes and proteins.** Columns: *Gene* – assigned gene and protein (NTE – N-terminus missing, CTE – C-terminus missing, INT – problems in the assembly, FUS – two gene models located in the same scaffold were fused; JOI – gene model spans scaffolds); OGS – the official gene number in the 12,678 genes in Ppapl1.1, prefix is PPAI; Scaffold (Sc) – the Ppapl1.1 genome assembly supercontig ID and Contig (Ct) – the Ppapl1 genome assembly contig ID, prefix is Scaffold; Coordinates – nucleotide range from the first position of the start codon to the last position of the stop codon in the scaffold; Strand + is forward and - is reverse; Introns – number of introns; AAs – number of encoded amino acids in the protein found in the gene model; Comments – comments about the OGS gene model, and repairs that were done in the genome assembly.

**Gene OGS Scaffold/Contig Coordinates Strand Introns AAs Comments**

***tim*-FUS** 012818 Sc47 137910-156773 - 13 1201 PPATMP008008 and PPATMP008007 were fused

***tim2*-JOI** - Sc597 3338-4648 - 9 969 New gene model. It was distributed in Sc597, Scaffold44752 and Scaffold52134

***per*-JOI** 006484 Ct101627.1 and Sc006484 4747-9741 and 350-435 + ; - 7 1158 1^st^ exon was located in Contig101627.1 and the rest of the model was in Scaffold4185 (PPATMP006484)

***cwo*-INT** 012819 Sc430 2201-11463 - 2 619 Manual edition

***Clk*** 002844 Sc2 440906-446226 + 6 706 Fine as it is

***cyc*** 010392 Sc77 344459-357605 + 7 634 1^st^ exon was eliminated and the initial methionine fixed

***sim-*NTE/CTE** 001673 Sc15 618709-626223 - 3 240 The NTE and CTE regions were missed

***tgo***- **NTE/CTE** 009623 Sc61 60289-66121 - 6 556 The NTE and CTE regions were missed

***sgg-*NTE** 010158 Sc72 200643-211489 + 4 331 The NTE region was missed

***dbt***- **NTE** 007687 Sc45652 3543-4142 + 0 199 The NTE region was missed

***CKII alpha*** 000516 Sc112 71465-72481 - 0 338 Fine as it is

***CKII beta-*FUS** 011154 Sc996 9684-20145 + 5 227 New gene model. PPATMP011154 and PPATMP011154 were fused and edited

***Pp1a*-NTE** 005749 Sc3582 603-11379 + 2 163 The NTE region was missed

***Pp2a-* NTE/CTE** 006622 Sc42725 17875-18809 + 1 216 The NTE and CTE regions were missed

***Pp2b*-FUS/CTE** 000839/?000840 Sc1211 15663-39415 + 6 401 New gene model. PPATMP000839 and PPATMP000840 were fused. Note there are still and PPAI000839 & PPAI000840

***PpV-6*-NTE** 003603 Sc23515 3045-4044 + 1 247 The NTE region is missed

***Pp7*-JOI/NTE** 009968/?006145 Sc672 and Sc3936 26965-35214 and 7053-7637 + 3 376 PPATMP009968 (Sc672) and PPATMP006145 (Sc3936) were fused and edited. NTE region was missed Note there is still PPAI009968 & PPAI006145

***nmo*-FUS/NTE** 012817 Sc594 27097-44269 + ; - 6 360 New gene model. PPATMP009517 and PPATMP009518 were fused

***cry1-*NTE** 002137 Sc1671 26175-26932 - 0 253 Model is partial and only contains the last exon

***cry2***-**JOI** 012817 Sc46912 and Sc2983 2643-4050, 301-1506 + ; - 4 743 New gene model. PPATMP009517 (Sc46912) and PPATMP009518 (Sc2983) were fused.

***phr*** 004254 Sc261 63214-64919 + 1 515 Fine as it is

**Gene OGS Scaffold/Contig Coordinates Strand Introns AAs Comments**

***norpA*-JOI** 007015/?007016 Sc4354 and Sc64711 - - 5 801 New gene model IN Sc4354 two predicted protein (PPATMP007015 and PPATMP007016) were fused. In Sc 64711 a new predicted protein was created. Note that PPAI007015 and PPAI007016 are both still there.

***vri*-JOI** ?????? Sc26106 and Sc64592 222-977 and 189-764 - 0 344 New gene model

***Pdp1*-NTE** 002842 Sc2 <273251-281247 + 2 190 NTE region was missed

***slmb-*INT** 004879 Sc299 7110-7561 + 0 150 Assembly problem

***cac*-NTE** 012826 Sc809 835-1791 - 2 267 Model is partial and only contains three exons; first four exons of the original model were deleted.

***na*-CTE** 008229 Sc48310 2841->8148 + 2 1122 CTE region is missed

***para*-JOI** 003387/?394&95 Sc2248 and Sc1094 - - , - ,+, + 16 980 New gene model. Four gene models were fused: PPATMP003387 (coordinates: 9174-14426) and PPATMP003386 (coordinates: 1151-7841) located in Sc2248; PPATMP000394 (coordinates: 7644-14047) and PPATMP000395 (coordinates: 26395-28039) located in Sc1094. Multiple assembly problems. Note all three IDs still there, please check.

***slo*-JOI/CTE** - Multiple - +, - , - ,+ ,+ 10 626 New gene model. Five gene models were fused and edited. PPATMP007684 (Sc45640, coordinates 5741-6062); PPATMP009501 (Sc5903, coordinates: 3467-4002); PPATMP003816 (Sc2407, coordinates: 2902-24213); PPATMP005694 (Sc3539, coordinates: 5161-8967) and PPATMP006643 (Sc42743, coordinates: 3497-9448) All still there

***nocte*-NTE** 002962 Sc2032 <3916-11712 + 3 1660 NTE region was missed

***Atax-2*** 004801 Sc2931 2462-12528 - 3 401 CTE region was extended

***ctrip*-FUS** 012822 Sc816 50562-51523 + 4 1684 New gene model. Two gene models (PPATMP010549 and PPATMP010550) were fused and edited

***to1*-NTE** 007441 Sc44847 <7218-7718 + 0 166 NTE region is missed

***to2*** 012824 Sc1162 <11478-11916 - 0 145 PPATMP000647 was divided in two models (*to2* and *to3*). Initial methionine was missed

***to3-*NTE** ?????? Sc1162 <6974-7842 - 2 225 New gene model. Initial methionine was missed.

***to4-*NTE** 000648 Sc1162 <20371-21129 - 1 225 PPATMP000648 was splitted in two models (*to4* and *to5*); Initial methionine was missed.

***to5-*NTE** ?????? Sc1162 <17873-18785 - 2 226 New gene model. Initial methionine was missed

***Rh3*-FUS** 012839/?012816 Sc2 1226541-1240886 - 2 379 Two gene models (PPATMP002883-RA and PPATMP002881) were fused and edited Note: 2 different IDs map

***LWO*** 004207 Sc26 547523-549089 + 1 381 Fine as it is

***Rh7-*INT** 007252 Sc44163 ? +; - 1 136 Gene model was edited. Assembly problems

***Piezo-*INT** 012821 Sc38 253661-462668 - 19 1959 Multiple changes

***iav*** 004901 Sc3 279511-291919 + 5 1135 Multiple changes

**Gene OGS Scaffold/Contig Coordinates Strand Introns AAs Comments**

***nan*-JOI** 007810 Sc4610 and Sc47892 4988-? - ; + 4 835 New gene model. PPATMP007810 (Sc4610) and Contig8123.1 and Contig190787.1 (Sc47892) were fused.

***pain*** 003852 Sc2424 160-2844 + 0 2685 Fine at it is

***TrpA1*-NTE** 004036 Sc2513 <8698-20514 - 9 1119 NTE region was missed

***wtrw*** 008786 Sc52 76321-79438 + 1 1003 Fine at it is

***wtrw*** 008787 Sc52 99530-102726 + 1 979 One extra exon was added at NTE region

***wtrw*** 008788 Sc52 114914-117964 + 1 993 Fine at it is

***trp-*JOI** 005961 Sc377 - ? ? 707 Manually created. Split in many different scaffolds. Partial model with CTE and NTE regions missed

***trp gamma-*FUS/NTE**00775? Sc12 <491600-498054 - 3 581 Two gene models (PPATMP00775 and PPATMP00776) were fused and edited. CONFUsing, please check.

***trpL*** 009654 Sc615 2582-9933 + 5 1019 Fine as it is

***trpm-*NTE** ????? Sc46755, Sc5448, Sc50709 - ? ? 681 Manually created. NTE region is missed

***trpml*** ????? Sc43374, Sc87076, Sc27005 - ? ? 635 Manually created

***ppk3-*NTE** ????? Sc348 <79190-80437 - 0 415 NTE region was missed

***ppk16*** 006353 Sc4085 2373-9139 - 4 526 Four exons were added at CTE region

***ppk16-like*** 002706 Sc191 136896-138806 - 2 575 Fine as it is

***ppk26-likeA*** 009365 Sc574 29919-35026 + 3 583 Fine as it is

***ppk26-likeB-*INT** 009363 Sc574 29919-35026 + 3 583 Problems in the assembly of the scaffold were detected

***ppk23*** 001398 Sc14 76160-80084 - 2 552 Fine as it is

***ppk28*** 004084 Sc2541 4922-12752 - 5 561 Multiple changes

***ppk31a-*NTE/CTE** 009142 Sc5514 <321->4531 - 2 323 1^st^ exon eliminated and the last exon extended

***ppk31b-*NTE/CTE** 003967 2495 <21580->22580 + 1 266 1^st^ exon eliminated and the last exon extended

***ppk100*** 001000 127 21689-25364 + 4 536 Fine as it is

***ppk101*** 000980 1261 6128-12698 + 2 516 Fine as it is

***ppk102-*CTE** 000978 1261 3674->4681 + 1 316 The last exon eliminated

***ppk-like-*NTE** 012838 42794 <5679-6785 + 1 344 Two first exons were eliminated. NTE region was missed

***mlv-*NTE/CTE** ????? 24350 >641-<1568 - 2 251 New gen model. Partial model

***Pkg2-1D*** 006090 39 103022-112773 - 6 812 Fine as it is

***sr-*NTE** 005020 302 >26270-69382 - 3 847 The initial methionine was missed. Multiple changes

**Official full names:**

*tim*: timeless

*per*: period

*cwo*: clockwork orange

Clk: clock

*cyc*: cycle

*sim*: Single minded

*tgo*: tango

*sgg*: shaggy

*dbt*: doubletime

*CKII alpha*: Casein kinase II alpha

*CKII beta*: Casein kinase II beta

*Pp1a:* serine/threonine-protein phosphatase 1 alpha

*Pp2a:* serine/threonine-protein phosphatase 2 alpha

*Pp2a:* serine/threonine-protein phosphatase 2 beta

*PpV-6:* serine/threonine-protein phosphatase V-6

*Pp7:* serine/threonine-protein phosphatase 7

*nmo*: nemo

*cry1*: Cryptochorme1

*cry2*: Cryptochorme2

*phr*: DNA photolyase, photorepair (phr)

*norpA*: Phosphoinositide phospholipase C

*vri*: Vrille

*Pdp1*: Par-domain protein1

*slmb*: Supernumerary limbs

*cac*: cacophony

*na*: narrowabdomen

*para*: paralytic

*slo*: Calcium-activated potassium channel slowpoke

*nocte*: No circadian temperature entrainment

*Atax*-2: Ataxin-2

*ctrip*: Circadin trip

*to*: JHBP/takeout

*Rh3*: Ultraviolet sensitive opsin

*LWO*: long-wavelength opsin

*Rh7*: Rhodopsin

*pain*: painless

*TrpA1*: transient receptor potential A

*wtrw*: water witch

*trp*: transient receptor potential protein

*trp* gamma: transient receptor potential gamma

*trpL*: transient receptor potential L

*trpm*: transient receptor potential cation channel, melastatin subfamily

*trpml*: transient receptor potential cation channel, mucolipin subfamily

*ppk*: pickpocket

*mlv*: malvolio

*Pkg2-1D*: cGMP-dependent protein kinase, isozyme 1

*sr*: stripe
