# Supplementary material for: Genomic analysis of two phlebotomine sand fly vectors of Leishmania from the New and Old World
Source: PLoS Negl Trop Dis. 2023 Apr 12;17(4):e0010862. doi: 10.1371/journal.pntd.0010862 (PMC10138862; doi:10.1371/journal.pntd.0010862)
Supplement: S33 Table — (DOCX) [file pntd.0010862.s035.docx]

| **Table S33: Parameter values of male copulatory songs from *Lutzomia longipalpis* from Araci and Olindina** | | | | |
| --- | --- | --- | --- | --- |
|  | **IPI / IBI (ms)** | **TL (s)** | **NB** | **FREQ** |
| **Araci** | 57.8 ±4.6 | 2.1 ±0.7 | 36.4 ±11.3 | 242.3 ±12.0 |
| **Olindina** | 283.4 ±45.2 | 3.1 ±0.7 | 11.5 ±1.9 | 272.8 ±10.2 |
| N, number of samples; IPI, inter-pulse interval; IBI, inter-burst interval; TL, train length; NB, number of bursts per train; NP, number of pulses per train; Freq, carrier frequency. Mean (±SE) values are presented. | | | | |
