# Supplementary figures and images for: Genomic analysis of two phlebotomine sand fly vectors of Leishmania from the New and Old World
Source: PLoS Negl Trop Dis. 2023 Apr 12;17(4):e0010862. doi: 10.1371/journal.pntd.0010862 (PMC10138862; doi:10.1371/journal.pntd.0010862)

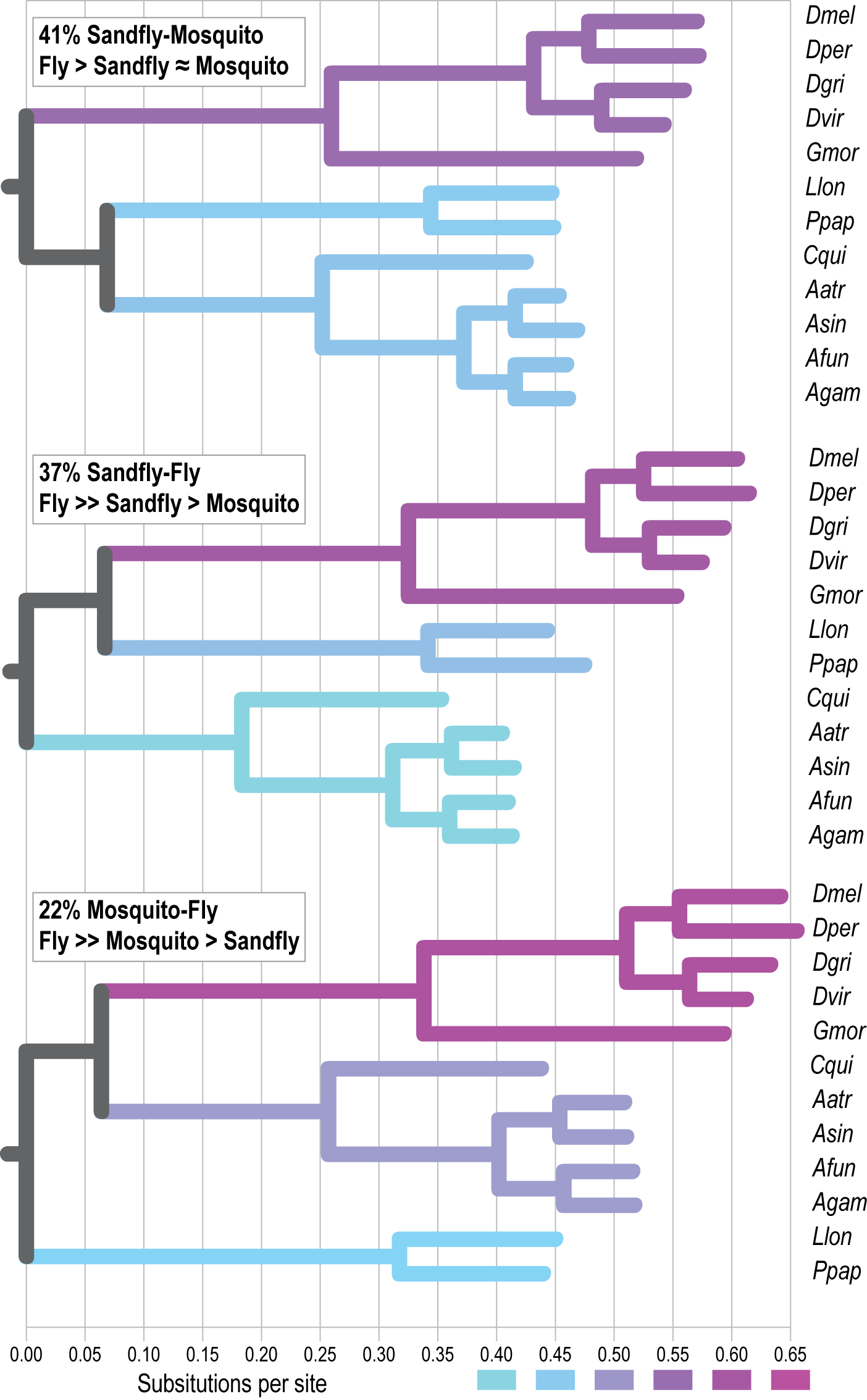

Supplement: S1 Fig — Analysis of the gene phylogenies of individual orthologous groups identified three major topologies with sand fly-mosquito (41%), sand fly-fly (37%), or mosquito-fly (22%) sister clades. Comparisons of average branch lengths for each topology suggest that, although substitution rates in flies are always higher, orthologs that support the sand fly-mosquito topology show the lowest substitution rates in flies and the smallest differences in substitution rates among the fly, sand fly, and mosquito clades. In contrast, the sand fly-fly and mosquito-fly topologies show much higher substitution rates in flies and much greater differences in substitution rates among the three clades. (TIF) [file pntd.0010862.s037.tif]

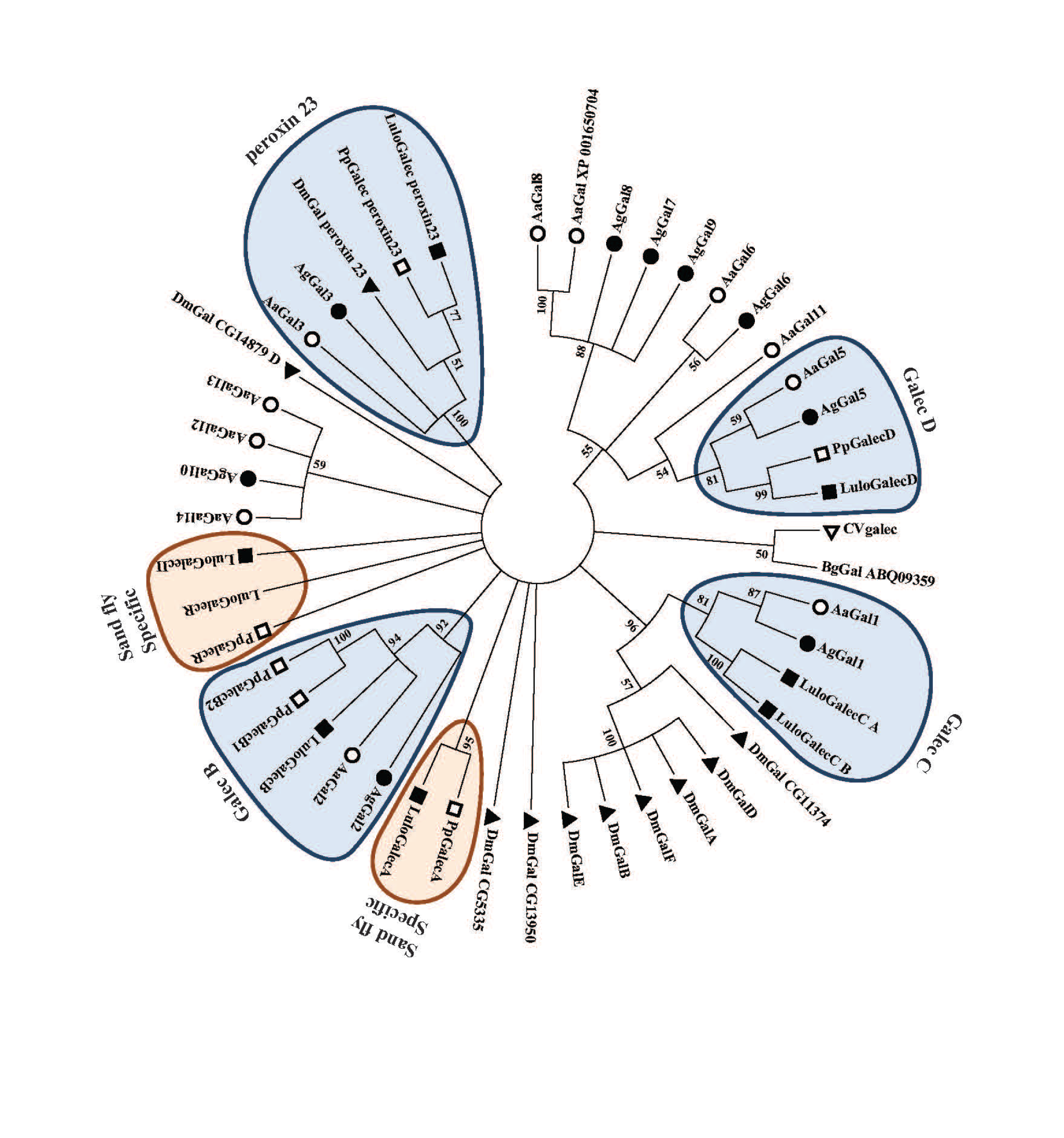

Supplement: S2 Fig — Condensed Neighbor-Joining tree depicting clustering among galectin protein sequences of sand flies (Ph. papatasi and Lu. longipalpis; open and filled squares, respectively), mosquitoes (Ae. aegypti and An. gambiae; open and filled circles, respectively), fly (D. melanogaster; filled triangle), eastern oyster (C. virginica; upside-down open triangle), and freshwater snail (B. glabrata; upside-down filled triangle). Branches encompassing shared orthologs are highlighted by blue shades. Sand fly specific clusters and genes are highlighted by orange shades. The evolutionary distances were computed using the p-distance method and are in the units of the number of amino acid differences per site. One thousand bootstrap replicates were performed, and only branches displaying at least 50% confidence are shown. (TIF) [file pntd.0010862.s038.tif]

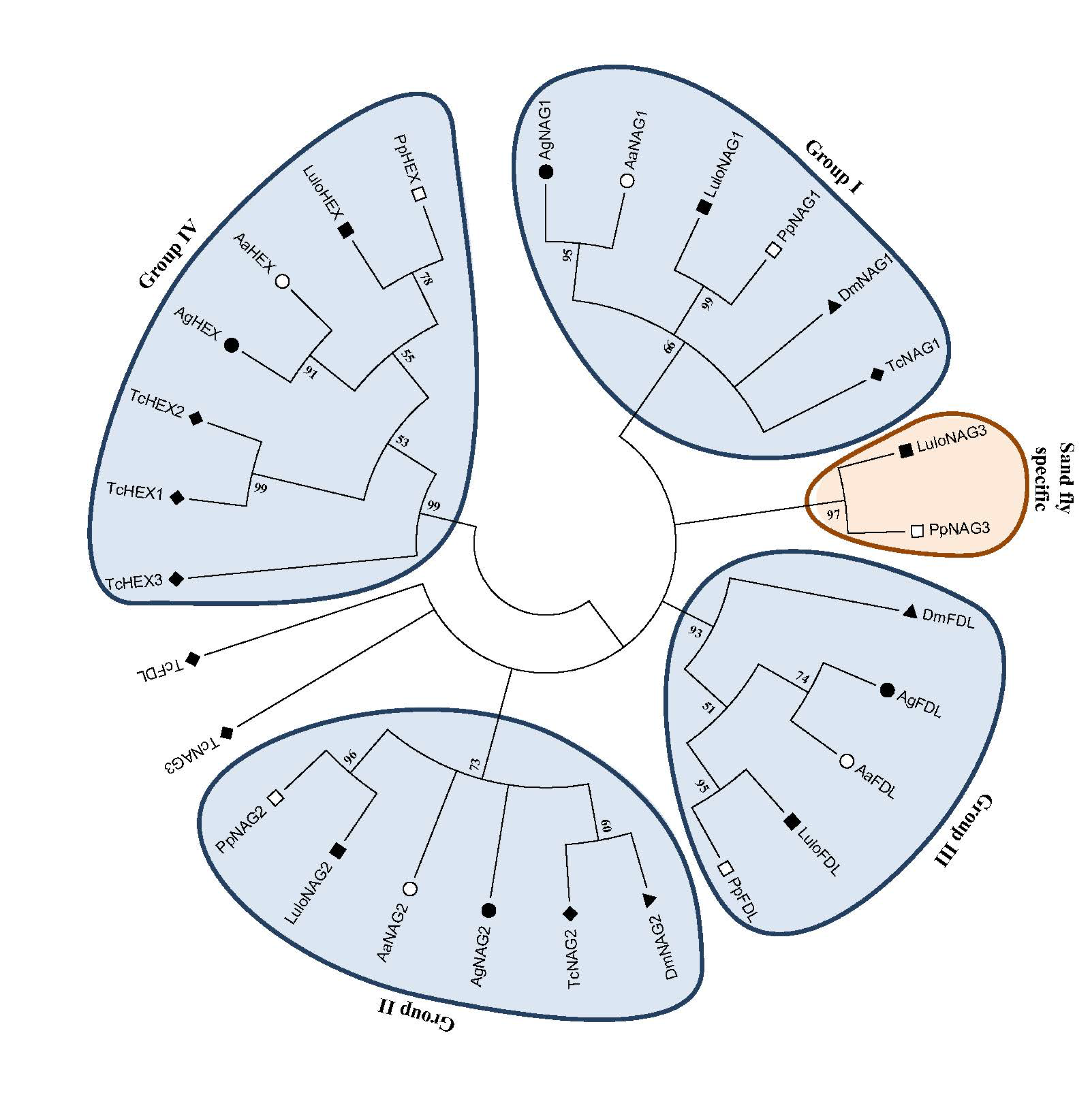

Supplement: S3 Fig — Branches encompassing sequences belonging to group I-IV n-acetylhexosaminidases are highlighted by a blue shade. The sand fly specific cluster is highlighted by an orange shade. The evolutionary distances were computed using the p-distance method and are in the units of the number of amino acid differences per site. One thousand bootstrap replicates were performed, and only branches displaying at least 50% confidence are shown. (TIF) [file pntd.0010862.s039.tif]

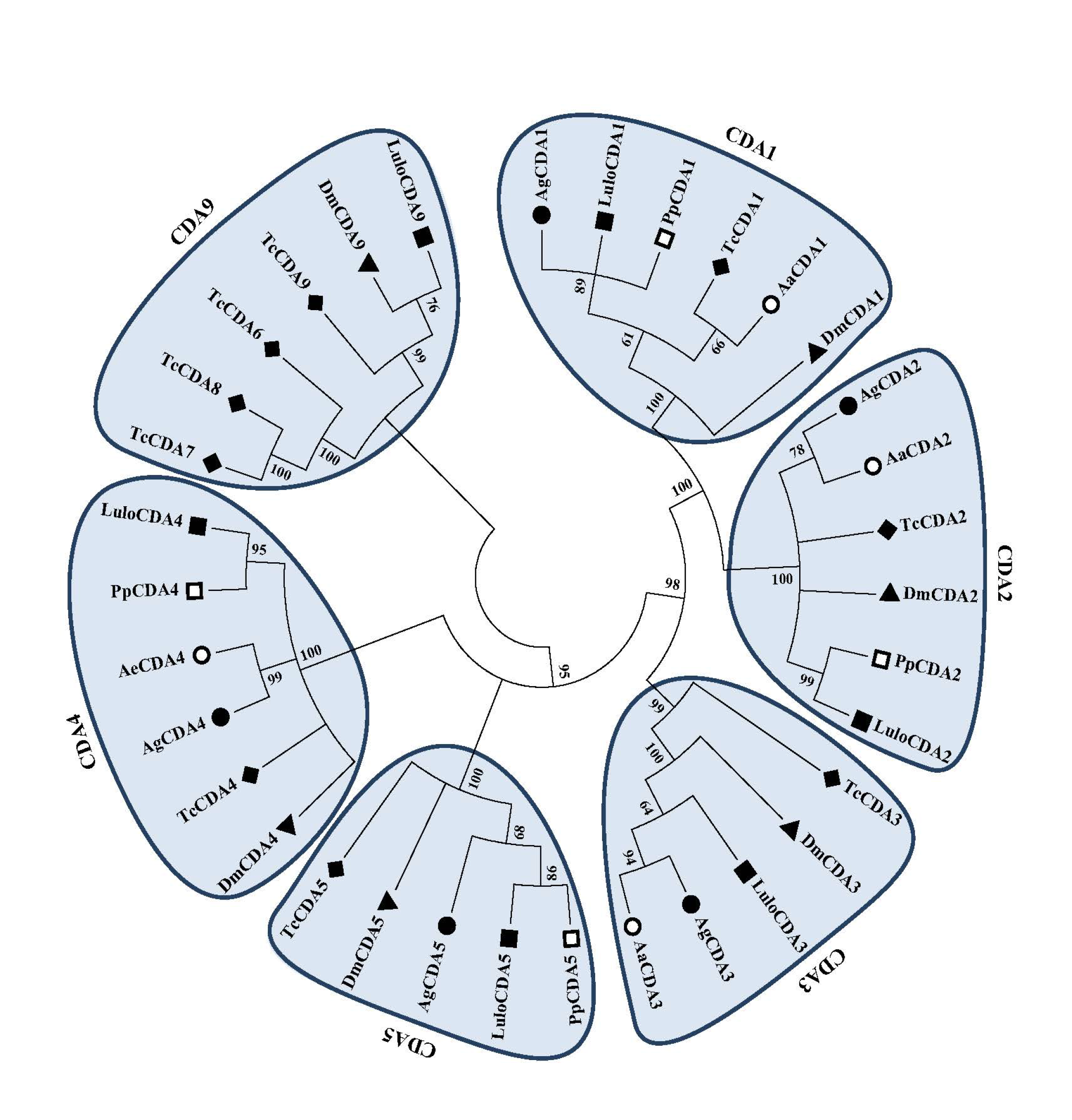

Supplement: S4 Fig — Branches encompassing sequences belonging to group 1–5 and 9 CDA are highlighted by blue shades. The evolutionary distances were computed using the p-distance method and are in the units of the number of amino acid differences per site. One thousand bootstrap replicates were performed, and only branches displaying at least 50% confidence are shown. (TIF) [file pntd.0010862.s040.tif]

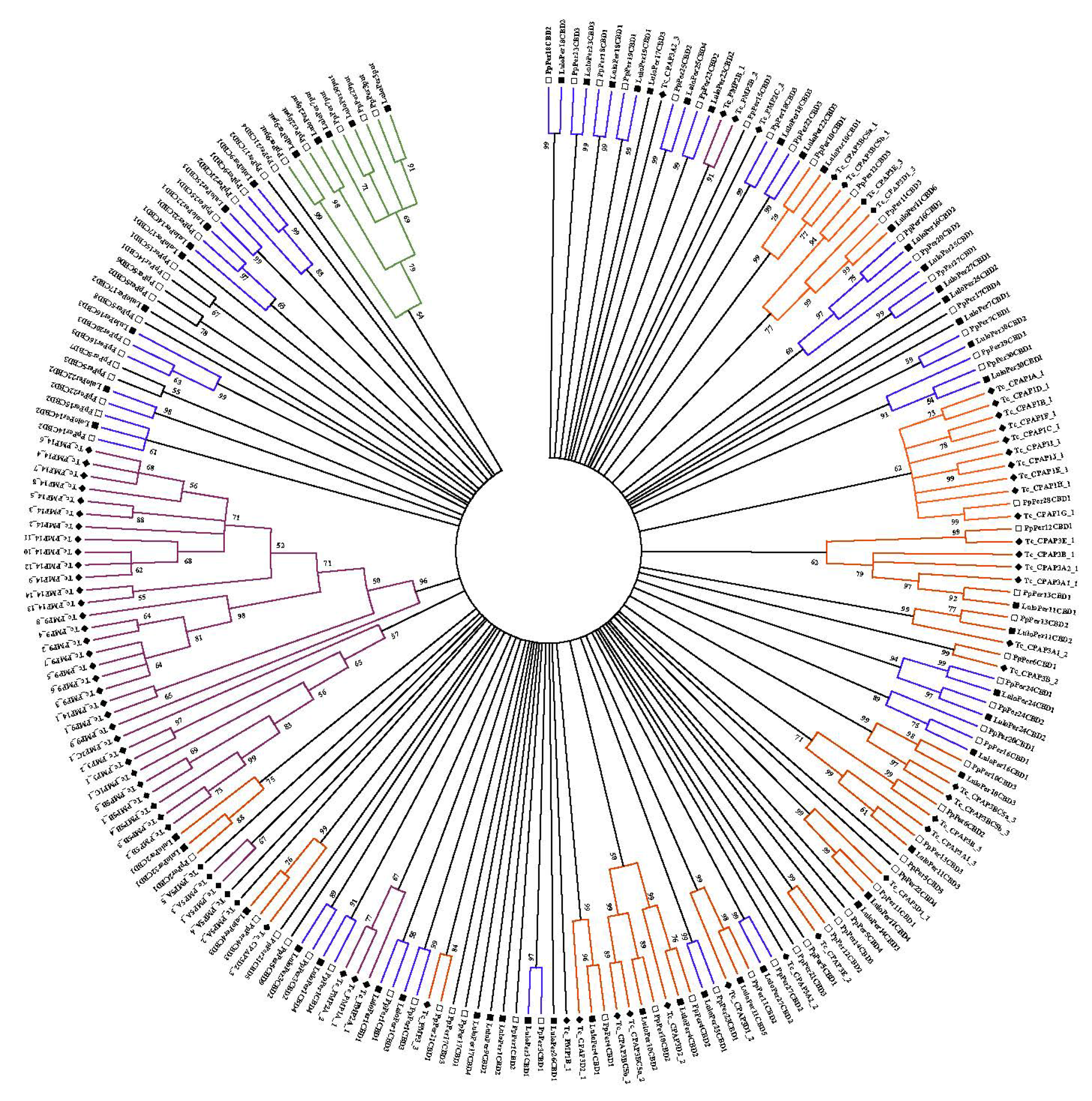

Supplement: S5 Fig — Open squares, filled squares, and filled diamonds represent Ph. papatasi, Lu. longipalpis, and T. castaneum domains, respectively. Branches exclusive to T. castaneum were color-coded in magenta; those specific to sand flies were highlighted in blue. The branch encompassing the CBD-like domain “CBDput” is highlighted in green. The branches shared by sand flies and RFB CBD domains are color-coded in orange. Maximum likelihood tree was constructed using the Whelan and Goldman (WAG) model with Gamma distributed among Invariant sites (G+I), as suggested by the Model test function of the Mega6 software. One thousand bootstrap replicates were performed, and only branches displaying at least 50% confidence are shown. (TIF) [file pntd.0010862.s041.tif]

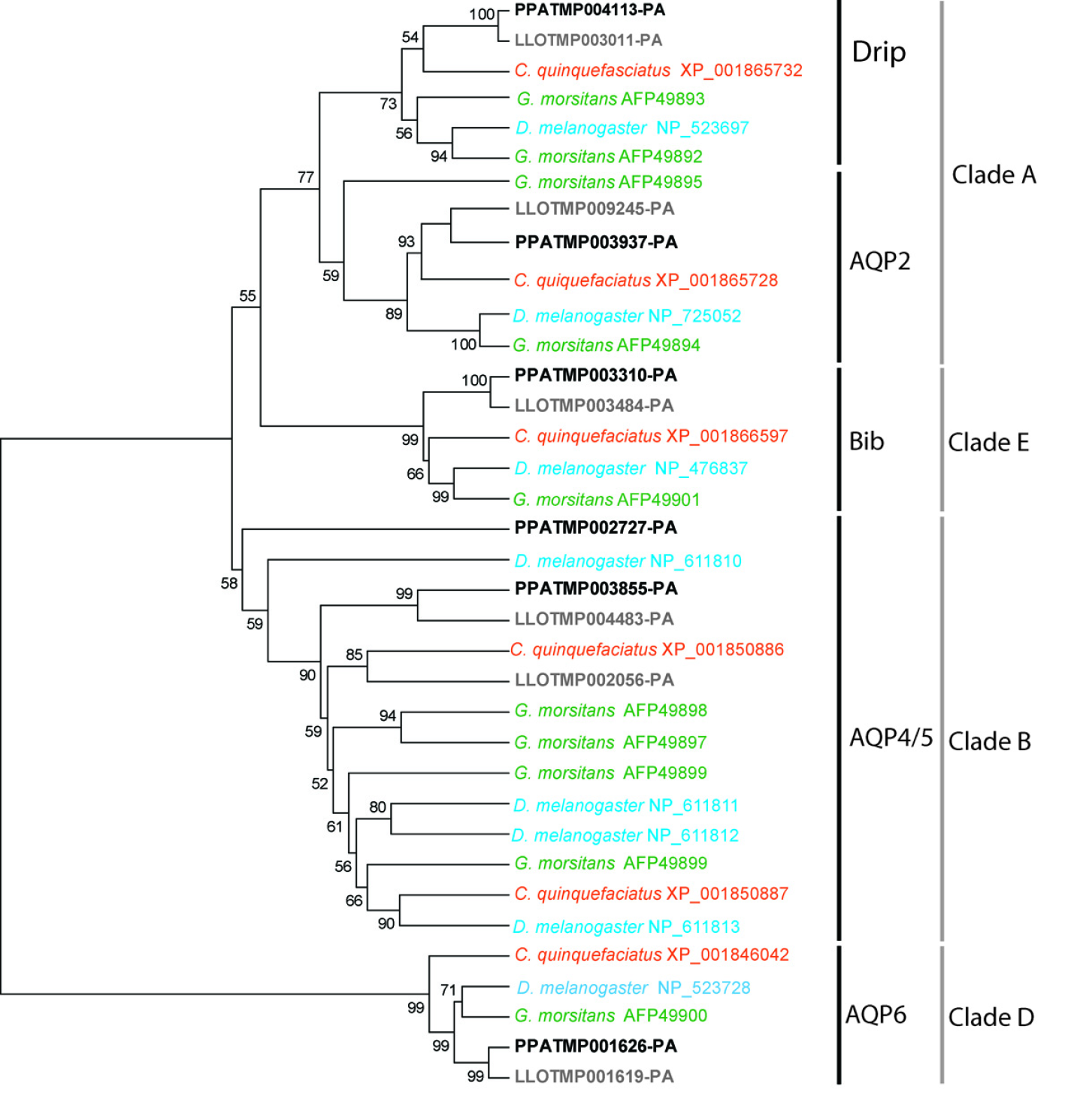

Supplement: S6 Fig — Neighbor-joining tree was produced using MEGA6 using Dayhoff Model and pairwise matching; branch values indicate support following 3000 bootstraps; values below 50% are omitted. (TIF) [file pntd.0010862.s042.tif]

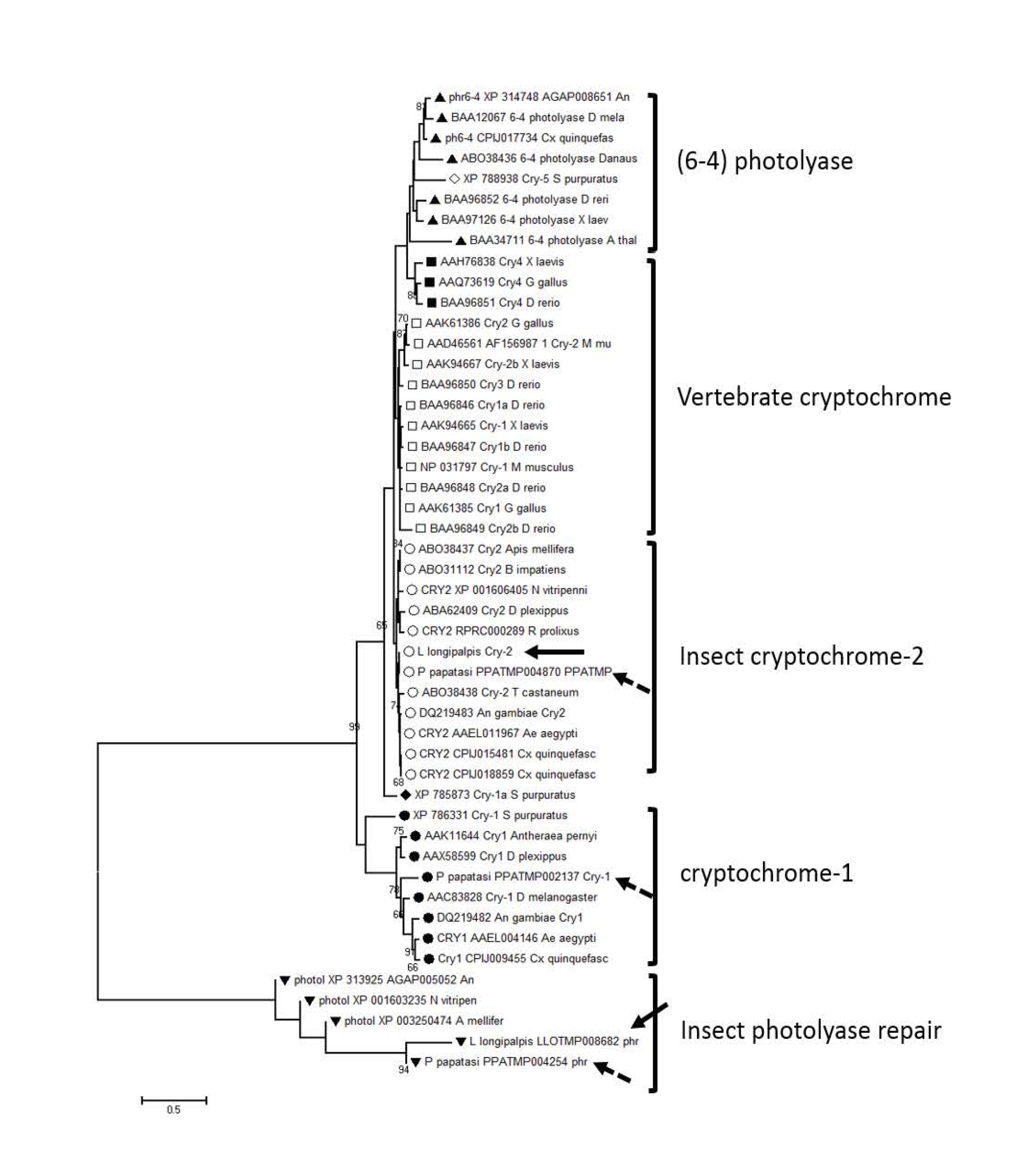

Supplement: S7 Fig — The different photoyases are displayed on the right. The evolutionary history was inferred by using the Maximum Likelihood method based on the Jones-Taylor-Thorton + four gamma categories with 1000 bootstrap replicates (showing only above 65). Sequences with squares are vertebrate cryptochromes (black—cry-4, white—cry-1, cry-2, and cry-3); sequences with black traingles represent (6–4) insect photolyases; sequences with inverted black triangles are reprenting all insect photolyase repir proteins; and sequences with a dot symbol show insect cryptochromes (black–cry-1, white–cry-2). Dashed arrows point to Ph. papatasi photolyase sequences and straight arrows to Lu. longipalpois photolyase sequences. (TIF) [file pntd.0010862.s043.tif]

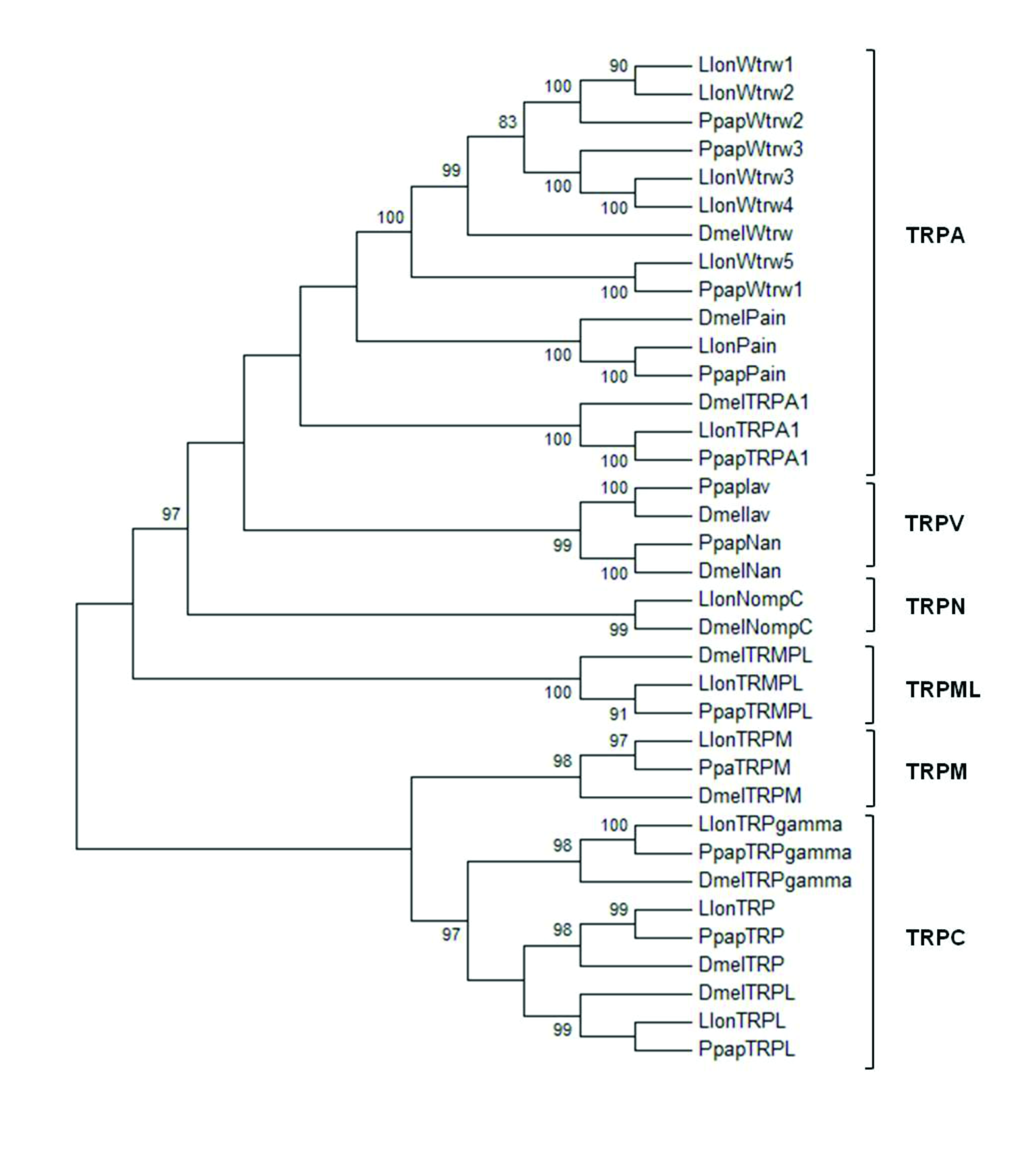

Supplement: S8 Fig — The different TRP subfamilies are displayed on the right. The evolutionary history was inferred by using the Maximum Likelihood method based on the Whelan and Goldman +Freq. model with 1000 bootstrap replicates. (TIF) [file pntd.0010862.s044.tif]

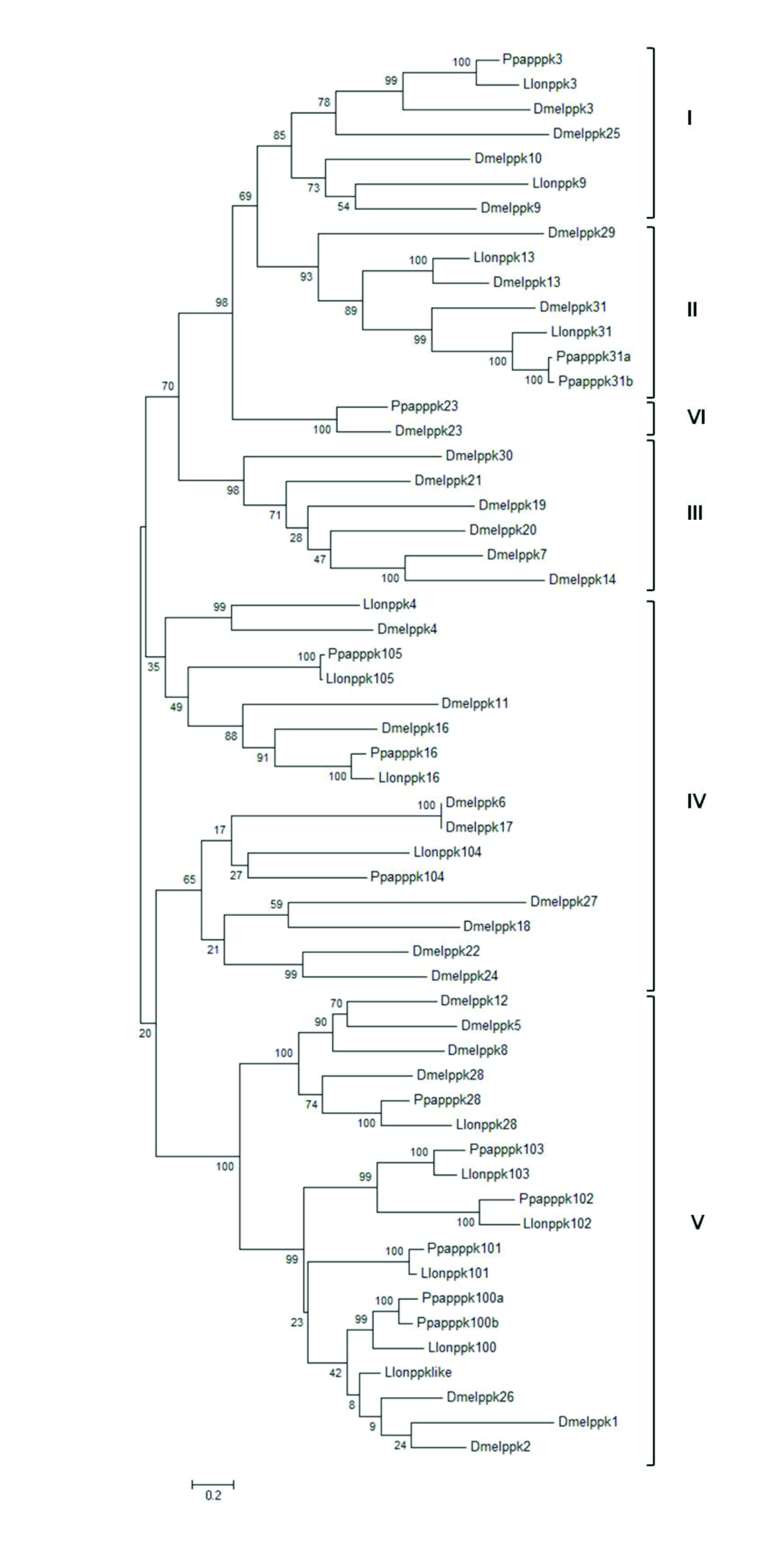

Supplement: S9 Fig — The different PPK subfamilies are displayed on the right. The evolutionary history was inferred by using the Maximum Likelihood method based on the Whelan and Goldman +Freq. model with 1000 bootstrap replicates. (TIF) [file pntd.0010862.s045.tif]

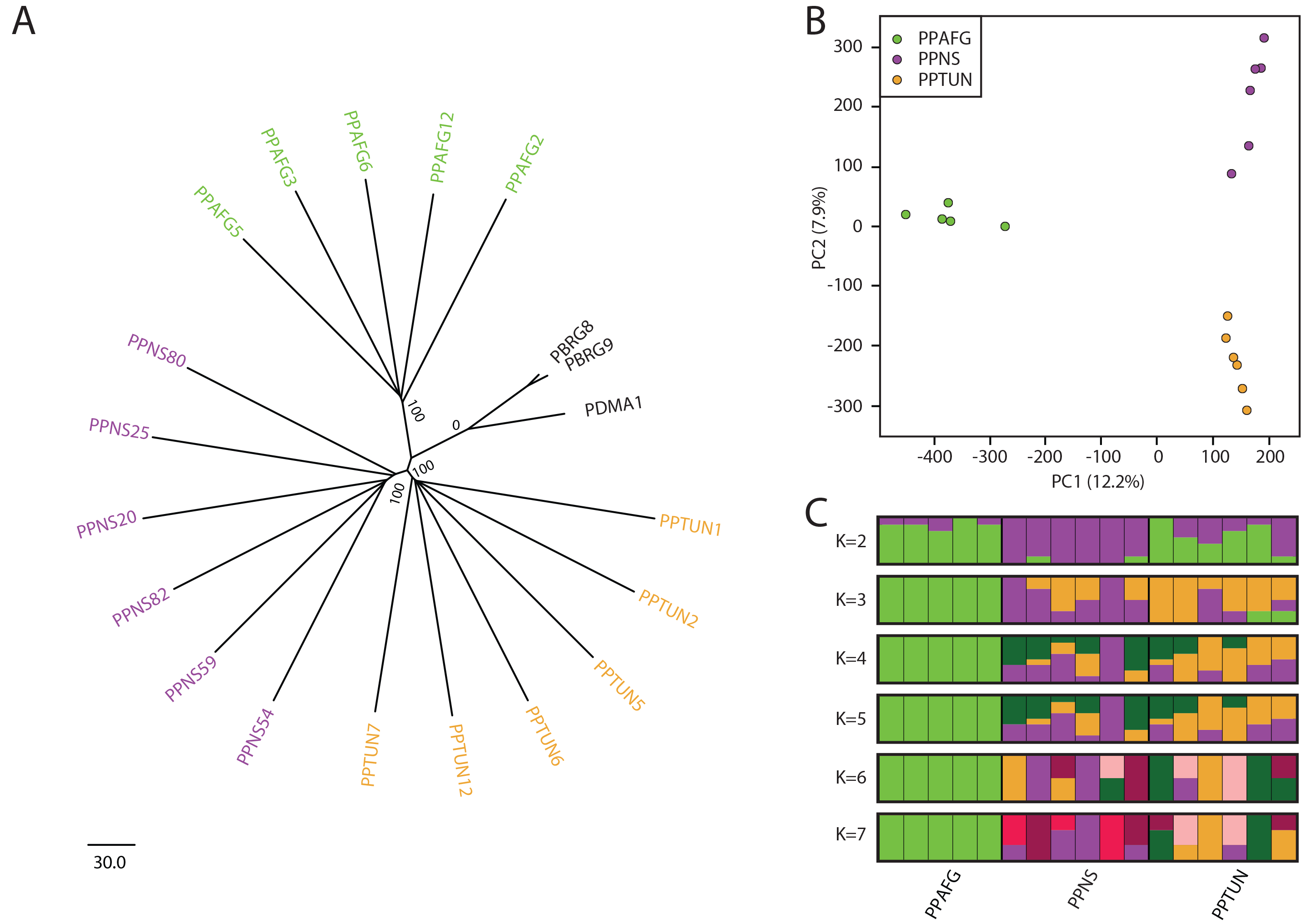

Supplement: S11 Fig — Inferred population structure of Ph. papatasi individuals collected from Afghanistan (PPAFG; green), North Sinai—Egypt (PPNS; purple), and Tunisia (PPTUN; orange). (A) Phylogenetic Analysis. Rooted neighbor joining (NJ) radial tree generated with the Adegenet and ape packages of R. We included both Ph. bergeroti (PBRG; black) and Ph. duboscqi (PDMA; gray), and used Ph. duboscqi to root the trees. Bootstrap values represent the percentage of 1,000 replicates. (B) Principle component analysis (PCA). Individuals are plotted according to their coordinates on the first two principal components (PC1 and PC2). (C) Admixture analysis. Ancestry proportions for Admixture models from K = 2 to K = 7 ancestral populations. Each individual is represented by a thin vertical line, partitioned into K coloured segments representing the individual’s estimated membership fractions to the K clusters. These data are the average of the major q-matrix clusters derived by CLUMPAK analysis. (TIF) [file pntd.0010862.s047.tif]

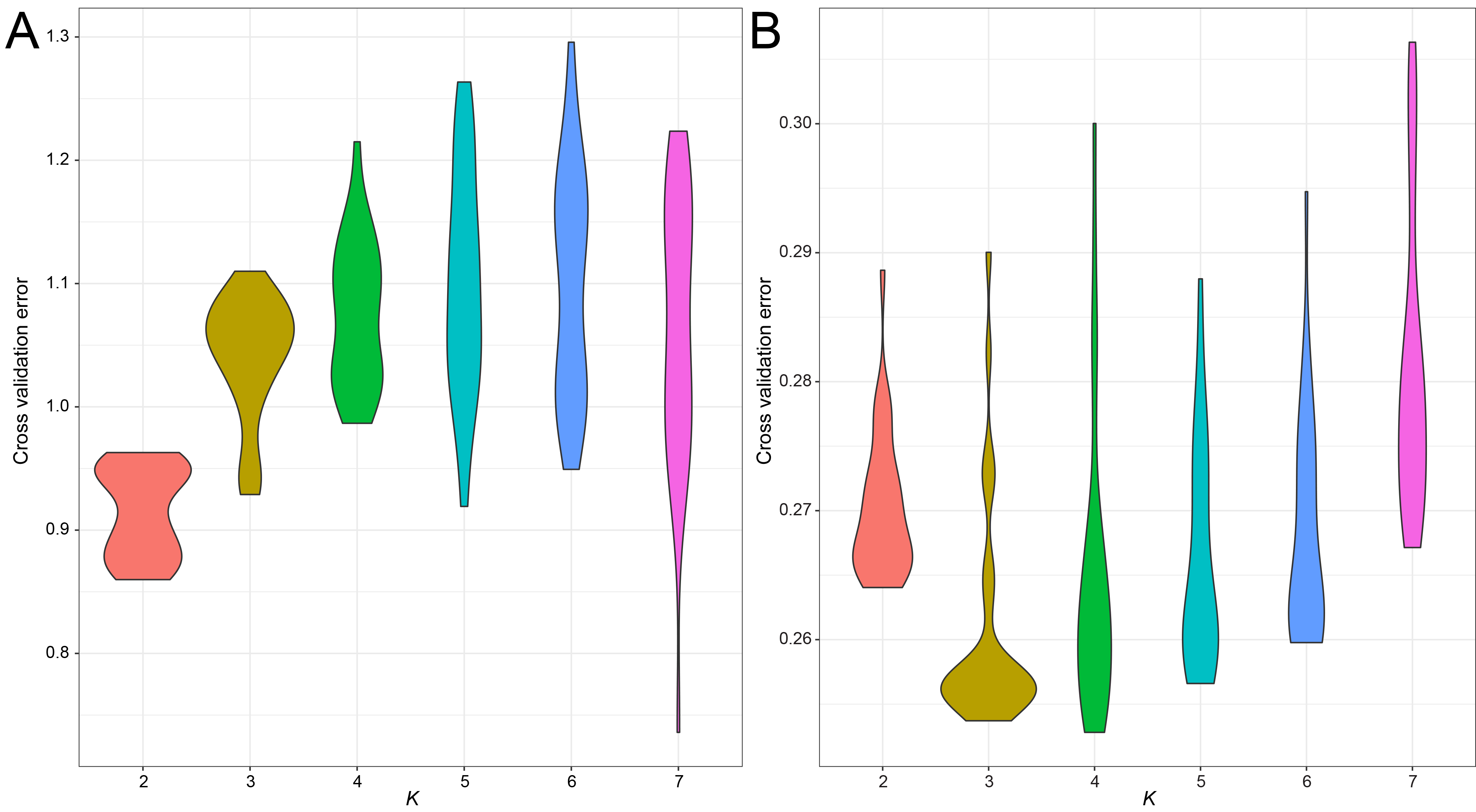

Supplement: S12 Fig — Violin plot of the cross-validation error for each of 30 replicates for each K value. (A) Phlebotomus papatasi populations. (B) Lutzomyia longipalpis populations. (TIF) [file pntd.0010862.s048.tif]

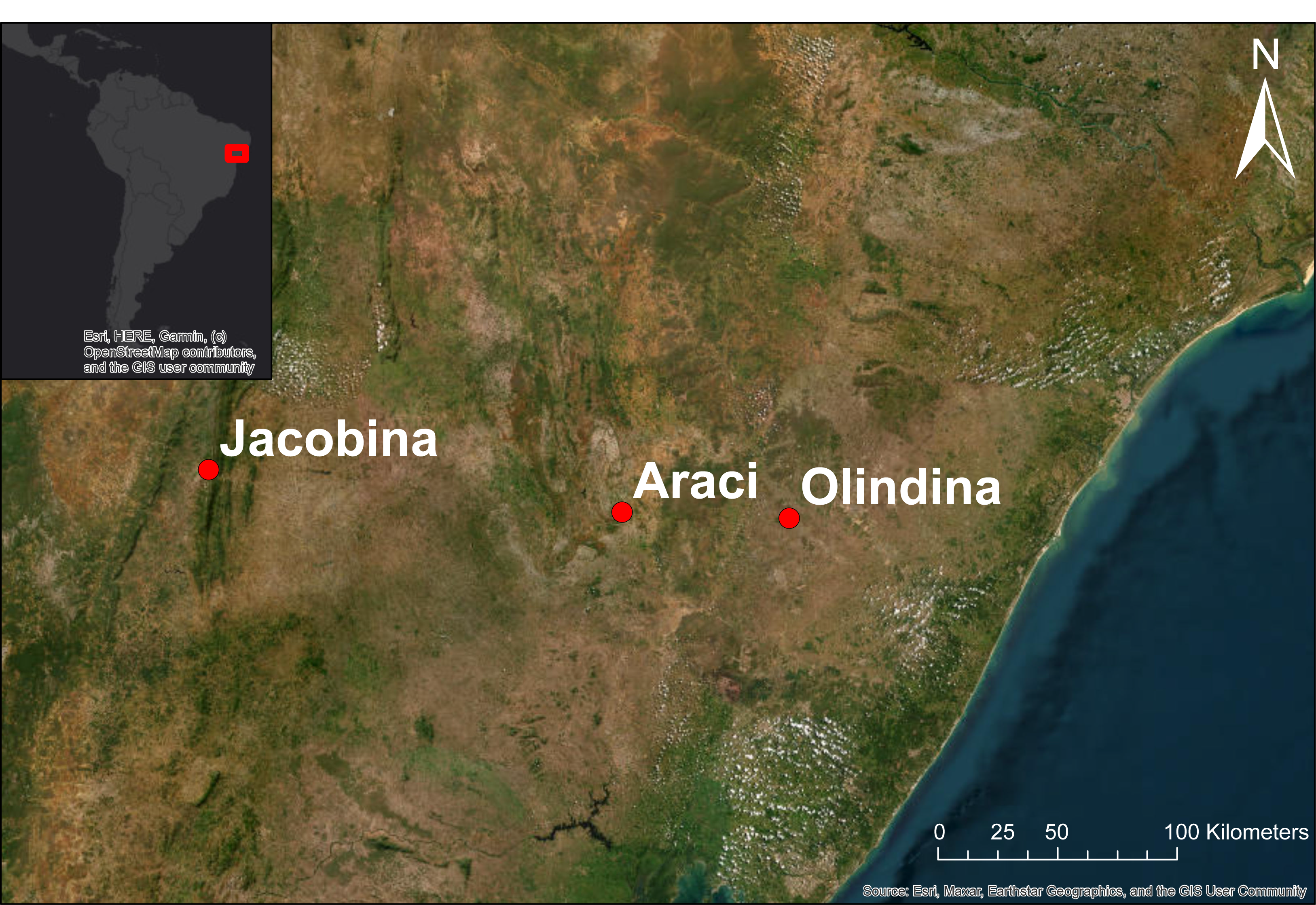

Supplement: S13 Fig — (A) Approximate distance of Araci and Olinda from Jacobina (B). Male copulatory courtship song tracings of Lutzomyia longialpis males collected from Araci and Olindina. The figure shows ~1 s of song in each case. Main map source: World Imagery (Source: Esri, Maxar, Earthstar Geographics, and the GIS User Community; http://goto.arcgisonline.com/maps/World_Imagery). Inset map source: World Dark Gray Canvas Base (Esri, HERE, Garmin, OpenStreetMap contributors, and the GIS user community; http://goto.arcgisonline.com/maps/Canvas/World_Dark_Gray_Base). (TIF) [file pntd.0010862.s049.tif]

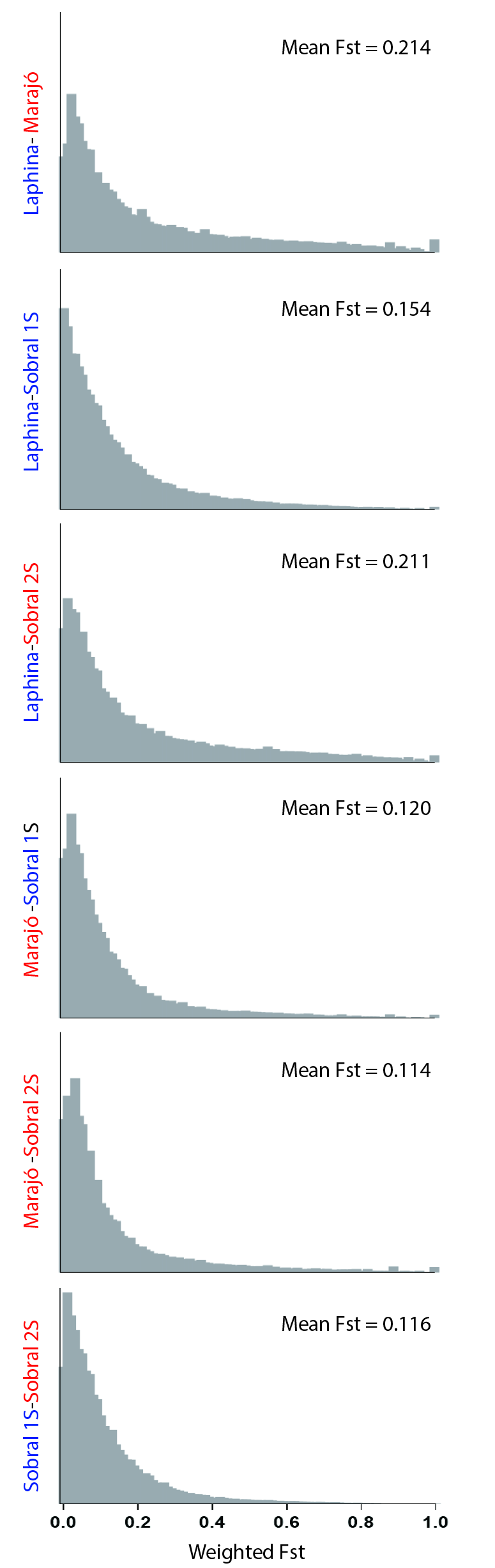

Supplement: S14 Fig — Weighted FST values for 1kb non-overlapping windows were calculated across the genome for each population comparison. (TIF) [file pntd.0010862.s050.tif]

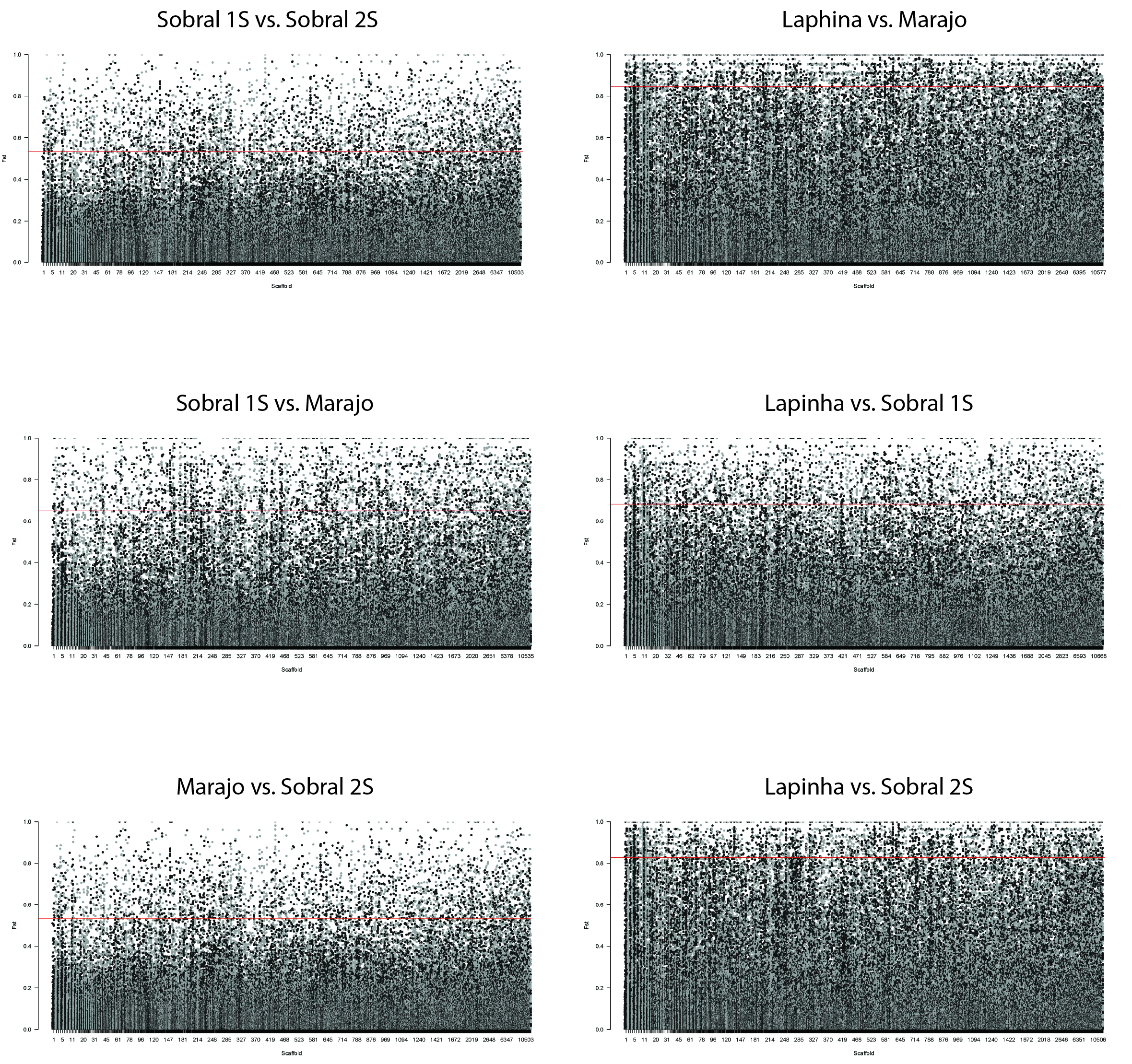

Supplement: S15 Fig — The red horizontal lines indicate the upper 0.05% of FST distribution over the entire genome. (TIF) [file pntd.0010862.s051.tif]

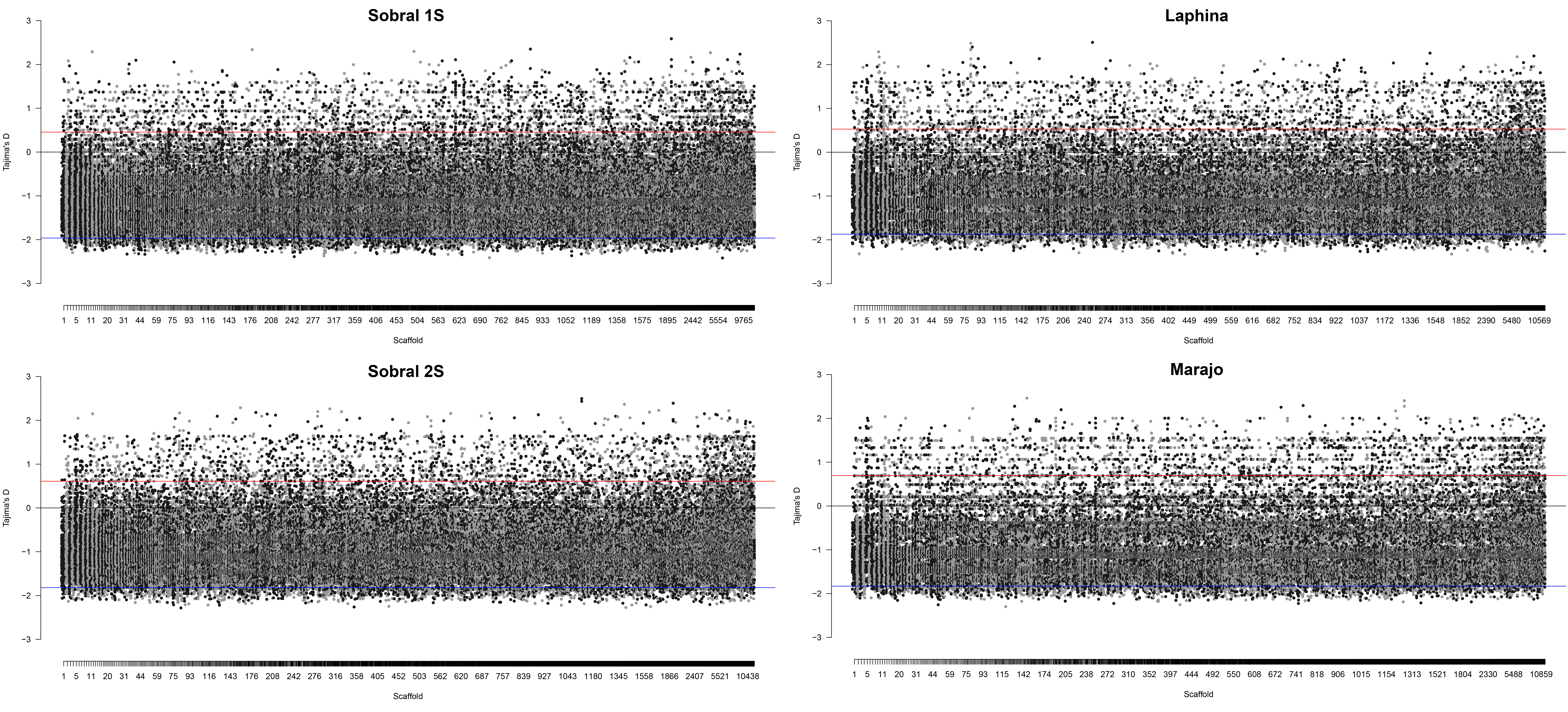

Supplement: S16 Fig — The red and blue horizontal lines indicate the upper and lower 0.05% of Tajima’s D distribution, respectively. (TIF) [file pntd.0010862.s052.tif]

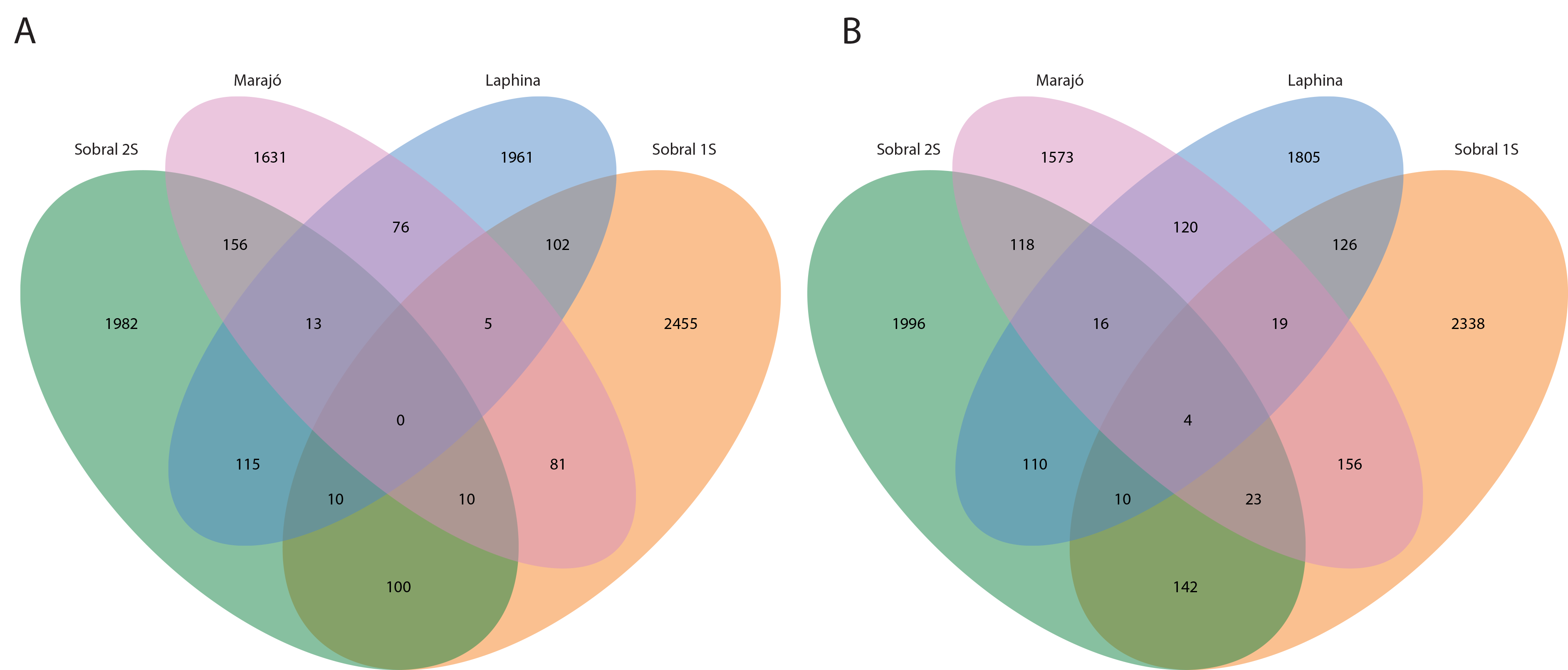

Supplement: S17 Fig — (A) The Venn diagram summarizes the numbers of 1kb genomic windows with Tajimas’D values in the upper 2.5% of the different populations. (B) The Venn diagram summarizes the numbers of 1kb genomic windows with Tajimas’D values in the lower 2.5% of the different populations. (TIF) [file pntd.0010862.s053.tif]
