# Supplementary material for: Genomic analysis of two phlebotomine sand fly vectors of Leishmania from the New and Old World
Source: PLoS Negl Trop Dis. 2023 Apr 12;17(4):e0010862. doi: 10.1371/journal.pntd.0010862 (PMC10138862; doi:10.1371/journal.pntd.0010862)
Supplement: Attachment — Submitted filename: Review of Manuscript PNTD-D-22-01257.docx [file pntd.0010862.s056.docx]

Review of Manuscript Number: PNTD-D-22-01257. Genomic Analysis of Two Phlebotomine Sand Fly Vectors of Leishmania from the New
and Old World

Summary:

Recommendation: acceptance without revision

This paper is a significant contribution to the study, not only of the phlebotomine sandflies, but also the wider group of dipteran disease vectors. The authors are to be congratulated on their success with these very challenging genome assemblies, and the extensive analysis of the genomic data. Personal highlights for me include the treatment of transposable element families and circadian rhythm genes, and also immunity genes (my current rabbit-hole in the family that I work with). The inclusion of natural population samples is a valuable enhancement to the analysis.

I hesitate to suggest more work that might delay publication, because I believe this paper will be valuable for other members of the dipteran genome community. However, if the authors might find it helpful to include the Chironomidae in their orthology analysis. Several chironomids genomes are available in OrthoDB (e.g., *Clunio*). Chironomids are members of the Culicimorpha, as sister family to the Simuliidae (blackflies). Although they are not vectors, having a non-mosquito member of the Culicimorpha might help strenghthen the clustering of the phlebotomines with the Culicimorphs as opposed to the advanced dipterans. However, I certainly would not make acceptance contingent on inclusion of chironomids.
