# Supplementary material for: Genomic analysis of two phlebotomine sand fly vectors of Leishmania from the New and Old World
Source: PLoS Negl Trop Dis. 2023 Apr 12;17(4):e0010862. doi: 10.1371/journal.pntd.0010862 (PMC10138862; doi:10.1371/journal.pntd.0010862)
Supplement: Attachment — Submitted filename: Response to Reviews.docx [file pntd.0010862.s057.docx]

**Response**

We appreciate the assistance of the editorial staff regarding the review of our laboratory’s manuscript. Moreover, we appreciate time and efforts by the reviewers in submitting thoughtful critiques of the manuscript and are delighted by the positive response. We have revised the manuscript to address the reviewer’s concerns specifically and feel that the revisions inspired by the critiques have led to an improved manuscript. We have highlighted our responses to the reviews in red below.

In the revision process, we made substantial changes to the introduction section that describes sand fly copulatory songs and pheromones and minor editorial changes throughout the document. These changes are reflected in the track changes version of the document. Additionally, we discovered that we forgot to include and author in the original submission, Gordon Hamilton, and were able to contact an author, Patrick Minx, that we had only included in the acknowledgement section in the original submission. Both investigators have now been included as authors.

**Reviewer comments:** 

**Methods**

Reviewer #1: The methods are appropriate and described in sufficient detail to be reproducible. I see no difficulties as written.

No action needed.

Reviewer #2: -Line 246. It is a stretch to say that DNA was sourced from inbred insects when no effort was made to make an inbred line. Other groups at the time performed several generations of full sibling single brother/sister matings to make an inbred line. Any claim to using an inbred strain should be removed. To say the DNA was sourced from a lab strain that may have reduced heterozygosity as the colony fluctuated in colony size over time would be acceptable.

We removed the word “inbred” and altered later sentence to read “At several times since establishment in the laboratory, the colony has fluctuated in population size and has been expanded from a relatively low number of files, therefore, this colony may have reduced heterozygosity. Sand flies were reared by the method of Modi and Tesh [54].” (starting on line 280 in edited, non-track changes version)

-Paragraph beginning line 315. Quite a few details on the genome assembly that would normally be included are missing here. How were contaminants removed? Cite method used.

We added additional information about how the contaminants were removed by including the sentence “Prior to submission to NCBI, this assembly was screened for contamination as previously described [55] by using MegaBLAST [56] against bacterial and vertebrate genome databases, resulting in the removal of 247 contigs.” (starting on line 347 in edited, non-track changes version).

Citations for the in the in-house “PolyGraph assembler” and “PyGap” are missing. Please add or provide more details on the software. They should be made available (e.g. GitHub).

Unfortunately, the code for both of the McDonnell Genome Institute (Washington University) software utilized was discontinued, relied on other in-house software programs, and was never published. We have provided information on what the programs did and provided citations for other studies that utilized the PyGap algorithm. The section now reads “Heterozygous contigs were removed or merged reducing the assembled genome size from 364 Mb to 345 Mb. A total of 5,661 gaps were closed and nearly 6.8 Mb of sequence was added using PyGap as previously described [55, 57-59]. The PyGap program utilizes the Pyramid assembler to detect and merge overlaps of adjoining contigs and closes gaps between non-overlapping adjoining contigs with Illumina data. The same Illumina data used in gap closure was aligned to the assembly to correct 89,378 presumed 454 insertion/deletion errors.” (starting on line 349 in edited, non-track changes version).

Illumina data was used for gap closure. but where did this come from? Only Sanger and 454 reads are mentioned in the preceding paragraph.

We apologize that the sentence describing the Illumina data was inadvertently omitted. We have now included the sentence “Whole-genome shotgun Illumina paired-end reads (300 bp inserts) were sequenced to 20X coverage for gap closing.” (starting on line 342 in edited, non-track changes version).

-line 353. List the stages sourced for RNAseq and ages of adults.

We have added additional information to describe the information requested. The sentence now reads, “To generate an extensive RNA-seq coverage to allow for quality gene prediction, RNA was obtained from both sexes one-, three-, and ten-days post emergence, during development, and females post feeding (6, 24, and 96 hours for *Ph. papatasi* and 6, 24, and 144 hours for *Lu. longipalpis*) on uninfected and *Leishmania* [*Le. major* (MHOM/IL/81/Friedlin) for *Ph. papatasi* and *Le. infantum* (MHOM/BR/76/M4192) for *Lu. longipalpis*] infected mouse blood.” (starting on line 385 in edited, non-track changes version).

**Results**

Reviewer #1: The Results are clearly presented, with appropriate supporting Figures.

No action needed.

Reviewer #2: _lines 522-528. N50s of 28kb and 85 kb and BUSCO scores of 86% are quite poor by current standards. Very fragmented genomes that are likely missing regions of the genomes.

We agree with the reviewer and have added the following sentence to alert readers to the possibility that regions are missing: “The N50 sizes and BUSCO completeness scores suggest that the assemblies are fragmented and may be missing regions of the genomes.” (starting on line 535 in edited, non-track changes version).

-orthology. 36 dipteran species sounds impressive but it is mostly a comparison of the two sandfly genomes with Drosophila and Anopheles genomes. So, not a broad sampling of Diptera. This was likely done at least 8 years ago. My suggestion would be to redo this analysis with a broader sampling of Dipteran genomes and not such a heavy reliance on Drosophila and Anopheles.

Response to broaden dipteran sampling (Reviewer #2) and optional suggestion to include chironomids (Reviewer #1, Recommendation section below), we agree that with the current rate of new genomes coming online there are now several additional dipteran species that could be included in our orthology and phylogenomics analyses, however, redoing the analysis every time a new genome appears in the public databases is considerable work and is unlikely to add substantially to our findings. The potential biases noted by Reviewer #2 were addressed by carefully selecting species for the comparative analyses to have a completely symmetrical phylogeny of flies and mosquitoes (see Figure 2: Molecular species phylogeny and ortholog sharing), comprising four anophelines and *Culex* for the mosquitoes and four drosophilids and *Glossina* for the flies.

-line 564. My recommendation would be to delete this section. With the current quality of reference genomes, similar synteny analyses use long chromosome-length scaffolds. Perhaps microsynteny could be reported but I would think this would be better left for the next versions of the genomes of these species.

As suggested by the reviewer, we removed all sections, including S2 Fig. and S4 Table, referring to synteny analysis. Note: remaining figures and tables were renumbered accordingly.

-TEs. With such fragmented genomes are the authors confident they can accurately report the % of the genomes that are repetitive? Long reads would definitely help with the assembly of regions with blocks of repetitive DNA. Consequently, speculation on the possible expansion of TEs in one species would seem premature.

We agree with the reviewer and have added a qualifying statement to the sentence. The sentence now reads, “Although the fragmented nature of the genome assemblies makes a completely accurate assessment of TE content difficult, the comparison of the TE content in both sand fly genome assemblies suggest an expansion of all the TE classes and orders in the *Ph. papatasi* genome.” (starting on line 589 in edited, non-track changes version).

Also, it is class II, the DNA transposons, that are “copy and paste” not class I, retrotransposons.

Class II TEs, the DNA transposons, are referred to as “cut-and-paste” and the class I elements are referred to as “copy-and-paste” (<https://doi.org/10.1186/s13059-018-1577-z>) therefore, we have left the section as originally written.

-Circadian Rhythm Genes. Perhaps Lu. longipalpis has only one CRY gene but with such highly fragmented genomes, this is not particularly convincing. Is this supported by RNAseq data? This section could be deleted.

We agree that it is possible that the fragmented *Lu. longipalpis* assembly may have made it impossible to identify *CRY1*. The sentence in the original submission contained a qualifying phrase “…, is just missing in the current assembly”. We have now added the word “fragmented” to further highlight the qualification. The sentence now reads, “We can speculate that the possible loss of *CRY1* in *Lu. longipalpis* genome may be related to a better adaptation of these insects to living in caves and dark places or alternatively, is just missing in the current fragmented assembly.” (starting on line 644 in edited, non-track changes version).

-Chemosensory Receptors. Wasn’t this already published? If nothing new here this section could be deleted.
As suggested, we removed S22 Table that reported the chemosensory receptor data that has been previously published. However, we felt that it would be helpful to readers to have some summary information regarding the previously published analysis on olfactory, gustatory, and ionotropic receptors so reworded the section to read, “The sand fly olfactory receptor (OR), gustatory receptors (GR), and ionotropic receptor (IR) repertoires were published elsewhere [35]. The sand fly OR repertoires in the genome assemblies comprise 139 canonical ORs in *Lu. longipalpis* and *Ph. papatasi*, plus one copy each of the odorant receptor co-receptor, *Orco*. Eighty-two genes encoding 91 GRs in *Lu. longipalpis* and 77 genes encoding 88 GRs in *Ph. papatasi* were identified in the reference assemblies, and 23 and 28 IR genes in *Lu. longipalpis* and *Ph. papatasi*, respectively were identified. Three ORs and three IRs suspected to be missing in the *Lu. longipalpis* references assembly were found in *de novo* assemblies of the field isolates [35].” (starting on line 649 in edited, non-track changes version).

-No comments on the population structure section other than I thought it was quite interesting and suggests follow-up studies are needed in Jacobina given the data suggests there are two groups.

We agree with the reviewer that the data is quite interesting and look forward to following up on the data in the future.

**Conclusions**

Reviewer #1: (No Response)

Reviewer #2: OK

No action needed.

**Recommendation and Editorial and Data Presentation Modifications**

Reviewer #1: Recommendation: acceptance without revision

I hesitate to suggest more work that might delay publication, because I believe this paper will be valuable for other members of the dipteran genome community. However, if the authors might find it helpful to include the Chironomidae in their orthology analysis.  Several chironomids genomes are available in OrthoDB (e.g., Clunio).  Chironomids are members of the Culicimorpha, as sister family to the Simuliidae (blackflies). Although they are not vectors, having a non-mosquito member of the Culicimorpha might help strengthen the clustering of the phlebotomines with the Culicimorphs as opposed to the advanced dipterans (or perhaps change the topology to the contrary!).  However, I certainly would not make acceptance contingent on inclusion of chironomids.

Please see “Results/Reviewer #2/-orthology” response above.

Reviewer #2: -Paragraph beginning line 211. The transition from blood meal digestion to circadian rhythms did not flow well. I realize that this sets up the finding that one sandfly species may only have one CRY gene but this deserves a separate paragraph. Or this could be removed entirely (see comments on results)

As suggested by the reviewer, we made a separate paragraph for this section. The section now reads “Many important aspects in sand fly biology such as hematophagy and host seeking are controlled by the biological clock [48]. In *Lu. longipalpis*, the main clock genes and their expression pattern throughout the day have been previously characterized [49, 50]. However, the molecular regulation of circadian rhythms is poorly understood in sand flies. Yuan *et al.* 2007 proposed three clock models based on the presence of the cryptochrome (CRY) proteins, CRY1 and CRY2 [51]. In the *Drosophila* clock model, only CRY1, which acts as a blue-light photoreceptor [52], is present. In the butterfly model, CRY1 also acts as a photoreceptor and CRY2, which is a mammalian–like transcriptional repression, dimers with PER to repress CLK/CYC activity. In the bee model, there is only CRY2, which seems to act as a repressor together with PER and some other molecule that is not CRY1 that acts as photoreceptor.” (starting on line 245 in edited, non-track changes version).

**Summary and General Comments**

Reviewer #1: This paper is a significant contribution to the study, not only of the phlebotomine sandflies, but also the wider group of dipteran disease vectors.  The authors are to be congratulated on their success with these very challenging genome assemblies, and the extensive analysis of the genomic data.  Personal highlights for me include the treatment of transposable element families and circadian rhythm genes, and also immunity genes (my current rabbit-hole in the family that I work with).  The inclusion of natural population samples is a valuable enhancement to the analysis.

We thank the reviewer for their kind sentiments.

Reviewer #2: It can take a long time from the completion of a genome assembly to publication but not usually a decade! The genome assemblies for the two sandfly species were completed in 2012 from a mix of Sanger and 454 reads! The DNAs were sourced from lab strains that had undergone bottlenecks but were not deliberately inbred for multiple generations such as was done for other insect genome projects at the time. Consequently, the assemblies are highly fragmented and not what would be considered reference genomes in 2022. Most insect genome papers I have seen this year use long reads such as PacBio HiFi and use HiC to obtain chromosome-length scaffolds. While the genome assemblies do not meet current standards for reference genomes, the manuscript is important because of the extensive efforts the authors have made at manual annotation and use of the assemblies for an analysis of population structures. A huge amount of valuable information has been collated in the 36 supplementary tables and 18 supplementary figures. I mostly have a few minor comments that should be addressed and one request. My request is that, if not done already, that all the manual annotations be submitted to NCBI. Too often I find insect genomes submitted to NCBI with only the annotations generated by MAKER (or similar) with the manual annotations only provided in the supplementary data of the paper.

We agree that the methods utilized to generate the reported assemblies did not utilize the most current techniques and appreciate that, nonetheless, both reviewers feel that the results provide valuable information to the scientific community.

Most of the manual annotations have been incorporated into the annotation hosted on VectorBase as indicated by the ID numbers presented in the tables. VEuPathDB/VectorBase (corresponding author McDowell is PI of VEuPathDB) are currently working closely with NCBI to develop automated methods whereby the annotations made in the VEuPathDB family of databases (including VectorBase) will be reflected in the NCBI database.
